# Species Discrimination within the *Metarhizium* PARB Clade: Ribosomal Intergenic Spacer (rIGS)-Based Diagnostic PCR and Single Marker Taxonomy

**DOI:** 10.3390/jof9100996

**Published:** 2023-10-08

**Authors:** Christina Schuster, Yamilé Baró Robaina, Haifa Ben Gharsa, Saikal Bobushova, Romina Guadalupe Manfrino, Alejandra C. Gutierrez, Claudia C. Lopez Lastra, Tinatin Doolotkeldieva, Andreas Leclerque

**Affiliations:** 1 Department of Biology, Technische Universität Darmstadt (TUDa), Schnittspahnstraße 10, 64287 Darmstadt, Germany; robainabaro@gmail.com (Y.B.R.);; 2 Plant Health Research Institute (INISAV), 110 Str. 514, Havana 11600, Cuba; 3 Faculty of Agriculture, Kyrgyz-Turkish Manas University, 56 Chyngyz Aitmatov Avenue, Bishkek 720038, Kyrgyzstan; 4 Centro de Estudios Parasitólogicos y de Vectores (CEPAVE), CONICET-Consejo Nacional de Investigaciones Científicas y Técnicas, UNLP-Universidad Nacional de La Plata, La Plata 1900, Argentina

**Keywords:** *Metarhizium anisopliae*, *Metarhizium robertsii*, *Metarhizum pinghaense*, *Metarhizium brunneum*, PARB clade, ribosomal intergenic spacer (rIGS), species delineation, diagnostic PCR, single-marker phylogeny

## Abstract

(1) Background: The entomopathogenic fungus *Metarhizium anisopliae sensu lato* forms a species complex, comprising a tight cluster made up of four species, namely *M. anisopliae sensu stricto*, *M. pinghaense*, *M. robertsii* and* M. brunneum*. Unambiguous species delineation within this “PARB clade” that enables both the taxonomic assignment of new isolates and the identification of potentially new species is highly solicited. (2) Methods: Species-discriminating primer pairs targeting the ribosomal intergenic spacer (rIGS) sequence were designed and a diagnostic PCR protocol established. A partial rIGS sequence, referred to as rIGS-ID800, was introduced as a molecular taxonomic marker for PARB species delineation. (3) Results: PARB species from a validation strain set not implied in primer design were clearly discriminated using the diagnostic PCR protocol developed. Using rIGS-ID800 as a single sequence taxonomic marker gave rise to a higher resolution and statistically better supported delineation of PARB clade species. (4) Conclusions: Reliable species discrimination within the *Metarhizium* PARB clade is possible through both sequencing-independent diagnostic PCR and sequencing-dependent single marker comparison, both based on the rIGS marker.

## 1. Introduction

The fungal genus *Metarhizium* (Hypocreales; Clavicipitaceae) comprises a wide range of insect pathogens of high interest for fundamental research in both host–pathogen interaction and the development of microbial insecticides [1,2]. The findings that *Metarhizium* fungi infect and kill economically important insect pests [3,4,5,6,7], colonize the plant rhizosphere [8,9,10] and can be produced and formulated with comparative ease [11,12,13] have triggered the investigation of *Metarhizium* isolates in many parts of the world [14,15,16,17,18,19,20].

A prerequisite for both the scientific investigation and the development of commercial mycoinsecticides is the sound taxonomic identification of fungal strain and isolates. Traditionally, Hypocreales fungi as *Metarhizium* were characterized by microscopic and morphological features such as the appearance and pigmentation of colonies or the shape and dimension of conidia and phialides, leading to the introduction of several *Metarhizium* species such as *Metarhizium anisopliae*, *Metarhizium flavoviride*, *Metarhizium pinghaense* and *Metarhizium guizhouense* [21,22]. The first molecular genetic markers used to delineate *Metarhizium* species were the internal transcribed spacer (ITS) sequence of the ribosomal RNA operon and the gene encoding the large (28S) ribosomal RNA [23]. These studies led inter alia to the introduction of several varieties such as *M. anisopliae *var.* acridum *and var. *lepidiotae* that were later elevated to the rank of taxonomic species [24]. Subsequent studies based on the molecular taxonomy of Hypocreales fungi on a varying set of protein-encoding marker genes of which the genes encode translation elongation factor 1 alpha (EF1A) and the RNA polymerase II subunits 1 and 2 (RPB1 and RPB2, respectively) have gained particular relevance [25,26]. Moreover, the intron-rich 5′-region of the translation elongation factor 1 alpha gene, referred to as 5TEF, has been found to be a suitable marker for species assignment within the genus *Metarhizium* [24]. As a result of respective Multilocus Sequence Analysis (MLSA) studies employing these “legacy genes” [27], the boundaries of the genus *Metarhizium* were redefined [26,28] and a plethora of new *Metarhizium* species were introduced [17,24,29,30,31,32,33]. As a salient feature of the resulting internal structure of the genus, many of the latter are organized into a *Metarhizium anisopliae *and a* Metarhizium flavoviride *species complex [28]. Within the *M. anisopliae* complex, a tight cluster of species informally termed the “PARB clade” according to the species epithets of the four taxa originally included, namely *M. pinghaense*, *M. anisopliae*, *M. robertsii* and* M. brunneum*, comprises fungi of high relevance for both fundamental research and insect biocontrol. Recently, *Metarhizium humberi* has been described as a fifth species within this clade [34,35].

However, these molecular taxonomic studies led to the conclusion that the legacy markers including 5TEF “likely under-represent diversity in the *M. anisopliae* complex” and that additional genetic markers are needed that “extend the resolving power of 5TEF to facilitate species level analyses” [27]. In a respective approach, Kepler and Rehner [27] have identified seven intergenic regions of nuclear genomes across the *M. anisopliae* complex. When used individually as phylogenetic markers, these intergenic loci supported diverging outcomes with respect to the monophyly of PARB species, but, in combination with the 5TEF marker, gave rise to a well-resolved PARB clade [36] and to sound species assignment of new *Metarhizium* isolates within the PARB clade [34].

Together with the intergenic spacer (rIGS) sequence of the ribosomal RNA operon, two of the seven intergenic loci have been developed into a multiplexed PCR assay distinguishing between the PARB clade species [37]. The rIGS sequence had previously been evaluated as a molecular taxonomic marker for *Metarhizium* fungi [38]. In contrast, earlier diagnostic PCR approaches targeting taxa within the *M. anisopliae* complex that were based on the ITS sequence did not discriminate between PARB species [39,40,41].

It was the two-fold purpose of this study (i) to evaluate the ribosomal RNA operon intergenic spacer as a single molecular taxonomic marker of higher resolution of PARB clade species compared to that obtained using 5TEF, and (ii) to develop an rIGS-based diagnostic PCR assay for the sequencing-independent discrimination of the PARB clade species *M. pinghaense*, *M. anisopliae*, *M. robertsii* and* M. brunneum. *If not otherwise stated, the species designation “*M. anisopliae*” refers to *M. anisopliae sensu stricto* throughout this publication.

## 2. Materials and Methods

### 2.1. Fungal Strains and Isolates

*Metarhizium* reference strains and corresponding data used to develop the species-discriminating PCR assay were combined into a “reference data set” as listed in Supplementary Table S1. *Metarhizium* strains and isolates employed to validate the PCR assay for the four PARB species were assembled into a “validation data set” as displayed in Supplementary Table S2. *Metarhizium* strains and isolates used in this study were received from the ARSEF, CEPAVE, INISAV and KTMU culture collections. Fungi were routinely cultivated on Yeast-Peptone-Glucose (YPG) agar (2 g/L of yeast extract, 10 g/L of peptone, 20 g/L of glucose) at 24 °C. Conidia were routinely harvested in sterile 0.005% Tween 20. Single-spore-derived cultures were obtained from environmental isolates by plating serial dilutions of conidial suspensions. Cryo stocks stored at −80 °C were prepared by freezing conidial suspensions with 30% glycerol in liquid nitrogen.

### 2.2. DNA Extraction

For DNA extraction, fungal isolates were grown on YPG agar containing 25 µg/mL of tetracycline for 1–2 weeks. Approximately 100 mg of mycelium was transferred to a screw-capped 2 mL microcentrifuge tube containing Lysing Matrix C (MP Biomedicals, CA, USA). Samples were frozen in liquid nitrogen and processed for 30–60 s at intermediate speed in a Minilys homogenizer (Bertin technologies, Montigny-le-Bretonneux, France). DNA was extracted from homogenized samples using the DNeasy Plant kit (Qiagen, Venlo, Netherlands) according to the standard protocol as provided by the manufacturer using both a Qiashredder and a DNA adsorption column. In the last step, genomic DNA from one mycelial sample was eluted from the column in 100 µL of buffer EB (10 mM Tris-Cl, pH = 8.5) and stored at −20 °C. DNA concentration was determined using a NanoDrop One device (Thermo Scientific, Waltham, MA, USA). Prior to use as diagnostic PCR templates, DNA samples were diluted in sterile 1× TE buffer (10 mM TRIS, 1 mM EDTA, pH 8.0).

### 2.3. Phylogenetic Reconstruction

Nucleotide sequences were aligned using the CLUSTAL W function [42] as implemented in version 11 of the MEGA software package [43]. The Tree-Puzzle version 5.2 software [44] was used to estimate data-set-specific parameters as nucleotide frequencies, the percentage of invariable sites, the transition/transversion ratio and the alpha-parameter for the gamma-distribution-based correction of rate heterogeneity among sites. Two algorithms were used to reconstruct *Metarhizium* phylogenies: (i) the Maximum Likelihood (ML) method as implemented in version 3.1 of the PhyML software tool [45] using the Hasegawa–Kishino–Yano model of nucleotide substitution [46] under the assumption of a gamma-distribution-based model of rate heterogeneity [47] allowing for eight rate categories, and (ii) a p-distance matrix-based Neighbor Joining (NJ) method as implemented in MEGA 11. For both reconstruction methods, alignment gaps and missing data were treated using pairwise deletion, and tree topology confidence limits were explored in non-parametric bootstrap analyses over 1000 pseudo-replicates.

### 2.4. Generation of an rIGS Reference Data Set for Diagnostic PCR Assay Development

Available rIGS sequences from *Metarhizium* PARB species were identified across the Genbank database using the respective nomenclatural type strain sequences as query. For *M. humberi*, due to unavailability of the type strain sequence, the rIGS of strain ESALQ 1638 that had been independently identified in the published genome sequence (GCA_020102295.1) was used as query. All identified rIGS sequences from these species were included in the reference data set (Supplementary Table S1).

Moreover, in order to make the bulk of information on genetic PARB clade diversity available for diagnostic PCR assay development, 5TEF sequences of the nomenclatural type strains representing the four original PARB clade species and *M. humberi* were used as a query in unfiltered BlastN high-similarity searches of the Genbank database, delivering up to 1000 hits as sorted by maximal sequence identity percentages. Integration of the five BlastN search results gave rise to a redundancy-corrected set of 5TEF sequences from PARB clade strains. Sequences were aligned and an NJ tree was generated to be used as guide tree for further reference strain identification. Within this 5TEF phylogeny, main specific sub-clades without presentation in the available rIGS data set were identified. Within these sub-clades, strains available as pure culture or extracted DNA were chosen for rIGS sequencing using oligonucleotide primers and PCR parameters indicated in Supplementary Table S3, and the new rIGS sequences determined were included in the reference data set (Supplementary Table S1).

### 2.5. Design of Primer Pairs for a PARB-Species-Discriminating Diagnostic PCR Assay

All PCR reactions performed in this study, including diagnostic multiplex PCR, were run in a T-One thermocycler (Biometra, Göttingen, Germany) using GoTaq polymerase (Promega, Fitchburg, MA, USA) with dNTP and oligonucleotide primer concentrations of 200 µM and 500 nM, respectively. For the species-discriminating diagnostic PCR assay, 100 pg/µL of extracted genomic DNA was added as PCR template to (typically) 20 µL reactions containing 0.5 units of DNA polymerase. It has been the rationale underlying the diagnostic PCR approach that PARB-species-discriminating primer pairs should be functional under identical reaction conditions. The diagnostic PCR protocol established on the basis of preliminary experimental results consisted of one initial denaturation step of 95 °C for 2 min, 35 cycles of denaturation at 95 °C for 30 s, annealing at 60 °C for 30 s and elongation at 72 °C for 45 s, followed by a 2 min final elongation step at 72 °C.

For primer design, rIGS sequences from the reference data set were carefully aligned and analyzed for species-specific sequence features as Single-Nucleotide Polymorphisms (SNPs) or InDels. Pairs of primers of varying length hybridizing against identified sequence features were optimized under the above fixed reaction conditions against template DNA of *Metarhizium* strains from the reference data set. Diagnostic PCR results were controlled through horizontal gel electrophoresis typically using 1.5% agarose gels stained with 5 µL/100 mL of Roti-GelStain (Carl Roth) in 1× TAE buffer (40 mM TRIS, 20 mM acetic acid, 1 mM EDTA, pH 8.3).

### 2.6. Determination of Molecular Taxonomic Marker Sequences

Molecular taxonomic marker sequences such as EF1A, RPB1, RPB2, 5TEF or rIGS were amplified from extracted genomic DNA, typically in 50 µL reactions, using a generalized preparative PCR protocol consisting of one initial denaturation step of 95 °C for 2 min, 35 cycles of 30 sec at 95 °C, 30 sec at the primer-specific annealing temperature and a 72 °C elongation step of amplicon-specific time, followed by a 5 min final elongation step at 72 °C. Primer sequences as well as amplicon- and primer-pair-specific parameters are indicated in Supplementary Table S3. Formation of a single PCR product of expected apparent size was routinely controlled through horizontal electrophoresis of a 5 µL sample using 1% agarose gels. PCR products were purified using the Qiaquick PCR purification kit (Qiagen) according to the standard protocol provided by the manufacturer comprising the final elution in 50 µL of EB buffer (10 mM Tris·Cl, pH 8.5).

Sanger sequencing of PCR products was performed by StarSEQ (Mainz, Germany) or MicroSynth (Gießen, Germany) using the corresponding PCR primers or additional sequencing primers as indicated in Supplementary Table S3. The raw sequence data obtained were combined into a single consensus sequence for each fungal specimen and marker using version 11 of the MEGA software package. Sequences determined were submitted to the Genbank database under the accession numbers indicated in Supplementary Tables S1 and S2.

## 3. Results

### 3.1. Generation of an rIGS Reference Data Set for Diagnostic PCR Assay Development

Genbank database mining gave rise to a minimal set of 22 published rIGS sequences from PARB clade species, mainly from the study by Mayerhofer et al. [37]. In order to obtain a more representative picture of the known genetic diversity present across the PARB clade, the Genbank database was systematically mined for 5TEF sequences from the four PARB species and *M. humberi*. Redundancy correction of a total of 1333 initially identified sequence entries gave rise to a set of 552 5TEF sequences. When following the species assignments indicated in the respective Genbank datasheets—i.e., *M. pinghaense* (116 sequence entries), *M. anisopliae* (119), *M. robertsii* (138), *M. brunneum* (160), *M. humberi* (2) and no species assignment (14)—the 5TEF data set appeared highly biased against *M. humberi*, presumably reflecting the only recent introduction of this fifth putative PARB clade species. However, in the 5TEF phylogeny reconstructed from this data set (Supplementary Figure S1), the presumed *M. humberi* clade comprised 65 strains that had mostly been assigned to *M. anisopliae,* indicating that the published genetic diversity of *M. humberi* might be much wider than that reflected by explicit strain assignments.

Guided by the 5TEF tree topology, nine further available *Metarhizium* strains were selected from *M. pinghaense (2)*, *M. anisopliae (2)*, *M. robertsii (2)* and *M. brunneum (3)* sub-clades that were not or insufficiently represented in the published rIGS data set. Complete rIGS sequences of these strains were determined and included in the reference data set (Supplementary Table S1). However, as our attempts to obtain extracted DNA or living cultures of recognized *M. humberi* strains were unsuccessful and there was, therefore, no possibility to base diagnostic PCR primer design upon infra-specific rIGS sequence comparison or to validate presumably *M. humberi*-specific primer pairs against cognate template DNA, the present study was limited to the four original PARB species.

### 3.2. Selection of rIGS as Target Sequence for the Development of a Single-Marker Approach to PARB Species Discrimination and Identification

**Figure 1:**
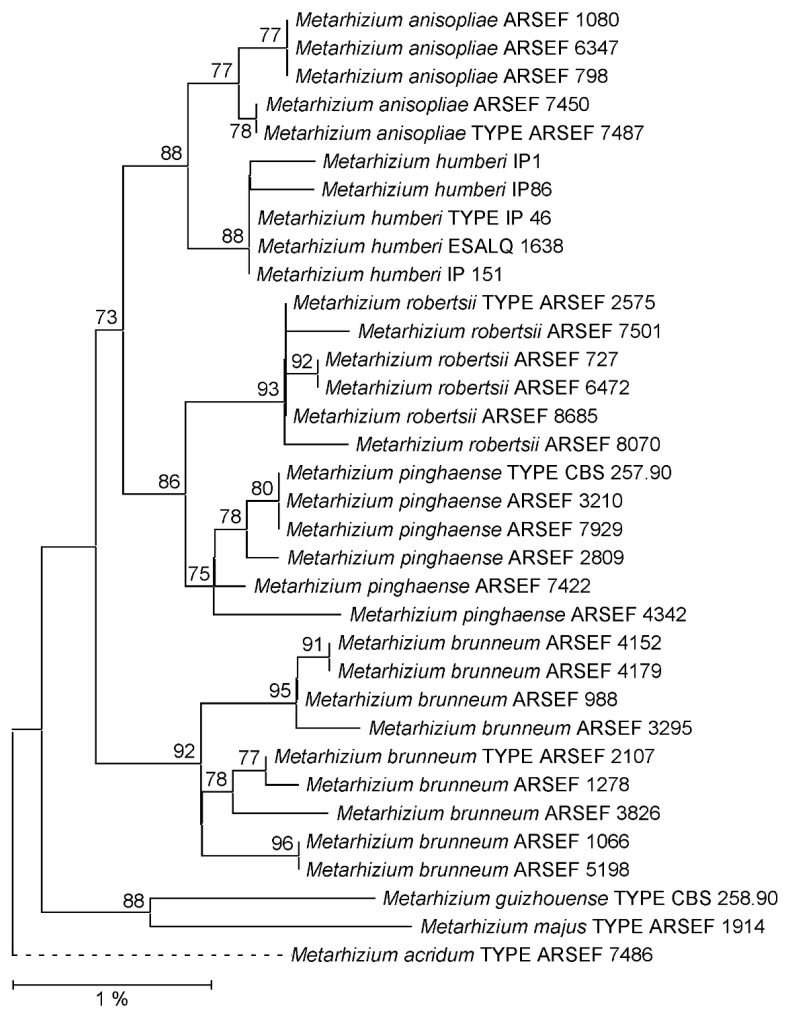
Maximum Likelihood (ML) phylogeny of *Metarhizium *fungi as reconstructed from 5TEF sequences. Terminal branches are labeled by genus, species and strain designations; “TYPE” denotes the nomenclatural type strain of a species. Numbers on branches indicate bootstrap support percentages. The size bar corresponds to 1% sequence divergence; branches drawn as dashed lines are not to scale. The orthologous sequence from the *M. acridum* type strain has been used as the outgroup.

**Figure 2:**
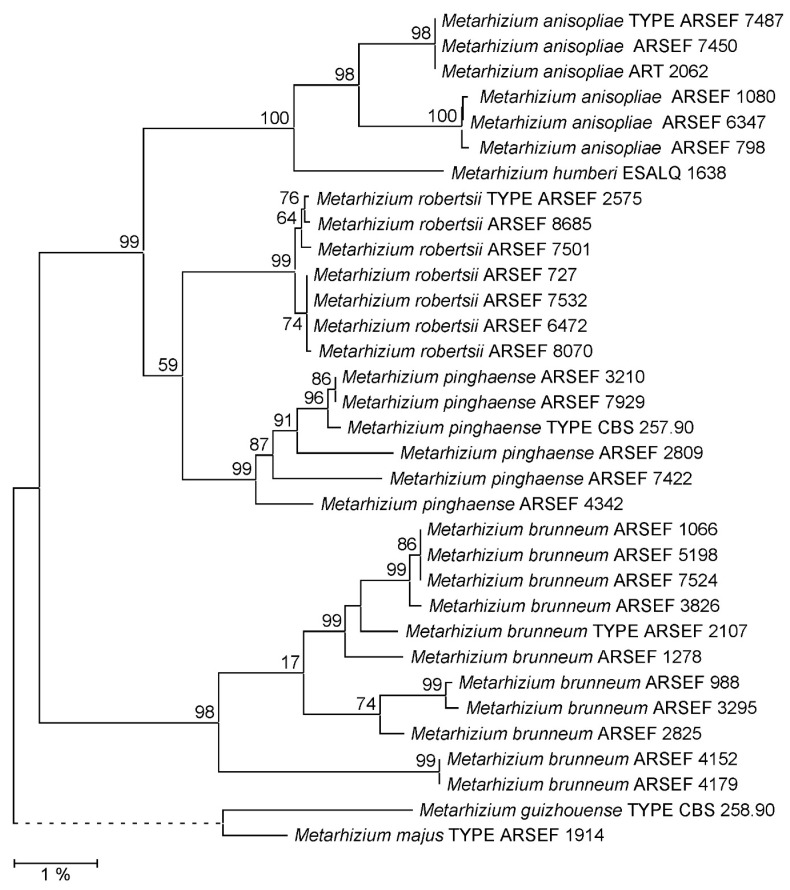
Maximum Likelihood (ML) phylogeny of *Metarhizium *fungi as reconstructed from rIGS sequences. Terminal branches are labeled by genus, species and strain designations; “TYPE” denotes the nomenclatural type strain of a species. Numbers on branches indicate bootstrap support percentages. The size bar corresponds to 1% sequence divergence; branches drawn as dashed lines are not to scale. The rIGS sequences from the *M. majus *and *M. guizhouense* type strains have been jointly used as the outgroup.

As far as not available from the GenBank database, EF1A, RPB1, RPB2 and 5TEF markers as well as complete rIGS sequences were determined for the *Metarhizium* strains comprised in the reference data set (Supplementary Table S1). Phylogenetic reconstruction using both the NJ and ML approaches confirmed that the PARB species were not consistently resolved by a concatenation of the EF1A, RPB1 and RPB2 marker sequences (Supplementary Figures S2 and S3). Using both 5TEF and rIGS as single taxonomic markers, PARB species clades were consistently resolved. However, when referring to the respective ML phylogenies (Figure 1 and Figure 2), bootstrap support values for the PARB species clades ranged from only 75% for *M. pinghaense* and 77% for *M. anisopliae* to 93% for *M. robertsii* in the 5TEF tree, whereas statistically sound species delineation with bootstrap support values ranging between 98% and 100% for all species clades was achieved using the rIGS marker. The substantially same difference in resolving power was reproduced in the NJ phylogenies reconstructed from 5TEF (60–97%) and rIGS (98–100%) sequence alignments (Supplementary Figures S4 and S5). Displaying a resolving power at least not inferior to that of the recognized 5TEF marker, the rIGS sequence was chosen as the candidate target locus for the development of both sequencing-independent and complementary-sequencing-dependent single-marker approaches to species-level identification within the *Metarhizium* PARB clade.

### 3.3. Diagnostic PCR Primer Design and Proof of Principle

**Figure 3:**
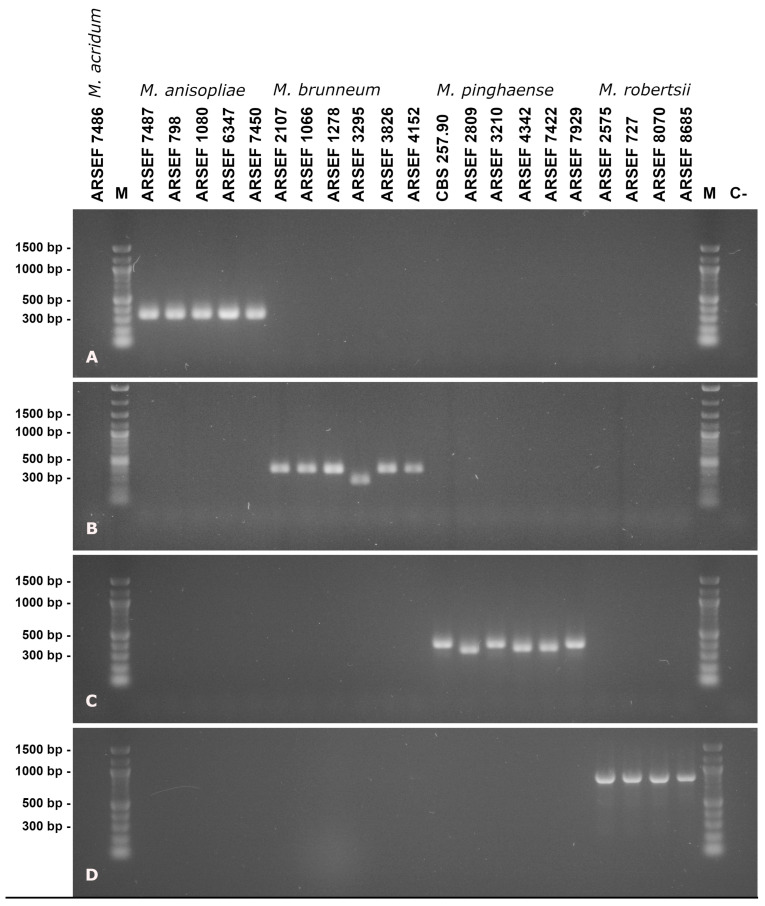
Agarose gel electrophoresis of diagnostic PCR products from template DNA of the reference strain set using PARB-species-discriminating primer pairs mani-IDF1/R1 (**A**), mbru-IDF1/R1 (**B**), mpin-IDF1/R1 (**C**) and mrob-IDF1/R1 (**D**). The length of relevant signals in the size standard is indicated in the left margin. Lane labels on top of the picture designate the *Metarhizium* species, strain or isolate; “**M**” denotes the size standard and “**C-**” denotes the negative (no template) control.

In order to design oligonucleotide primers for species-discriminating diagnostic PCR, rIGS sequences of the selected *M. anisopliae*, *M. brunneum*, *M. robertsii* and *M. pinghaense* reference strains (Supplementary Table S1) were carefully aligned (Supplementary Figure S6). Species-specific sequence features such as InDels or SNPs were identified, and pairs of oligonucleotide primers of varying length comprising these specific sequence features were designed and validated under fixed PCR parameters for functionality and discriminative power. Following this rationale gave rise to the definition of four diagnostic PCR primer pairs for the species *M. pinghaense*, *M. anisopliae*, *M. robertsii* and* M. brunneum *as listed in Table 1. When employed with the diagnostic PCR protocol, these primer pairs unambiguously discriminated at the species level between the* Metarhizium* PARB clade reference strains amplifying partial rIGS sequences exclusively from cognate genomic DNA templates (Figure 3).

### 3.4. Validation of the Species-Discriminating Diagnostic PCR

**Figure 4:**
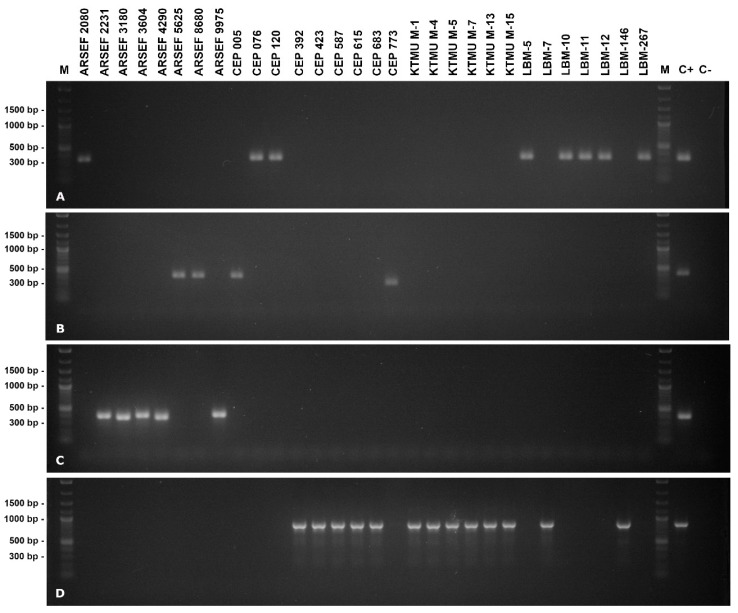
Agarose gel electrophoresis of diagnostic PCR products from template DNA of the validation strain set using PARB-species-discriminating primer pairs mani-IDF1/R1 (**A**), mbru-IDF1/R1 (**B**), mpin-IDF1/R1 (**C**) and mrob-IDF1/R1 (**D**). The length of relevant signals in the size standard is indicated in the left margin. Lane labels on top of the picture indicate the *Metarhizium* strain or isolate; “**M**” denotes the size standard, “**C-**” denotes the negative (no template) control and “**C+**” denotes the respective cognate type strain as reaction-specific positive control.

The functionality and species-discriminative power of the designed diagnostic primers were validated with *Metarhizium* fungi with no previous availability of rIGS sequence information that had not been used in method development. Genomic DNA was extracted from strains and isolates obtained from the ARSEF, CEPAVE, INISAV and KTMU culture collections that, according to a previous identification, were presumed to be assignable to the PARB clade or at least to the *M. anisopliae* species complex. These strains and isolates gave rise to the validation data set (Supplementary Table S2). When probed in separate reactions with the four diagnostic primer pairs, a single reaction was found positive with all strains and isolates tested (Figure 4), motivating assignment to exactly one of the four PARB species. From a total of 30 strains and isolates tested, 5 were thus found assignable to *M. pinghaense*, 8 to* M. anisopliae*, 13 to* M. robertsii* and 4 to *M. brunneum*. Respective previous assignments were thereby confirmed for 12 and rendered more precisely for 18 strains or isolates; in no case was a previous assignment contradicted.

### 3.5. Validation of a Multiplexed Diagnostic PCR Approach for Species Discrimination

In addition to the diagnostic PCR with one pair of species-discriminating primers, DNA samples from both the reference and validation strain sets were probed in a multiplexed diagnostic PCR approach, i.e., with all four diagnostic primer pairs being employed simultaneously in a single reaction. The outcome of these multiplexed PCR reactions (Supplementary Figure S7) was broadly in line with expectations from single primer pair reactions. In most reactions, a single main product was produced. For *M. anisopliae* and *M. robertsii* strains, the products generated were apparently identical in length to the respective type strain control, whereas there were important differences in the apparent size of PCR products generated from *M. brunneum* and *M. pinghaense* DNA samples.

### 3.6. The rIGS-ID800 Sequence as Molecular Taxonomic Marker for Species-Level Identification within the Metarhizium PARB Clade

**Figure 5:**
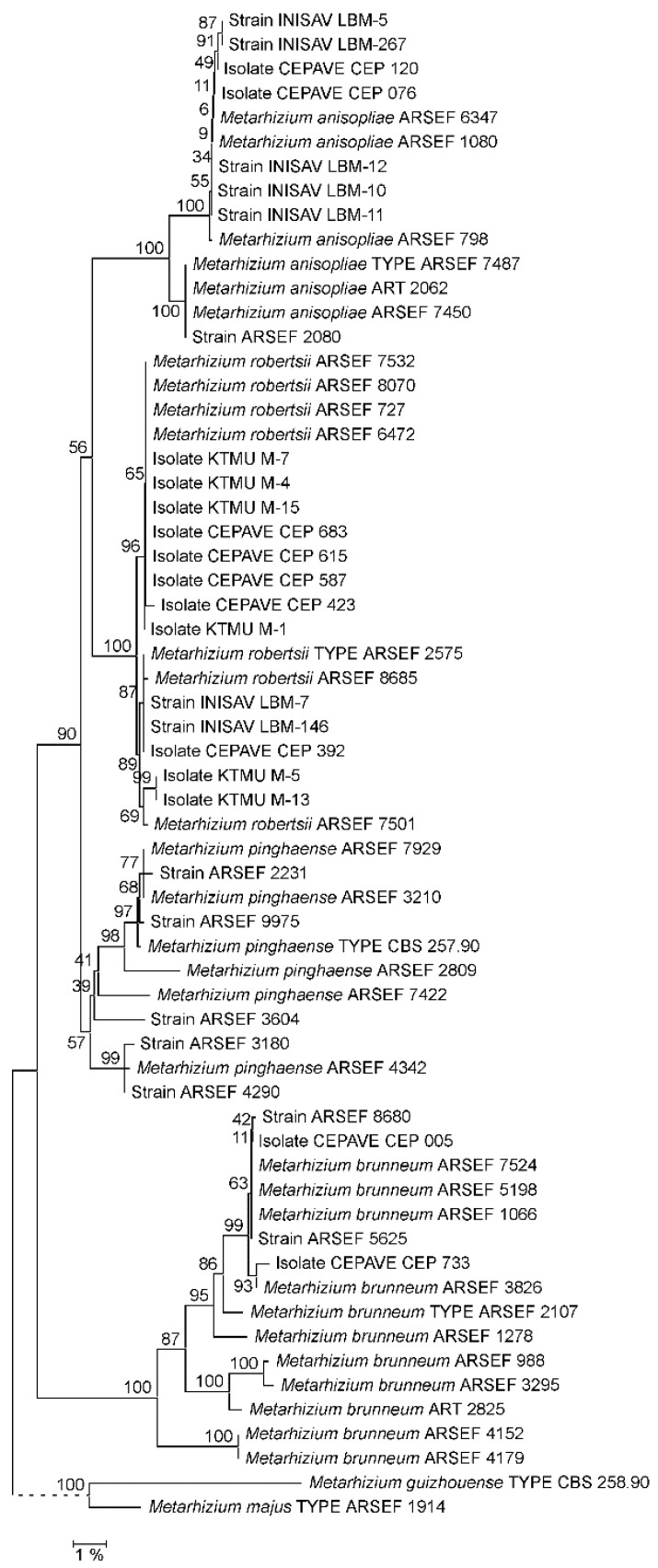
Neighbor Joining (NJ) phylogeny of *Metarhizium *fungi as reconstructed from rIGS-ID800 sequences. Terminal branches are labeled by genus, species and strain designations; “TYPE” denotes the nomenclatural type strain of a species. Numbers on branches indicate bootstrap support percentages. The size bar corresponds to 1% sequence divergence; branches drawn as dashed lines are not to scale. The rIGS sequences from the *M. majus and M. guizhouense* type strains have been jointly used as outgroup.

In order to assess the practicability of the use of the rIGS sequence as a molecular taxonomic marker, an approximately 700–800 bp portion of the spacer immediately downstream of the 28S rRNA encoding gene was PCR-amplified using primer pair Migs1-F1/Migs850-R1 and sequenced with primer Migs1-F2 (Supplementary Table S3). A single PCR product was generated from all *Metarhizium* strains comprised in the validation data set (Supplementary Figure S8). In the NJ tree reconstructed from a comparison of these sequences with those from the reference data set, the *M. anisopliae*, *M. brunneum* and *M. robertsii* clades received 100% bootstrap support, whereas support for the supposed *M. pinghaense* clade was only 57% (Figure 5). A qualitatively similar picture arose in the NJ phylogeny reconstructed from 5TEF marker sequences from the same set of strains and isolates, with bootstrap support values of 95–96% for the *M. anisopliae*, *M. brunneum* and *M. robertsii* clades and only 61% for the *M. pinghaense* clade (Supplementary Figure S9). Under the *caveat* of the generally low support for a monophyletic *M. pinghaense* clade, all fungi from the validation strain set were located in both the rIGS-ID800 and 5TEF trees in congruence with the respective diagnostic-PCR-based species-level assignment.

## 4. Discussion

The taxonomic species *M. pinghaense*, *M. anisopliae*, *M. robertsii *and *M. brunneum *form a tight clade within the *M. anisopliae* species complex. As these PARB species comprise numerous *Metarhizium *strains of high relevance for fundamental research in both entomopathogenicity and microbial control of insect pests, the fast and reliable species-level assignment of fungal isolates is highly solicited. Morphological and microscopic identification has clear limits with respect to the discrimination of PARB clade species. As for laboratories in many parts of the world where DNA sequencing is still not readily available, a sequencing-independent molecular approach can be expected to meet the needs of numerous researchers in the field. When analyzing the current 5TEF sequence entries in the Genbank database, 66/552 *Metarhizium* strains assigned to the PARB clade appeared mis-identified at the species level, and 13/552 were not assigned to a species.

Using the intergenic spacer (rIGS) sequence of the ribosomal RNA operon clusters of the *Metarhizium* genome as a target sequence, four pairs of PCR primers were demonstrated to discriminate between the four PARB species when analyzing *Metarhizium* strains and isolates from a wide range of geographic origins and isolation sources. Without exception, diagnostic-PCR-based species-level assignments were in line with 5TEF-sequence-based identifications.

In addition to species-specific diagnostic PCR employing a single out of the four species-discriminating primer pairs, the latter were simultaneously evaluated in a multiplexed diagnostic PCR assay, with the technically good result that a product of apparently similar size is generally generated from the same template DNA in the single primer pair and in the multiplexed reaction. However, it has to be stressed here that the present study did not aim to design diagnostic primers for use in a multiplexed assay based on the rIGS sequence as a diagnostic target. In this respect, our approach is complementary to the one presented by Mayerhofer et al. [37] who described PARB species discrimination in a multiplexed assay using ribosomal intergenic spacer (rIGS) sequences for *M. anisopliae *and* M. robertsii* and IGS sequences from protein-encoding genes (protIGS) for *M. brunneum* and *M. pinghaense*. Accordingly, the rationale followed in this study led to the design of primer pairs for the identification of *M. anisopliae *and* M. robertsii *that target rIGS sequence features and amplicons entirely different from those targeted by the rIGS-based multiplex primer pairs for these two species designed by Mayerhofer et al. [37].

Diagnostic primers to be employed in a multiplexed or competitive PCR assay have to respond to more restrictive criteria: as species-discrimination is mostly achieved due to the combined qualities of both primers of the cognate pair, amplicons targeted by different competing diagnostic primer pairs should not overlap in the target sequence, to prevent the formation of PCR products from hybrid primer pairs interfering with the formation of the solicited PCR product.

Moreover, in the multiplexed assay, species identification absolutely relies on the apparent size of the generated PCR product, i.e., there should be no insertions or deletions in the species-specific amplicons across the respective species. However, when basing molecular diagnostics upon intergenic spacer sequences that frequently carry InDels, this condition might be difficult to meet. In the present context, this problem appears particularly relevant for the identification of *M. brunneum* and *M. pinghaense*, as is obvious from the considerable PCR product size variation in the diagnostic PCR reactions targeting these species (Figure 3B,C and Figure 4B,C). Consistently, Mayerhofer et al. [37] stated that “no unique distinguishing sites for either *M. brunneum* or *M. pinghaense *were detected at rIGS”, and therefore employed primers targeting protein-encoding gene IGS without InDels to identify these species in a multiplexed assay. However, as rRNA operons and their rIGS are thought to be present in a number approximately two orders of magnitude higher across the fungal genome than specific protein-encoding genes and their protIGS, the combined use of rIGS and protIGS for PCR diagnostics might potentially complicate the further development of quantifying diagnostics such as real-time PCR.

The authors of this study share the expectation expressed by Kepler and Rehner [27] that species diversity in the *M. anisopliae* complex is currently under-represented. However, with the description of new species within the complex and presumably within the PARB clade, too, restrictions on primer design for multiplexed species-discriminating PCR approaches will likely increase, affecting the feasibility of multiplexed diagnostics spanning an extended or enriched taxonomic range. Restrictions will presumably be less severe for approaches targeting a single species, which is the approach favored here. It goes without saying that both approaches can potentially complement each other in a pragmatic manner. When reducing the level of assayed taxonomic complexity of the PARB clade to the four *Metarhizium* species that gave rise to its designation, the multiplexed diagnostic PCR protocol developed here (Supplementary Figure S7) might serve as a first analysis step intended, for instance, to select a sub-set of fungal isolates for downstream identification.

Complementary to these sequencing-independent approaches, the suitability of the rIGS sequence as a marker for single sequence molecular taxonomic studies was assessed. A previous evaluation of the rIGS marker in multi-sequence phylogenetic studies of *Metarhizium* fungi [38] has been hampered by the fact that the complete intergenic spacer sequence is long (2–3 kb) and that due to the presence of repetitive elements and a tendency to secondary structure formation amplification, sequencing may be difficult. Moreover, due to the high degree of rIGS sequence variation, the definition of universal internal primers for the amplification of partial sequences is difficult.

In the present study, the internal primer Migs850-R1 binding to a sequence motif conserved across the PARB clade species was designed and used together with universal external primers targeting the 3′-end of the 28S rRNA gene to reliably amplify and sequence an approximately 800 bp long part of rIGS, termed rIGS-ID800, immediately downstream of the 28S rRNA gene. Using rIGS-ID800 as a single sequence marker for molecular taxonomic analysis of the *Metarhizium* strains under study gave rise to unequivocal discrimination of the PARB clade species *M. anisopliae*, *M. brunneum* and *M. robertsii* with higher statistical support than in the corresponding 5TEF-based phylogeny. In contrast, strains of the species *M. pinghaense* did not cluster in a well-supported clade in phylogenies reconstructed from both the rIGS-ID800 and 5TEF sequences, indicating that, in its current extension, this species is most likely polyphyletic.

## 5. Conclusions

The present study demonstrated that reliable species discrimination within the *Metarhizium* PARB clade is possible by both sequencing-independent diagnostic PCR and sequencing-dependent single marker comparison based on the partial ribosomal intergenic spacer sequence termed rIGS-ID800. A PCR tool for sequencing-independent species assignment is thereby backed up by a correlated ID sequence definition suitable for, e.g., new isolate descriptions requiring molecular data.

## Figures and Tables

**Table 1:** PARB-species-discriminating diagnostic PCR primer pairs used in this study.

Primer Designation	Nucleotide Sequence (5′ => 3′)	Expected Specificity	Product Size (bp)
mani-IDF1	GGCTATAGTYAACTTTTGGACTTGC	* M. anisopliae *	335
mani-IDR1	ACAAAAAAATCAACTCACGCCTATAT		
mbru-IDF1	TGACTTKTGGACWYGGCGGA	* M. brunneum *	277–405
mbru-IDR1	CGCTACYRGGCTCTCGTGGT		
mpin-IDF1	GTGCCGGGGCCCTGTAG	* M. pinghaense *	337–391
mpin-IDR1	GCCAAAATACTAGGAACTTGTATA		
mrob-IDF1	GCGGGTGTTGGGGTTAAT	* M. robertsii *	841–842
mrob-IDR1	CTAAAAGTATTGGCTGCGGC		

## Data Availability

Sequence data analyzed in this study are publicly available from the Genbank database https://www.ncbi.nlm.nih.gov (accessed on 7 September 2023) under nucleotide sequence accession numbers listed in Supplementary Tables S1 and S2 to this study.

## Supplementary Materials

The following supporting information can be downloaded at https://www.mdpi.com/article/10.3390/jof9100996/s1: Supplementary Figure S1: Neighbor-Joining (NJ) phylogeny of *Metarhizium *PARB clade fungi as reconstructed from 5TEF sequences. Supplementary Figure S2: Maximum Likelihood (ML) phylogeny of *Metarhizium *fungi as reconstructed from a concatenation of EF1A-RPB1-RPB2 sequences. Supplementary Figure S3: Neighbor-Joining (NJ) phylogeny of *Metarhizium *fungi as reconstructed from a concatenation of EF1A-RPB1-RPB2 sequences. Supplementary Figure S4: Neighbor-Joining (NJ) phylogeny of *Metarhizium *fungi as reconstructed from 5TEF sequences. Supplementary Figure S5: Neighbor-Joining (NJ) phylogeny of *Metarhizium *fungi as reconstructed from rIGS sequences. Supplementary Figure S6: Alignment of ribosomal intergenic spacer (rIGS) sequences used for diagnostic primer design. Supplementary Figure S7: Agarose gel electrophoresis of multiplexed diagnostic PCR reactions. Supplementary Figure S8: Agarose gel electrophoresis of PCR reactions from template DNA of the validation strain set amplifying the rIGS-ID800 sequence with primer pair Migs1-F1/Migs850-R1. Supplementary Figure S9: Neighbor-Joining (NJ) phylogeny of *Metarhizium *fungi as reconstructed from 5TEF sequences. Supplementary Table S1: Reference data set of *Metarhizium* strains and marker sequences. Supplementary Table S2: Validation data set of *Metarhizium* strains and marker sequences. Supplementary Table S3: Preparative PCR primers used and reaction-specific parameters applied in this study. Refs. [48,49,50,51,52,53,54] are cited in the Supplementary Tables S1–S3.
