# Supplementary material for: Species Discrimination within the Metarhizium PARB Clade: Ribosomal Intergenic Spacer (rIGS)-Based Diagnostic PCR and Single Marker Taxonomy
Source: J Fungi (Basel). 2023 Oct 8;9(10):996. doi: 10.3390/jof9100996 (PMC10607842; doi:10.3390/jof9100996)
Supplement: Supplementary file 1 [file jof-09-00996-s001.zip › Suppl Figure S6.pdf]

```
mani-IDF1                               ---------- ---------- ---------- ---------- ---------- ---------- ---------- ---------- ---------- ----------   100
mani-IDR1                               ---------- ---------- ---------- ---------- ---------- ---------- ---------- ---------- ---------- ----------   100
Metarhizium_anisopliae_TYPE_ARSEF_7487  GCGGGGCGGG TGTTGGGGTT AAAGCCACCA A-GTA-GTGG TTACAGGCTT GAGGGGAG-- CTGCCGAG-- -TGGT-GGTT CTAGAGGGAA AAGTCTGCCA   100
Metarhizium_anisopliae_ARSEF_1080       GCGGGGCGGG TGTTGGGGTT AGAGCCACCA A-GTA-GTGG TTACAGGCTT GAGGAGAG-- CTGCCGAG-- -TGGT-GGTT CTAGAGGGAA AAGTCTGCCA   100
Metarhizium_anisopliae_ARSEF_6347       GCGGGGCGGG TGTTGGGGTT AGAGCCACCA A-GTA-GTGG TTACAGGCTT GAGGAGAG-- CTGCCGAG-- -TGGT-GGTT CTAGAGGGAA AAGTCTGCCA   100
Metarhizium_anisopliae_ARSEF_7450       GCGGGGCGGG TGTTGGGGTT AAAGCCACCA A-GTA-GTGG TTACAGGCTT GAGGGGAG-- CTGCCGAG-- -TGGT-GGTT CTAGAGGGAA AAGTCTGCCA   100
Metarhizium_anisopliae_ARSEF_798        GCGGGGCGGG TGTTGGGGTT AGAGCCACCA A-GTA-GTGG TTACAGGCTT GAGGAGAG-- CTGCCGAG-- -TGGT-GGTT CTAGAGGGAA AAGTCTGCCA   100
Metarhizium_anisopliae_ART_2062         GCGGGGCGGG TGTTGGGGTT AAAGCCACCA A-GTA-GTGG TTACAGGCTT GAGGGGAG-- CTGCCGAG-- -TGGT-GGTT CTAGAGGGAA AAGTCTGCCA   100

mbru-IDF1                               ---------- ---------- ---------- ---------- ---------- ---------- ---------- ---------- ---------- ----------   100
mbru-IDR1                               ---------- ---------- ---------- ---------- ---------- ---------- ---------- ---------- ---------- ----------   100
Metarhizium_brunneum_TYPE_ARSEF_2107    GCGGGGCGGG TGTTGGGGTT AGAGCCACCG A-GTA-GTGG CCGCAGGCTG GAGGAGAG-- CCGCCGAG-- -TGGT-GGTT C-AGGAGGGA AAATCTGCCA   100
Metarhizium_brunneum_ARSEF_1066         GCGGGGCGGG TGTTGGGGTT AGAGCCACCG A-GTA-GTGG CCGCAGGCTT GAGGAGAG-- CCGCCGAG-- -TGGT-GGTT C-AGGAGGGA AAATCTGCCA   100
Metarhizium_brunneum_ARSEF_1278         GCGGGGCGGG TGTTGGGGTT AGAGCCACCG A-GTA-GTGG CCGCAGGCTT GAGGAGAG-- CCGCCGAG-- -TGGT-GGTT C-AGGAGGGA AAATCTGCCA   100
Metarhizium_brunneum_ARSEF_3295         GCGGGGCGGG TGTTGGGGTT AGAGCCACCG A-GTA-GTGG CCGCAGGCTT GAGGAGAG-- CCGCCGAG-- -TGGT-GGTT C-GGGAGGGA AAATCTGCCA   100
Metarhizium_brunneum_ARSEF_3826         GCGGGGCGGG TGTTGGGGTT AGAGCCACCG A-GCA-GTGG CCGCAGGCTT GAGGAGAG-- CCGCCGAG-- -TGGT-GGTT C-AGGAGGGA AAATCTGCCA   100
Metarhizium_brunneum_ARSEF_4152         GCGGGGCGGG TGTTGGGGTT AGAGCCACCG A-GTA-GTGG CCGCAGGCTT GAGGAGAG-- CCGCCGAG-- -TGGT-GGTT C-GGGAGGGA AAATCTGCCA   100
Metarhizium_brunneum_ARSEF_4179         GCGGGGCGGG TGTTGGGGTT AGAGCCACCG A-GTA-GTGG CCGCAGGCTT GAGGAGAG-- CCGCCGAG-- -TGGT-GGTT C-GGGAGGGA AAATCTGCCA   100
Metarhizium_brunneum_ARSEF_5198         GCGGGGCGGG TGTTGGGGTT AGAGCCACCG A-GTA-GTGG CCGCAGGCTT GAGGAGAG-- CCGCCGAG-- -TGGT-GGTT C-AGGAGGGA AAATCTGCCA   100
Metarhizium_brunneum_ARSEF_7524         GCGGGGCGGG TGTTGGGGTT AGAGCCACCG A-GTA-GTGG CCGCAGGCTT GAGGAGAG-- CCGCCGAG-- -TGGT-GGTT C-AGGAGGGA AAATCTGCCA   100
Metarhizium_brunneum_ARSEF_988          GCGGGGCGGG TGTTGGGGTC AGAGCCACCG A-GTA-GTGG CCGCAGGCTT GAGGAGAG-- CCGCCGAG-- -TGGT-GGTT C-GGGAGGGA AAATCTGCCA   100
Metarhizium_brunneum_ART_2825           GCGGGGCGGG TGTTGGGGTT AGAGCCACCG A-GTA-GTGG CCGCAGGCTT GAGGAGAG-- CCGCCGAG-- -TGGT-GGTT C-GGGGGGGG AAATCTGCCA   100

mpin-IDF1                               ---------- ---------- ---------- ---------- ---------- ---------- ---------- ---------- ---------- ----------   100
mpin-IDR1                               ---------- ---------- ---------- ---------- ---------- ---------- ---------- ---------- ---------- ----------   100
Metarhizium_pingshaense_TYPE_CBS_257.90 GCGGGGCGGG TGTTGGGGTT AAAGCCACCA AAGTA-GTGG TTGCAGGCTT GAGGAGAG-- CCGCCGAGCG GTGGTTGGTT CTAGAGGGGA AAATCTGCCA   100
Metarhizium_pingshaense_ARSEF_2809      GCGGGGCGGG TGTTGGGGTT AAAGCCACCG A-GTA-GTGG TTGCAGGCTT GAGGAGAGAG CTGCCGAGCG GT----GGTT CTAGAGGGGA AAATCTGCCA   100
Metarhizium_pingshaense_ARSEF_3210      GCGGGGCGGG TGTTGGGGTT AAAGCCGCCA AAGTA-GTGG TTGCAGGCTT GAGGAGAG-- CCGCCGAGCG GTGGTTGGTT CTAGAGGGGA AAATCTGCCA   100
Metarhizium_pingshaense_ARSEF_4342      GCGGGGCGGG TGTTGGGGTT AAAGCCACCA A-GTA-GTGG TTGCAGGCTT GAGGAGAG-- CCGCCGAG-- -TGGT-GGTT CTAGAGGGAA AAATCTGCCA   100
Metarhizium_pingshaense_ARSEF_7422      GCGGGGCGGG TGTTGGGGTT AAAGCCACCA A-GTAAGTGG CTGCAGGCTT GAGGAGAG-- CCGCCGGGCG G----TGGTT CTAGAGGGGA AAATCTGCCA   100
Metarhizium_pingshaense_ARSEF_7929      GCGGGGCGGG TGTTGGGGTT AAAGCCGCCA AAGTA-GTGG TTGCAGGCTT GAGGAGAG-- CCGCCGAGCG GTGGTTGGTT CTAGAGGGGA AAATCTGCCA   100

mrob-IDF1                               -----GCGGG TGTTGGGGTT AAT------- ---------- ---------- ---------- ---------- ---------- ---------- ----------   100
mrob-IDR1                               ---------- ---------- ---------- ---------- ---------- ---------- ---------- ---------- ---------- ----------   100
Metarhizium_robertsii_TYPE_ARSEF_2575   GCGGGGCGGG TGTTGGGGTT AATGCCACCA A-GTA-GTGG TTGCAGGCTT GAGGAGAG-- CCGCCGAG-- -TGGT-GGTT CTAGAGGGAA AAATCTGCCA   100
Metarhizium_robertsii_ARSEF_6472        GCGGGGCGGG TGTTGGGGTT AATGCCACCA A-GTA-GTGG TTGCAGGCTT GAGGAGAG-- CCGCCGAG-- -TGGT-GGTT CTAGAGGGAA AAATCTGCCA   100
Metarhizium_robertsii_ARSEF_727         GCGGGGCGGG TGTTGGGGTT AATGCCACCA A-GTA-GTGG TTGCAGGCTT GAGGAGAG-- CCGCCGAG-- -TGGT-GGTT CTAGAGGGAA AAATCTGCCA   100
Metarhizium_robertsii_ARSEF_7501        GCGGGGCGGG TGTTGGGGTT AATGCCACCA A-GTA-GTGG TTGCAGGCTT GAGGAGAG-- CCGCCGAG-- -TGGT-GGTT CTAGAGGGAA AAATCTGCCA   100
Metarhizium_robertsii_ARSEF_7532        GCGGGGCGGG TGTTGGGGTT AATGCCACCA A-GTA-GTGG TTGCAGGCTT GAGGAGAG-- CCGCCGAG-- -TGGT-GGTT CTAGAGGGAA AAATCTGCCA   100
Metarhizium_robertsii_ARSEF_8070        GCGGGGCGGG TGTTGGGGTT AATGCCACCA A-GTA-GTGG TTGCAGGCTT GAGGAGAG-- CCGCCGAG-- -TGGT-GGTT CTAGAGGGAA AAATCTGCCA   100
Metarhizium_robertsii_ARSEF_8685        GCGGGGCTGG TGTTGGGGTT AATGCCACCA A-GTA-GTGG TTGCAGGCTT GAGGAGAG-- CCGCCGAG-- -TGGT-GGTT CTAGAGGGAA AAATCTGCCA   100
```

```
mani-IDF1                               ---------- ---------- ---------- ---------- ---------- ---------- ---------- ---------- ---------- ----------   200
mani-IDR1                               ---------- ---------- ---------- ---------- ---------- ---------- ---------- ---------- ---------- ----------   200
Metarhizium_anisopliae_TYPE_ARSEF_7487  AGTTC--AAA GGTCCAGGCA AGCGAAAAG- TTTACCAAGT CGGAAGGTTG GGCGAGTAAA AAAAT-TACC AAGTCCAAAA TATTGGCGGT AACCTACCCG   200
Metarhizium_anisopliae_ARSEF_1080       GGTTC--AAA GGTCCAGGCA AGCGAAAAG- TTTACCAAGT CGGAAGGTTG GGCGAGTAAA AAAAT-TACC AAGTCCAAAA TATTGGCGGT AACCTACCCG   200
Metarhizium_anisopliae_ARSEF_6347       GGTTC--AAA GGTCCAGGCA AGCGAAAAG- TTTACCAAGT CGGAAGGTTG GGCGAGTAAA AAAAT-TACC AAGTCCAAAA TATTGGCGGT AACCTACCCG   200
Metarhizium_anisopliae_ARSEF_7450       AGTTC--AAA GGTCCAGGCA AGCGAAAAG- TTTACCAAGT CGGAAGGTTG GGCGAGTAAA AAAAT-TACC AAGTCCAAAA TATTGGCGGT AACCTACCCG   200
Metarhizium_anisopliae_ARSEF_798        GGTTC--AAA GGTCCAGGCA AGCGAAAAG- TTTACCAAGT CGGAAGGTTG GGCGAGTAAA AAAAT-TACC AAGTCCAAAA TATTGGCGGT AACCTACCCG   200
Metarhizium_anisopliae_ART_2062         AGTTC--AAA GGTCCAGGCA AGCGAAAAG- TTTACCAAGT CGGAAGGTTG GGCGAGTAAA AAAAT-TACC AAGTCCAAAA TATTGGCGGT AACCTACCCG   200

mbru-IDF1                               ---------- ---------- ---------- ---------- ---------- ---------- ---------- ---------- ---------- ----------   200
mbru-IDR1                               ---------- ---------- ---------- ---------- ---------- ---------- ---------- ---------- ---------- ----------   200
Metarhizium_brunneum_TYPE_ARSEF_2107    AGCTC--AAA GGTC-AGGCA AGCGAGAGG- TTTACCGAGT CGGAAAGTCG GGCGAGTAAA AAGATATACC AAGTCCAAAA TACTGCCGGC AACATACCCG   200
Metarhizium_brunneum_ARSEF_1066         AGCTCTCAAA GGTC-AGGCA AGCGAGAGG- TTTACCGAGT CGGAAAGTCG GGCGAGTAAA AAGATATACC AAGTCCAAAA TACTGCCGGC AACATACCCG   200
Metarhizium_brunneum_ARSEF_1278         AGCTC--AAA GGTC-AGGCA AGCGAGAGGT TTTACCGAGT CGGAAAGTCG GGCGAGTAAA AAGATATACC AAGTCCAAAA TACTGCCGGC AACATACCCG   200
Metarhizium_brunneum_ARSEF_3295         AGCTC--AAA GGTC-AGGCA AGCGAGAGG- TTTACCGGGT CGGAAAGTCG GGCGAGTAAA AAGATATACC AAGTCCAAAA CACTGCCGGC AACATACCCG   200
Metarhizium_brunneum_ARSEF_3826         AGCTC--AAA GGTC-AGGCA AGCGAGAGG- TTTACCGAGT CGGAAAGTCG GGCGAGTAAA AAGATATACC AAGTCCAAAA TACTGCCGGC AACATACCCG   200
Metarhizium_brunneum_ARSEF_4152         AGCTC--AAA GGTC-AGGCA AGCGAGAGG- TTTACCGGGT CGGAAAGTCG GGCGAGTAAA AAGATATACC AAGTCCAAAA TACTGCCGGC AACATACCCG   200
Metarhizium_brunneum_ARSEF_4179         AGCTC--AAA GGTC-AGGCA AGCGAGAGG- TTTACCGGGT CGGAAAGTCG GGCGAGTAAA AAGATATACC AAGTCCAAAA TACTGCCGGC AACATACCCG   200
Metarhizium_brunneum_ARSEF_5198         AGCTCTCAAA GGTC-AGGCA AGCGAGAGG- TTTACCGAGT CGGAAAGTCG GGCGAGTAAA AAGATATACC AAGTCCAAAA TACTGCCGGC AACATACCCG   200
Metarhizium_brunneum_ARSEF_7524         AGCTCTCAAA GGTC-AGGCA AGCGAGAGG- TTTACCGAGT CGGAAAGTCG GGCGAGTAAA AAGATATACC AAGTCCAAAA TACTGCCGGC AACATACCCG   200
Metarhizium_brunneum_ARSEF_988          AGCTC--AAA GGTC-AGGCA AGCGAGAGG- TTTACCGGGT CGGAAAGTCG GGCGAGTAAA AAGATATACC AAGTCCAAAA CACTGCCGGC AACATACCCG   200
Metarhizium_brunneum_ART_2825           AGCTC--AAA GGTC-AGGCA AGCGAGAGG- TTTACCGAGT CGGAAAGTCG GGCGAGTAAA AAGATATACC AAGTCCAAAA TACTGCCGGC AACATATCCG   200

mpin-IDF1                               ---------- ---------- ---------- ---------- ---------- ---------- ---------- ---------- ---------- ----------   200
mpin-IDR1                               ---------- ---------- ---------- ---------- ---------- ---------- ---------- ---------- ---------- ----------   200
Metarhizium_pingshaense_TYPE_CBS_257.90 AGTTC--AAA GGTC-AGGCA AGCGAAAGG- TTTACCGAGT CGGAAGGTTG GGCGAGTAAA AAATT-TACC AAGTCCAAAA TACTGCCGGC AACATACCCG   200
Metarhizium_pingshaense_ARSEF_2809      AGTTC--AAA GGTC-AGGCA AGCGAAAGGT TGTACCGAGT CGGAAGGTTG GGCGAGTAAA GAATT-TACC AAGTCCAAAA TACTGCCGGC AACATACCCG   200
Metarhizium_pingshaense_ARSEF_3210      AGTTC--AAA GGTC-AGGCA AGCGAAAGG- TTTACCGAGT CGGAAGGTTG GGCGAGTAAA AAATT-TACC AAGTCCAAAA TACTGCCGGC AACATACCCG   200
Metarhizium_pingshaense_ARSEF_4342      AGTCC--AAA GGTC-AGGCA AGCGAAAGG- TTCACCGAGT CGGAAAGTCG GGCGAGTAAA AAATT-TACC AAGTCCAAAA TACTGCCGGC AACATACCCG   200
Metarhizium_pingshaense_ARSEF_7422      AGTTC--AAA GGTC-AGGCA AGCGAAAGG- TTTACCGAGT CGGAAGGTTG GGCGAGTAAA AAATT-TACC AAGTCCAAAA TACTGCCGGC AACATACCCG   200
Metarhizium_pingshaense_ARSEF_7929      AGTTC--AAA GGTC-AGGCA AGCGAAAGG- TTTACCGAGT CGGAAGGTTG GGCGAGTAAA AAATT-TACC AAGTCCAAAA TACTGCCGGC AACATACCCG   200

mrob-IDF1                               ---------- ---------- ---------- ---------- ---------- ---------- ---------- ---------- ---------- ----------   200
mrob-IDR1                               ---------- ---------- ---------- ---------- ---------- ---------- ---------- ---------- ---------- ----------   200
Metarhizium_robertsii_TYPE_ARSEF_2575   AGTTC--AAA GGTC-AGGCA AGCGAAAGG- TTTACCGAGT CGGAAGGTTG GGCGAGTAAA AAAAT-TACC AAGTCCAAAA TACTGGCGGT AACCTACCCG   200
Metarhizium_robertsii_ARSEF_6472        AGTTC--AAA GGTC-AGGCA AGCGAAAGG- TTTACCGAGT CGGAAGGTTG GGCGAGTAAA AAAAT-TACC AAGTCCAAAA TACTGGCGGT AACCTACCCG   200
Metarhizium_robertsii_ARSEF_727         AGTTC--AAA GGTC-AGGCA AGCGAAAGG- TTTACCGAGT CGGAAGGTTG GGCGAGTAAA AAAAT-TACC AAGTCCAAAA TACTGGCGGT AACCTACCCG   200
Metarhizium_robertsii_ARSEF_7501        AGTTC--AAA GGTC-AGGCA AGCGAAAGG- TTTACCGAGT CGGAAGGTTG GGCGAGTAAA AAAAT-TACC AAGTCCAAAA TACTGGCGGT AACCTACCCG   200
Metarhizium_robertsii_ARSEF_7532        AGTTC--AAA GGTC-AGGCA AGCGAAAGG- TTTACCGAGT CGGAAGGTTG GGCGAGTAAA AAAAT-TACC AAGTCCAAAA TACTGGCGGT AACCTACCCG   200
Metarhizium_robertsii_ARSEF_8070        AGTTC--AAA GGTC-AGGCA AGCGAAAGG- TTTACCGAGT CGGAAGGTTG GGCGAGTAAA AAAAT-TACC AAGTCCAAAA TACTGGCGGT AACCTACCCG   200
Metarhizium_robertsii_ARSEF_8685        AGTTC--AAA GGTC-AGGCA AGCGAAAGG- TTTACCGAGT CGGAAGGTTG GGCGAGTAAA AAAAT-TACC AAGTCCAAAA TACTGGCGGT AACCTACCCG   200
```

```
mani-IDF1                                ---------- ---------- ---------- ---------- ---------- ---------- ---------- ---------- ---------- ----------   300
mani-IDR1                                ---------- ---------- ---------- ---------- ---------- ---------- ---------- ---------- ---------- ----------   300
Metarhizium_anisopliae_TYPE_ARSEF_7487   GGACTCCGAG TAAGTCGGGG AAAAAGTTGG CGGATTTTTT CCCAACCAAC TCAACTTTTG ACCAATTT-A CCTGCCCGAG CTCACGGGTA GGCTGCGGTT   300
Metarhizium_anisopliae_ARSEF_1080        GGACTCCGAG TAAGTCGGGG AAAAAGTTGG CGGATTTTTC CCCAACCAAC TCAACTTTTG ACCAATTT-A CCTGCCCGAG CTCACGGGTA GGCTGCGGTT   300
Metarhizium_anisopliae_ARSEF_6347        GGACTCCGAG TAAGTCGGGG AAAAAGTTGG CGGATTTTTC CCCAACCAAC TCAACTTTTG ACCAATTT-A CCTGCCCGAG CTCACGGGTA GGCTGCGGTT   300
Metarhizium_anisopliae_ARSEF_7450        GGACTCCGAG TAAGTCGGGG AAAAAGTTGG CGGATTTTTT CCCAACCAAC TCAACTTTTG ACCAATTT-A CCTGCCCGAG CTCACGGGTA GGCTGCGGTT   300
Metarhizium_anisopliae_ARSEF_798         GGACTCCGAG TAAGTCGGGG AAAAAGTTGG CGGATTTTTC CCCAACCAAC TCAACTTTTG ACCAATTT-A CCTGCCCGAG CTCACGGGTA GGCTGCGGTT   300
Metarhizium_anisopliae_ART_2062          GGACTCCGAG TAAGTCGGGG AAAAAGTTGG CGGATTTTTT CCCAACCAAC TCAACTTTTG ACCAATTT-A CCTGCCCGAG CTCACGGGTA GGCTGCGGTT   300

mbru-IDF1                                ---------- ---------- ---------- ---------- ---------- ---------- ---------- ---------- ---------- ----------   300
mbru-IDR1                                ---------- ---------- ---------- ---------- ---------- ---------- ---------- ---------- ---------- ----------   300
Metarhizium_brunneum_TYPE_ARSEF_2107     GGACCCCGAG TAAGTCGGGG AAGAAGTTGG CGGATGTTTT TCCAGCCAAC TCAACTTT-G ACCAATTT-A CCTGCCCGAG CTCACGGGTA GGCTGCGGTT   300
Metarhizium_brunneum_ARSEF_1066          GGACCCCGAG TAAGTCGGGG AAGAAGTTGG CGGGTGTTTT TCCAGCCAAC TCAACTTT-G ACCAATTTTA CCTGCCCGAG CTCACGGGTA GGCTGCGGTT   300
Metarhizium_brunneum_ARSEF_1278          GGACCCCGAG TAAGTCGGGG AAGAAGTTGG CGGGTGTTTT CCCAGCCAAC TCAACTCT-G ACCAATTT-A CCTGCCCGAG CTCACGGGTA GGCTGCGGTT   300
Metarhizium_brunneum_ARSEF_3295          GGACCCCGAG TAAGTCGGGG AAGAAGTTGG CGGATGTTTT TCCAGCCAAC TCAACTTT-G ACCAATTT-A CCTGCCCGAG CTCACGGGTA GGCTGCGGTT   300
Metarhizium_brunneum_ARSEF_3826          GGACCCCGAG TAAGTCGGGG AAGAAGTTGG CGGATGTTTT TCCAGCCAAC TCAACTTT-G ACCAATTT-A CCTGCCCGAG CTCACGGGTA GGCTGCGGTT   300
Metarhizium_brunneum_ARSEF_4152          GGACCCCGAG TAAGTCGGGG AAGAAGTTGG CGGATGTTTT CCCAGCCAAC TCAACTTT-G ACCAATTT-A CCTGCCCGAG CTCACGGGTA GGCTGCGGTT   300
Metarhizium_brunneum_ARSEF_4179          GGACCCCGAG TAAGTCGGGG AAGAAGTTGG CGGATGTTTT CCCAGCCAAC TCAACTTT-G ACCAATTT-A CCTGCCCGAG CTCACGGGTA GGCTGCGGTT   300
Metarhizium_brunneum_ARSEF_5198          GGACCCCGAG TAAGTCGGGG AAGAAGTTGG CGGGTGTTTT TCCAGCCAAC TCAACTTT-G ACCAATTTTA CCTGCCCGAG CTCACGGGTA GGCTGCGGTT   300
Metarhizium_brunneum_ARSEF_7524          GGACCCCGAG TAAGTCGGGG AAGAAGTTGG CGGGTGTTTT TCCAGCCAAC TCAACTTT-G ACCAATTTTA CCTGCCCGAG CTCACGGGTA GGCTGCGGTT   300
Metarhizium_brunneum_ARSEF_988           GGACCCCGAG TAAGTCGGGG AAGAAGTTGG CGGATGTTTT TCCAGCCAAC TCAACTTT-G ACCAATTT-A CCTGCCCGAG CTCACGGGTA GGCTGCGGTT   300
Metarhizium_brunneum_ART_2825            GGACCCCGAG TAAGTCGGGG AAGAAGTTGG CGGATGTTTT TCCAGCCAAC TCAACTTT-G ACCAATTT-A CCTGCCCGAG CTCACGGGTA GGCTGCGGTT   300

mpin-IDF1                                ---------- ---------- ---------- ---------- ---------- ---------- ---------- ---------- ---------- ----------   300
mpin-IDR1                                ---------- ---------- ---------- ---------- ---------- ---------- ---------- ---------- ---------- ----------   300
Metarhizium_pingshaense_TYPE_CBS_257.90  GGACTCCGAG TAAGTCGGGG AAGAAGTTGG CGGATTTTTT CCCAACCAAC TCAACTTTTG ACCAATTT-A CCTGCCCGAG CTCACGGGTA GGCTGCGGTT   300
Metarhizium_pingshaense_ARSEF_2809       GGACTCCGAG TAAGTCGGGG AAGAAGTTGG CGGATTTTTT CCCAACCAAC TCAACTTTTG ACCAATTT-A CCTGCCCGAG CTCACGGGTA GGCTGCGGTT   300
Metarhizium_pingshaense_ARSEF_3210       GGACTCCGAG TAAGTCGGGG AAGAAGTTGG CGGATTTTTT CCCAACCAAC TCAACTTTTG ACCAATTT-A CCTGCCCGAG CTCACGGGTA GGCTGCGGTT   300
Metarhizium_pingshaense_ARSEF_4342       GGACTCCGAG TAAGTCGGGG AAGAAGTTGG CGGATTTTTT CCCAACCAAC TCAACTT--G ACCAATTT-A CCTGCCCGAG CTCACGGGTA GGCTGCGGTC   300
Metarhizium_pingshaense_ARSEF_7422       GGACTCCGAG TAAGTCGGGG AAGAAGTTGG CGGATTTTTT CCCAACCAAC TCAACTTTTG ACCAATTT-A CCTGCCCGAG CTCACGGGTA GGCTGCGGTT   300
Metarhizium_pingshaense_ARSEF_7929       GGACTCCGAG TAAGTCGGGG AAGAAGTTGG CGGATTTTTT CCCAACCAAC TCAACTTTTG ACCAATTT-A CCTGCCCGAG CTCACGGGTA GGCTGCGGTT   300

mrob-IDF1                                ---------- ---------- ---------- ---------- ---------- ---------- ---------- ---------- ---------- ----------   300
mrob-IDR1                                ---------- ---------- ---------- ---------- ---------- ---------- ---------- ---------- ---------- ----------   300
Metarhizium_robertsii_TYPE_ARSEF_2575    GGACTCCGAG TAAGTCGGGG AAGAAGTTGG CGGATTTTTT CCCAACCAAC TCAACTTTTG ACCAATTT-A CCTGCCCGAG CTCACGGGTA GGCTGCGGTT   300
Metarhizium_robertsii_ARSEF_6472         GGACTCCGAG TAAGTCGGGG AAGAAGTTGG CGGATTTTTT CCCAACCAAC TCAACTTTTG ACCAATTT-A CCTGCCCGAG CTCACGGGTA GGCTGCGGTT   300
Metarhizium_robertsii_ARSEF_727          GGACTCCGAG TAAGTCGGGG AAGAAGTTGG CGGATTTTTT CCCAACCAAC TCAACTTTTG ACCAATTT-A CCTGCCCGAG CTCACGGGTA GGCTGCGGTT   300
Metarhizium_robertsii_ARSEF_7501         GGACTCCGAG TAAGTCGGGG AAGAAGTTGG CGGATTTTTC CCCAACCAAC TCAACTTTTG ACCAATTT-A CCTGCCCGAG CTCACGGGTA GGCTGCGGTT   300
Metarhizium_robertsii_ARSEF_7532         GGACTCCGAG TAAGTCGGGG AAGAAGTTGG CGGATTTTTT CCCAACCAAC TCAACTTTTG ACCAATTT-A CCTGCCCGAG CTCACGGGTA GGCTGCGGTT   300
Metarhizium_robertsii_ARSEF_8070         GGACTCCGAG TAAGTCGGGG AAGAAGTTGG CGGATTTTTT CCCAACCAAC TCAACTTTTG ACCAATTT-A CCTGCCCGAG CTCACGGGTA GGCTGCGGTT   300
Metarhizium_robertsii_ARSEF_8685         GGACTCCGAG TAAGTCGGGG AAGAAGTTGG CGGATTTTTT CCCAACCAAC TCAACTTTTG ACCAATTT-A CCTGCCCGAG CTCACGGGTA GGCTGCGGTT   300
```

```
mani-IDF1                               ---------- ---------- ---------- ---------- ---------- ---------- ---------- ------GGCT ATAGTYAACT TTTGGACTT-   400
mani-IDR1                               ---------- ---------- ---------- ---------- ---------- ---------- ---------- ---------- ---------- ----------   400
Metarhizium_anisopliae_TYPE_ARSEF_7487  TATTTTTGGA CTTT-GTAGA TTTACTTGAA TCAATTCACG GGAAGTTACC TGCCGAATTA CCTGGGCTCC CGGGTAGGCT ATAGTTAACT TTTGGACTT-   400
Metarhizium_anisopliae_ARSEF_1080       TATTTTTGGA CTTT-GTAGA TTTACTTGAA TCAATTCACG GGAAGTTACC TGCCGAATTA CCTGGGCTCC CGGGTAGGCT ATAGTCAACT TTTGGACTT-   400
Metarhizium_anisopliae_ARSEF_6347       TATTTTTGGA CTTT-GTAGA TTTACTTGAA TCAATTCACG GGAAGTTACC TGCCGAATTA CCTGGGCTCC CGGGTAGGCT ATAGTCAACT TTTGGACTT-   400
Metarhizium_anisopliae_ARSEF_7450       TATTTTTGGA CTTT-GTAGA TTTACTTGAA TCAATTCACG GGAAGTTACC TGCCGAATTA CCTGGGCTCC CGGGTAGGCT ATAGTTAACT TTTGGACTT-   400
Metarhizium_anisopliae_ARSEF_798        TATTTTTGGA CTTT-GTAGA TTTACTTGAA TCAATTCACG GGAAGTTACC TGCCGAATTA CCTGGGCTCC CGGGTAGGCT ATAGTCAACT TTTGGACTT-   400
Metarhizium_anisopliae_ART_2062         TATTTTTGGA CTTT-GTAGA TTTACTTGAA TCAATTCACG GGAAGTTACC TGCCGAATTA CCTGGGCTCC CGGGTAGGCT ATAGTTAACT TTTGGACTT-   400

mbru-IDF1                               ---------- ---------- ---------- ---------- ---------- ---------- ---------- ---------- -----TGACT TKTGGACWYG   400
mbru-IDR1                               ---------- ---------- ---------- ---------- ---------- ---------- ---------- ---------- ---------- ----------   400
Metarhizium_brunneum_TYPE_ARSEF_2107    AATTTTTGGA CTTTTGCGGA TTTACTTGAA TCAATTCACA GGAAGTTACC TGCCAAATTG CCTGGGCTCC CGGGTAGGCT ATAGTTGACT TTTGGACTTG   400
Metarhizium_brunneum_ARSEF_1066         AATTTTTGGA CTTT-GCGGA TTTACTTGAA TCAATTCACA GGAAGTTACC TGCCAAATTG CCTGGGCTCC CGGGTAGGCT ATAGTTGACT TTTGGACTTG   400
Metarhizium_brunneum_ARSEF_1278         AATTTTTGGA CTTT-GCGGA TTTACTTGAA TCAATTCACA GGAGGTTACC TGCCAAATTG CCTGGGCTCC CGGGTAGGCT ATAGTTGACT TTTGGACACG   400
Metarhizium_brunneum_ARSEF_3295         AATTTTTGGA CTTT-GCGGG TTCACTCGAA TCAATTCACA GGAAGTTACC TGCCAAATTG CCTGGGCTCC CGGGTAGGCT ATAGTTGACT TTTGGACTTG   400
Metarhizium_brunneum_ARSEF_3826         AATTTTTGGA CTTT-GCGGA TTTACTTGAA TCAATTCACA GGAAGTTACC TGCCAAATTG CCTGGGCTCC CGGGTAGGCT ATAGTTGACT TTTGGACTTG   400
Metarhizium_brunneum_ARSEF_4152         AATTTTTGGA CTTG-GCGGA TTTACTTGAA TCAATCCACA GGAAGTTACC TGCCGAATTA CCTGGGCTCC CGGGTAGGCC ATAGTTGACT TGTGGACTTG   400
Metarhizium_brunneum_ARSEF_4179         AATTTTTGGA CTTG-GCGGA TTTACTTGAA TCAATCCACA GGAAGTTACC TGCCGAATTA CCTGGGCTCC CGGGTAGGCC ATAGTTGACT TGTGGACTTG   400
Metarhizium_brunneum_ARSEF_5198         AATTTTTGGA CTTT-GCGGA TTTACTTGAA TCAATTCACA GGAAGTTACC TGCCAAATTG CCTGGGCTCC CGGGTAGGCT ATAGTTGACT TTTGGACTTG   400
Metarhizium_brunneum_ARSEF_7524         AATTTTTGGA CTTT-GCGGA TTTACTTGAA TCAATTCACA GGAAGTTACC TGCCAAATTG CCTGGGCTCC CGGGTAGGCT ATAGTTGACT TTTGGACTTG   400
Metarhizium_brunneum_ARSEF_988          AATTTTTGGA CTTT-GCGGA TTCACTCGAA TCAATTCACA GGAAGTTACC TGCCAAATTG CCTGGGCTCC CGGGTAGGCT ATAGTTGACT TTTGGACTTG   400
Metarhizium_brunneum_ART_2825           AATTTTTGGA CTTT-GCGGA TTTACTTGAA TCAATTCACA GGAAGTTACC TGCCAAATTG CCTGGGCTCC CGGGTAGGCT ATAGTTGACT TTTGGACTTG   400

mpin-IDF1                               ---------- ---------- ---------- ---------- ---------- ---------- ---------- ---------- ---------- ----------   400
mpin-IDR1                               ---------- ---------- ---------- ---------- ---------- ---------- ---------- ---------- ---------- ----------   400
Metarhizium_pingshaense_TYPE_CBS_257.90 TATTTTTGGA CTTT-GTAGA TTTGCTTGAA TCAATTTACA GGAAGTTGCC TGCCGAATTA CCTGGGCTCC CGGGTAGGCT ATAGTTAACT TTTGGACTT-   400
Metarhizium_pingshaense_ARSEF_2809      TATTTTTGGA CTTT-GTAGA TTTGCTTGAA TCAATTTACA GGAAGTTGCC TGCCGAATTA CCTTGGCTCC CGGGTAGGCT ATAGTTAACT TTTGGACTT-   400
Metarhizium_pingshaense_ARSEF_3210      TATTTTTGGA CTTT-GTAGA TTTGCTTGAA TCAATTTACA GGAAGTTGCC TGCCGAATTA CCTGGGCTCC CGGGTAGGCT ATAGTTAACT TTTGGACTT-   400
Metarhizium_pingshaense_ARSEF_4342      AATTTTTGGA CTTT-GTAGA TTTACTTGAA TCAATTCACA AGAAGTTACC TGCCGAATTA CCTGGGCTCC CGGGTAGGCT ATAGTCAACT TTTGGACTT-   400
Metarhizium_pingshaense_ARSEF_7422      TATTTTTGGA CTTT-GTAGA TTTACTTAAA TCAATTTACA GCAAGTTGCC TGCCGAATTA CCTGGGCTCC CGGGTAGGCT ATAGT-AACT TTTGGACTT-   400
Metarhizium_pingshaense_ARSEF_7929      TATTTTTGGA CTTT-GTAGA TTTGCTTGAA TCAATTTACA GGAAGTTGCC TGCCGAATTA CCTGGGCTCC CGGGTAGGCT ATAGTTAACT TTTGGACTT-   400

mrob-IDF1                               ---------- ---------- ---------- ---------- ---------- ---------- ---------- ---------- ---------- ----------   400
mrob-IDR1                               ---------- ---------- ---------- ---------- ---------- ---------- ---------- ---------- ---------- ----------   400
Metarhizium_robertsii_TYPE_ARSEF_2575   TATTTTTGGA CTTT-GTAGA TTTACTTAAA TCAATTCACA GGAAGTTACC TGCCGAATTA CCTGGGCTCC CGGGTAGGCT ATAGTTAACT TTTGGACTT-   400
Metarhizium_robertsii_ARSEF_6472        TATTTTTGGA CTTT-GTAGA TTTACTTAAA TCAATTCACA GGAAGTTACC TGCCGAATTA CCTGGGCTCC CGGGTAGGCT ATAGTTAACT TTTGGACTT-   400
Metarhizium_robertsii_ARSEF_727         TATTTTTGGA CTTT-GTAGA TTTACTTAAA TCAATTCACA GGAAGTTACC TGCCGAATTA CCTGGGCTCC CGGGTAGGCT ATAGTTAACT TTTGGACTT-   400
Metarhizium_robertsii_ARSEF_7501        TATTTTTGGA CTTT-GTAGA TTTACTTAAA TCAATTCACA GGAAGTTACC TGCCGAATTA CCTGGGCTCC CGGGTAGGCT ATAGTTAACT TTTGGACTT-   400
Metarhizium_robertsii_ARSEF_7532        TATTTTTGGA CTTT-GTAGA TTTACTTAAA TCAATTCACA GGAAGTTACC TGCCGAATTA CCTGGGCTCC CGGGTAGGCT ATAGTTAACT TTTGGACTT-   400
Metarhizium_robertsii_ARSEF_8070        TATTTTTGGA CTTT-GTAGA TTTACTTAAA TCAATTCACA GGAAGTTACC TGCCGAATTA CCTGGGCTCC CGGGTAGGCT ATAGTTAACT TTTGGACTT-   400
Metarhizium_robertsii_ARSEF_8685        TATTTTTGGA CTTT-GTAGA TTTACTTAAA TCAATTCACA GGAAGTTACC TGCCGAATTA CCTGGGCTCC CGGGTAGGCT ATAGTTAACT TTTGGACTT-   400
```

```
mani-IDF1                                  ---------- ---------- ----GC---- ---------- ---------- ---------- ---------- ---------- ---------- ----------   500
mani-IDR1                                  ---------- ---------- ---------- ---------- ---------- ---------- ---------- ---------- ---------- ----------   500
Metarhizium_anisopliae_TYPE_ARSEF_7487     ---------- ---------- ----GCGACT ATAACCTGGG ATTTTCCCAA CCATA--GCT CACGGGTAGG CTAGTTGTGA TTTTTGGACT TGGTGAAATT   500
Metarhizium_anisopliae_ARSEF_1080          ---------- ---------- ----GCGACT ATAACCTGGG ATTTTCCCAA CCAGA--GCT CACGGGTAGG CTAGTTGTGA TTTTTGGACT TGGTGAAATT   500
Metarhizium_anisopliae_ARSEF_6347          ---------- ---------- ----GCGACT ATAACCTGGG ATTTTCCCAA CCAGA--GCT CACGGGTAGG CTAGTTGTGA TTTTTGGACT TGGTGAAATT   500
Metarhizium_anisopliae_ARSEF_7450          ---------- ---------- ----GCGACT ATAACCTGGG ATTTTCCCAA CCATA--GCT CACGGGTAGG CTAGTTGTGA TTTTTGGACT TGGTGAAATT   500
Metarhizium_anisopliae_ARSEF_798           ---------- ---------- ----GCGACT ATAACCTGGG ATTTTCCCAA CCAGA--GCT CACGGGTAGG CTAGTTGTGA TTTTTGGACT TGGTGAAATT   500
Metarhizium_anisopliae_ART_2062            ---------- ---------- ----GCGACT ATAACCTGGG ATTTTCCCAA CCATA--GCT CACGGGTAGG CTAGTTGTGA TTTTTGGACT TGGTGAAATT   500

mbru-IDF1                                  GCGGA----- ---------- ---------- ---------- ---------- ---------- ---------- ---------- ---------- ----------   500
mbru-IDR1                                  ---------- ---------- ---------- ---------- ---------- ---------- ---------- ---------- ---------- ----------   500
Metarhizium_brunneum_TYPE_ARSEF_2107       GCGGATTTTT TTAAACTTAC TTCCCCGACT ATAACCTGGG ATTTTCCCGA CCATA--GCT CACGGGTAGG TAAGTTGTGA TTTTTGGACT TGGTGAAATT   500
Metarhizium_brunneum_ARSEF_1066            GCGGATTTTT TTAAACTTAC TTCCCCGACT ATAACCTGGG ATTTTCCCGA CCATG--GCT CACGGGTAGG TTAGTTGTGA TTTTTGGACT TGGTGAAATT   500
Metarhizium_brunneum_ARSEF_1278            GCGGATTTTT TTAAACTTAC TTCCCCGACT ATAACCTGGG ATTTTCCCGA CCATA--GCT CACGGGTAGG TTAGTTGTGA TTTTTGGAC- -GGTGAAATT   500
Metarhizium_brunneum_ARSEF_3295            GCGGATTTTT TTAAACTTAC TTCCCCCACT ATGACCTGGG ATTTTCCCGA CCATA--GCT CACGGGTAGG TTAGTTGTGA T--------- ----------   500
Metarhizium_brunneum_ARSEF_3826            GCGGATTTTT TTAAACTTAC TTCCCCGACT ATAACCTGGG ATTTTCCCGA CCATG--GCT CACGGGTAGG TTAGTTGTGA TTTTTGGACT TGGTGAAATT   500
Metarhizium_brunneum_ARSEF_4152            GCGGATTTTT TCCAACCTAC TTCCCCGACT ATAACCTGGG ATTTTCCCGA CCATA--GCT CACGGGTAGG TTAGTTGTGA TTTTTGGACT TGGTGAAATT   500
Metarhizium_brunneum_ARSEF_4179            GCGGATTTTT TCCAACCTAC TTCCCCGACT ATAACCTGGG ATTTTCCCGA CCATA--GCT CACGGGTAGG TTAGTTGTGA TTTTTGGACT TGGTGAAATT   500
Metarhizium_brunneum_ARSEF_5198            GCGGATTTTT TTAAACTTAC TTCCCCGACT ATAACCTGGG ATTTTCCCGA CCATG--GCT CACGGGTAGG TTAGTTGTGA TTTTTGGACT TGGTGAAATT   500
Metarhizium_brunneum_ARSEF_7524            GCGGATTTTT TTAAACTTAC TTCCCCGACT ATAACCTGGG ATTTTCCCGA CCATG--GCT CACGGGTAGG TTAGTTGTGA TTTTTGGACT TGGTGAAATT   500
Metarhizium_brunneum_ARSEF_988             GCGGATTTTT TTAAACTTAC TTCCCCCACT ATAACCTGGG ATTTTCCCGA CCATA--GCT CACGGGTAGG TTAGTTGTGA T--------- ----------   500
Metarhizium_brunneum_ART_2825              GCGGATTTTT TTAAACTTAC TTCCCCGACT ATAACCTGGG ATTTTCCCGA CCATA--GCT CACGGGTAGG TTAGTTGTGA T--------- ----------   500

mpin-IDF1                                  ---------- ---------- ---------- ---------- ---------- ---------- ---------- ---------- ---------- ----------   500
mpin-IDR1                                  ---------- ---------- ---------- ---------- ---------- ---------- ---------- ---------- ---------- ----------   500
Metarhizium_pingshaense_TYPE_CBS_257.90    ---------- ---------- ----TCGACT ATAACCTGGG ATTTTCCCAA CTATA--GCT CACGGGTAGG TTAGTTGTGA TTTTTGGAC- -GGTGAAATT   500
Metarhizium_pingshaense_ARSEF_2809         ---------- ---------- ----CCGACT ATAACCTGGG ATTTTCCCAA CTATA--GCT CACGGGTAGG TTAGTTGTGG TTTTTGGACT TGGTGAAATT   500
Metarhizium_pingshaense_ARSEF_3210         ---------- ---------- ----TCGACT ATAACCTGGG ATTTTCCCAA CTATATAGCT CACGGGTAGG TTAGTTGTGA TTTTTGGACT TGGTGAAATT   500
Metarhizium_pingshaense_ARSEF_4342         ---------- ---------- ----CCGACT ATAACCTGGG ATTTTCCCAA CTATA--GCT CACGGGTAGG TTAGTTGTGA TTTTTGGACT TGGTGAAATT   500
Metarhizium_pingshaense_ARSEF_7422         ---------- ---------- ----CCGACT ATAACCTGGG ATTTTCCCAA CTATA--GCT CACGGGTAGG TTAGTTGTGA TTTTTGGAGA CGGTGAAACT   500
Metarhizium_pingshaense_ARSEF_7929         ---------- ---------- ----TCGACT ATAACCTGGG ATTTTCCCAA CTATATAGCT CACGGGTAGG TTAGTTGTGA TTTTTGGACT TGGTGAAATT   500

mrob-IDF1                                  ---------- ---------- ---------- ---------- ---------- ---------- ---------- ---------- ---------- ----------   500
mrob-IDR1                                  ---------- ---------- ---------- ---------- ---------- ---------- ---------- ---------- ---------- ----------   500
Metarhizium_robertsii_TYPE_ARSEF_2575      ---------- ---------- ----CCGACT ATAACCTGGG ATTTTCCCAA CCATA--GCT CACGGGTAGG TTAGTTGTGA TTTTTGGACT CGGTGAAATT   500
Metarhizium_robertsii_ARSEF_6472           ---------- ---------- ----CCGACT ATAACCTGGG ATTTTCCCAA CCATA--GCT CACGGGTAGG TTAGTTGTGA TTTTTGGACT CGGTGAAATT   500
Metarhizium_robertsii_ARSEF_727            ---------- ---------- ----CCGACT ATAACCTGGG ATTTTCCCAA CCATA--GCT CACGGGTAGG TTAGTTGTGA TTTTTGGACT CGGTGAAATT   500
Metarhizium_robertsii_ARSEF_7501           ---------- ---------- ----CCGACT ATAACCTGGG ATTTTCCCAA CCATA--GCT CACGGGTAGG TTAGTTGTGA TTTTTGGACT CGGTGAAATT   500
Metarhizium_robertsii_ARSEF_7532           ---------- ---------- ----CCGACT ATAACCTGGG ATTTTCCCAA CCATA--GCT CACGGGTAGG TTAGTTGTGA TTTTTGGACT CGGTGAAATT   500
Metarhizium_robertsii_ARSEF_8070           ---------- ---------- ----CCGACT ATAACCTGGG ATTTTCCCAA CCATA--GCT CACGGGTAGG TTAGTTGTGA TTTTTGGACT CGGTGAAATT   500
Metarhizium_robertsii_ARSEF_8685           ---------- ---------- ----CCGACT ATAACCTGGG ATTTTCCCAA CCATA--GCT CACGGGTAGG TTAGTTGTGA TTTTTGGACT CGGTGAAATT   500
```

```
mani-IDF1                               ---------- ---------- ---------- ---------- ---------- ---------- ---------- ---------- ---------- ----------   600
mani-IDR1                               ---------- ---------- ---------- ---------- ---------- ---------- ---------- ---------- ---------- ----------   600
Metarhizium_anisopliae_TYPE_ARSEF_7487  TCTACTTTCC TGCCAAGTCT AGCCTACCCG GGAGCCCAGG CAAGTCGGGC AGGTAAATTC CGCCAACTCG GCAGGGAACT AATTGCAAAT CAATTCGAGC   600
Metarhizium_anisopliae_ARSEF_1080       TCTACTTTCC TGCCAAGTCT AGCCTACCCG GGAGCCCAGG CAAGTCGGGC AGGTAAATTC CGCCAACTCG GCAGGGAACT AATTGCAAAT CAATTCGAGC   600
Metarhizium_anisopliae_ARSEF_6347       TCTACTTTCC TGCCAAGTCT AGCCTACCCG GGAGCCCAGG CAAGTCGGGC AGGTAAATTC CGCCAACTCG GCAGGGAACT AATTGCAAAT CAATTCGAGC   600
Metarhizium_anisopliae_ARSEF_7450       TCTACTTTCC TGCCAAGTCT AGCCTACCCG GGAGCCCAGG CAAGTCGGGC AGGTAAATTC CGCCAACTCG GCAGGGAACT AATTGCAAAT CAATTCGAGC   600
Metarhizium_anisopliae_ARSEF_798        TCTACTTTCC TGCCAAGTCT AGCCTACCCG GGAGCCCAGG CAAGTCGGGC AGGTAAATTC CGCCAACTCG GCAGGGAACT AATTGCAAAT CAATTCTAGC   600
Metarhizium_anisopliae_ART_2062         TCTACTTTCC TGCCAAGTCT AGCCTACCCG GGAGCCCAGG CAAGTCGGGC AGGTAAATTC CGCCAACTCG GCAGGGAACT AATTGCAAAT CAATTCGAGC   600

mbru-IDF1                               ---------- ---------- ---------- ---------- ---------- ---------- ---------- ---------- ---------- ----------   600
mbru-IDR1                               ---------- ---------- ---------- ---------- ---------- ---------- ---------- ---------- ---------- ----------   600
Metarhizium_brunneum_TYPE_ARSEF_2107    TCTACTTTCC TGCCAAGTCT AGCCTACCTG GGAGCCCAGG CAAGTCGGGC AGGTAAATTC CGCCAACTCG GCAGGGAACT GATTGCAAAT TAATTTAACC   600
Metarhizium_brunneum_ARSEF_1066         TCTACTTTCC TGCCAAGTCT AGCCTACCTG GGAGCCCAGG CAAGTCGGGC AGGTAAATTC CGCCAACTCG GCAGGGAACT GATTGCAAAT TAATCTAACC   600
Metarhizium_brunneum_ARSEF_1278         TCTACTTTCC TGCCAAGTCT AGCCTACCTG GGAGCCCAGG CAAGTCGGGC AGGTGAATTC CGCCAACTCG GCAGGGAACT GATTGCAAAT TAATTCAACC   600
Metarhizium_brunneum_ARSEF_3295         ---------- ---------- ---------- ---------- ---------- ---------- ---------- ---------- ---------- ----------   600
Metarhizium_brunneum_ARSEF_3826         TCTACTTTCC TGCCAAGTCT AGCCTACCTG GGAGCCCAGG CAAGTCGGGC AGGTAAATTC CGCCAACTCG GCAGGGAACT GATTGCAAAT TAATCTAACC   600
Metarhizium_brunneum_ARSEF_4152         TCTACTTTCC TGCCAAGTCT AGCCTACCCG GGAGCCCAGG CAAGTCGGGC AGGTAAATTC CGCCAACCCG GCAGGGAACT GATTGCAAAT CAATTCAACC   600
Metarhizium_brunneum_ARSEF_4179         TCTACTTTCC TGCCAAGTCT AGCCTACCCG GGAGCCCAGG CAAGTCGGGC AGGTAAATTC CGCCAACCCG GCAGGGAACT GATTGCAAAT CAATTCAACC   600
Metarhizium_brunneum_ARSEF_5198         TCTACTTTCC TGCCAAGTCT AGCCTACCTG GGAGCCCAGG CAAGTCGGGC AGGTAAATTC CGCCAACTCG GCAGGGAACT GATTGCAAAT TAATCTAACC   600
Metarhizium_brunneum_ARSEF_7524         TCTACTTTCC TGCCAAGTCT AGCCTACCTG GGAGCCCAGG CAAGTCGGGC AGGTAAATTC CGCCAACTCG GCAGGGAACT GATTGCAAAT TAATCTAACC   600
Metarhizium_brunneum_ARSEF_988          ---------- ---------- ---------- ---------- ---------- ---------- ---------- ---------- ---------- ----------   600
Metarhizium_brunneum_ART_2825           ---------- ---------- ---------- ---------- ---------- ---------- ---------- ---------- ---------- ----------   600

mpin-IDF1                               ---------- ---------- ---------- ---------- ---------- ---------- ---------- ---------- ---------- ----------   600
mpin-IDR1                               ---------- ---------- ---------- ---------- ---------- ---------- ---------- ---------- ---------- ----------   600
Metarhizium_pingshaense_TYPE_CBS_257.90 TCTACTTTCC TGCCAAGTCT AGCCTACCCG GGAGCCCAGG CAAGTCGGGC AGGTAAATTC CGCCAACTCG GCAGGAAACT AATTGCAAAT CAATTCAAGC   600
Metarhizium_pingshaense_ARSEF_2809      TCTGCTTTCC TGCCAAGTCT AGCCTACCCG GGAGCCCAGG CAAGTCGGGG AGGTAAATTC CGCCAACTCG GCAGGAAACT AATTGCAAAT CAATTCAAGC   600
Metarhizium_pingshaense_ARSEF_3210      TCTACTTTCC TGCCAAGTCT AGCCTACCCG GGAGCCCAGG CAAGTCGGGC AGGTAAATTC CGCCAACTCG GCAGGAAACT AATTGCAAAT CAATTCAAGC   600
Metarhizium_pingshaense_ARSEF_4342      TCTACTTTCC TGCCAAGTCT AGCCTACCCG GGAGCCCAGG CAAGTCGGGC AGGTAAATTC CGCCAACTCG GCAGGAAACT AATTGCAAAT CAATTCAAGC   600
Metarhizium_pingshaense_ARSEF_7422      TCTACTTTCC TGCCAAGTCT AGCCTACCCG GGAGCCCAGG CAAGTCGGGC AGGTAAATTC CGCCAACTCG GCAGGAAACT AATTGCAAAT CAATTCAAGC   600
Metarhizium_pingshaense_ARSEF_7929      TCTACTTTCC TGCCAAGTCT AGCCTACCCG GGAGCCCAGG CAAGTCGGGC AGGTAAATTC CGCCAACTCG GCAGGAAACT AATTGCAAAT CAATTCAAGC   600

mrob-IDF1                               ---------- ---------- ---------- ---------- ---------- ---------- ---------- ---------- ---------- ----------   600
mrob-IDR1                               ---------- ---------- ---------- ---------- ---------- ---------- ---------- ---------- ---------- ----------   600
Metarhizium_robertsii_TYPE_ARSEF_2575   TCTACTTTCC TGCCAAGTCT AGTCTACCCG GGAGCCCAGG CAAGTCGGGC AGGTAAATTC CGCCAACTCG GCAGGGAACT AATTGCAAAT CAATTCAAGC   600
Metarhizium_robertsii_ARSEF_6472        TCTACTTTCC TGCCAAGTCT AGTCTACCCG GGAGCCCAGG CAAGTCGGGC AGGTAAATTC CGCCAACTCG GCAGGGAACT AATTGCAAAT CAATTCAAGC   600
Metarhizium_robertsii_ARSEF_727         TCTACTTTCC TGCCAAGTCT AGTCTACCCG GGAGCCCAGG CAAGTCGGGC AGGTAAATTC CGCCAACTCG GCAGGGAACT AATTGCAAAT CAATTCAAGC   600
Metarhizium_robertsii_ARSEF_7501        TCTACTTTCC TGCCAAGTCT AGTCTACCCG GGAGCCCAGG CAAGTCGGGC AGGTAAATTC CGCCAACTCG GCAGGGAACT AATTGCAAAT CAATTCAAGC   600
Metarhizium_robertsii_ARSEF_7532        TCTACTTTCC TGCCAAGTCT AGTCTACCCG GGAGCCCAGG CAAGTCGGGC AGGTAAATTC CGCCAACTCG GCAGGGAACT AATTGCAAAT CAATTCAAGC   600
Metarhizium_robertsii_ARSEF_8070        TCTACTTTCC TGCCAAGTCT AGTCTACCCG GGAGCCCAGG CAAGTCGGGC AGGTAAATTC CGCCAACTCG GCAGGGAACT AATTGCAAAT CAATTCAAGC   600
Metarhizium_robertsii_ARSEF_8685        TCTACTTTCC TGCCAAGTCT AGTCTACCCG GGAGCCCAGG CAAGTCGGGC AGGTAAATTC CGCCAACTCG GCAGGGAACT AATTGCAAAT CAATTCAAGC   600
```

```
mani-IDF1                              ---------- ---------- ---------- ---------- ---------- ---------- ---------- ---------- ---------- ----------   700
mani-IDR1                              ---------- ---------- ---------- ---------- ---------- ---------- ---------- ---------- ---------- ----------   700
Metarhizium_anisopliae_TYPE_ARSEF_7487 AAATATGCCA AGTCCACAAA TGAATCGCAG CCTACCCGGT AGGTAAGCCT GTGTAGTTTC CGTGTAATTT TCCCGTAAAG ACCAAAAAGT CAGGTGTTTT   700
Metarhizium_anisopliae_ARSEF_1080      AAATGTGCCA AGTCCACAAA TTAATCGTAG CCTACCCGGT AGGTAAGCCT GTGTAGTTTC CGTGTAATTT TCCCGCAAAG ACCAAAAAGT CAGGTGTTTT   700
Metarhizium_anisopliae_ARSEF_6347      AAATGTGCCA AGTCCACAAA TTAATCGTAG CCTACCCGGT AGGTAAGCCT GTGTAGTTTC CGTGTAATTT TCCCGCAAAG ACCAAAAAGT CAGGTGTTTT   700
Metarhizium_anisopliae_ARSEF_7450      AAATATGCCA AGTCCACAAA TGAATCGCAG CCTACCCGGT AGGTAAGCCT GTGTAGTTTC CGTGTAATTT TCCCGTAAAG ACCAAAAAGT CAGGTGTTTT   700
Metarhizium_anisopliae_ARSEF_798       AAATGTGCCA AGTCCACAAA TTAATCGTAG CCTACCCGGT AGGTAAGCCT GTGTAGTTTC CGTGTAATTT TCCCGCAAAG ACCAAAAAGT CAGGTGTTTT   700
Metarhizium_anisopliae_ART_2062        AAATATGCCA AGTCCACAAA TGAATCGCAG CCTACCCGGT AGGTAAGCCT GTGTAGTTTC CGTGTAATTT TCCCGTAAAG ACCAAAAAGT CAGGTGTTTT   700

mbru-IDF1                              ---------- ---------- ---------- ---------- ---------- ---------- ---------- ---------- ---------- ----------   700
mbru-IDR1                              ---------- ---------- ---------- ---------- ---------- ---------- ---------- ---------- ---------- ----------   700
Metarhizium_brunneum_TYPE_ARSEF_2107   AAATCTGCCA AGTCCACAAG TTAACTATAG CCTACCCGGG A----GGCCT GGGTGTTTCC CGCGTGATTT -CCTGTAAAA ACAAAAAAGT TAGGTGTTTT   700
Metarhizium_brunneum_ARSEF_1066        AAATCTGCCA AGTCCACAAG TTAACTATAG CCTACCCGGT A----GACCT GGGTGTTTCC CGCGTGATTT -CCTGTGAAA ACAAAAAAGT TAGGTGTTTT   700
Metarhizium_brunneum_ARSEF_1278        AAATCTGCCA AGTCCACAAG TTAACTATAG CTTACCCGGT A----GGCCT GGGTGCTTCC CGCGTGGTTT -CCTGTAAAA ACAAAAAAGT TAGGTGTTTT   700
Metarhizium_brunneum_ARSEF_3295        ---------A AGTCCACAAA TAAATTACAG CCTACCCGGT A----GGCCT GTATATTTCC CGCGCGATTT -CCTGTGAAG ACCAAAAAGT TAGGTGTTTT   700
Metarhizium_brunneum_ARSEF_3826        AAATCTGCCA AGTCCACAAG TTAACTATAG CCTACCCGGT A----GACCT GGGTGTTTCC CGCGTGATTT -CCTGTGAAA ACAAAAAAGT TAGGTGTTTT   700
Metarhizium_brunneum_ARSEF_4152        AAATCTGCCA AGTCCACAGA TAAACTATAG CCTACCCGGT A----GGCCT GTAGATTTCC CGCGCGATTT -CCTGCGAAG ACCAAAAAGT TAGGTGTTTT   700
Metarhizium_brunneum_ARSEF_4179        AAATCTGCCA AGTCCACAGA TAAACTATAG CCTACCCGGT A----GGCCT GTAGATTTCC CGCGCGATTT -CCTGCGAAG ACCAAAAAGT TAGGTGTTTT   700
Metarhizium_brunneum_ARSEF_5198        AAATCTGCCA AGTCCACAAG TTAACTATAG CCTACCCGGT A----GACCT GGGTGTTTCC CGCGTGATTT -CCTGTGAAA ACAAAAAAGT TAGGTGTTTT   700
Metarhizium_brunneum_ARSEF_7524        AAATCTGCCA AGTCCACAAG TTAACTATAG CCTACCCGGT A----GACCT GGGTGTTTCC CGCGTGATTT -CCTGTGAAA ACAAAAAAGT TAGGTGTTTT   700
Metarhizium_brunneum_ARSEF_988         ---------A AGTCCACAAA TAAATTACAG CCTACCCGGT A----GGCCT GTATATTTCC CGCGCGATTT -CCTGTGAAG ACCAAAAAGT TAGGTGTTTT   700
Metarhizium_brunneum_ART_2825          ---------A AGTCCACAAA TAAATTACAG CCTACCCGGT A----GGCCT GTATATTTCC CGCGCGATTT -CCTGTGAAG ACCAAAAAGT TAGGTGTTTT   700

mpin-IDF1                              ---------- ---------- ---------- ---------- ---------- ---------- ---------- ---------- ---------- ----------   700
mpin-IDR1                              ---------- ---------- ---------- ---------- ---------- ---------- ---------- ---------- ---------- ----------   700
Metarhizium_pingshaense_TYPE_CBS_257.90 AAATATGCCA AGTCCACAAA TCAATCGCAG CCTACCCGGT A--------- ---------- ---------- ---------- ---------- ----------   700
Metarhizium_pingshaense_ARSEF_2809     AAATATGCCA AGTCCACAAA TCAACCGCAG CCTACCCGGT A--------- ---------- ---------- ---------- ---------- ----------   700
Metarhizium_pingshaense_ARSEF_3210     AAATATGCCA AGTCCACAAA TCAATCGCAG CCTACCCGGT A--------- ---------- ---------- ---------- ---------- ----------   700
Metarhizium_pingshaense_ARSEF_4342     AAATATGCCA AGTCCACAAA TTAATCGCAG CCTACCCGGT A----AGCCT GTGTAATTT- ---------- ---------- ---------- ----------   700
Metarhizium_pingshaense_ARSEF_7422     AAATATGCCA AGTCCACAAA TTAATCGCAG CCTACCCGGT A----AGCCT GTGTAATTTC TGTGTAATTT -CCCGTAAAG ACCAAAAAGT TGGGTATTTT   700
Metarhizium_pingshaense_ARSEF_7929     AAATATGCCA AGTCCACAAA TCAATCGCAG CCTACCCGGT A--------- ---------- ---------- ---------- ---------- ----------   700

mrob-IDF1                              ---------- ---------- ---------- ---------- ---------- ---------- ---------- ---------- ---------- ----------   700
mrob-IDR1                              ---------- ---------- ---------- ---------- ---------- ---------- ---------- ---------- ---------- ----------   700
Metarhizium_robertsii_TYPE_ARSEF_2575  AAATGTGCCA AGTCCACAAA TCAATCGCAG CCTACCCGGT AGGTAAGCCT GTGTAGTTTC CGAGTAATTT TCCCGTAAAG ACCAAAAAGT CAGGTATTTT   700
Metarhizium_robertsii_ARSEF_6472       AAATGTGCCA AGTCCACAAA TCAATCGCAG CCTACCCGGT AGGTAAGCCT GTGTGATTTC CGTGTAATTT -CCCGTAAAG ACCAAAAAGT TAGGTATTTT   700
Metarhizium_robertsii_ARSEF_727        AAATGTGCCA AGTCCACAAA TCAATCGCAG CCTACCCGGT AGGTAAGCCT GTGTGATTTC CGTGTAATTT -CCCGTAAAG ACCAAAAAGT TAGGTATTTT   700
Metarhizium_robertsii_ARSEF_7501       AAATGTGCCA AGTCCACAAA TCAATCGCAG CCTACCTGGT AGGTAAGCCT GTGTAGTTTC CGTGTAATTT TCCCGTAAAG ACCAAAAAGT CAGGTATTTT   700
Metarhizium_robertsii_ARSEF_7532       AAATGTGCCA AGTCCACAAA TCAATCGCAG CCTACCCGGT AGGTAAGCCT GTGTGATTTC CGTGTAATTT -CCCGTAAAG ACCAAAAAGT TAGGTATTTT   700
Metarhizium_robertsii_ARSEF_8070       AAATGTGCCA AGTCCACAAA TCAATCGCAG CCTACCCGGT AGGTAAGCCT GTGTGATTTC CGTGTAATTT -CCCGTAAAG ACCAAAAAGT TAGGTATTTT   700
Metarhizium_robertsii_ARSEF_8685       AAATGTGCCA AGTCCACAAA TCAATCGCAG CCTACCCGGT AGGTAAGCCT GTGTAGTTTC CGAGTAATTT TCCCGTAAAG ACCAAAAAGT CAGGTATTTT   700
```

```
mani-IDF1                              ---------- ---------- ---------- ---------- ---------- ---------- ---------- ---------- ---------- ----------   800
mani-IDR1                              ---------- --ATATAGGC GTGAGTTGAT TTTTTTG-T- ---------- ---------- ---------- ---------- ---------- ----------   800
Metarhizium_anisopliae_TYPE_ARSEF_7487 AGTATTTAAT TTATATAGGC GTGAGTTGAT TTTTTTG-TT TTTCTATATT AATAAATTTT GCGGAAAATA AAAAATAAGC TATAAAAACC TGGTAACAGA   800
Metarhizium_anisopliae_ARSEF_1080      AGTATTTAAT TTATATAGGC GTGAGTTGAT TTTTTTG-TT TTTTTATATT AATAAATTTT GCGGAAAATA AAAAATAAGC TATAAAAACC TGGTAACAAA   800
Metarhizium_anisopliae_ARSEF_6347      AGTATTTAAT TTATATAGGC GTGAGTTGAT TTTTTTG-TT TTTTTATATT AATAAATTTT GCGGAAAATA AAAAATAAGC TATAAAAACC TGGTAACAAA   800
Metarhizium_anisopliae_ARSEF_7450      AGTATTTAAT TTATATAGGC GTGAGTTGAT TTTTTTG-TT TTTCTATATT AATAAATTTT GCGGAAAATA AAAAATAAGC TATAAAAACC TGGTAACAGA   800
Metarhizium_anisopliae_ARSEF_798       AGTATTTAAT TTATATAGGC GTGAGTTGAT TTTTTTG-TT TTTTTATATT AATAAATTTT GCGGAAAATA AAAAATAAGC TATAAAAACC TGGTAACAAA   800
Metarhizium_anisopliae_ART_2062        AGTATTTAAT TTATATAGGC GTGAGTTGAT TTTTTTG-TT TTTCTATATT AATAAATTTT GCGGAAAATA AAAAATAAGC TATAAAAACC TGGTAACAGA   800

mbru-IDF1                              ---------- ---------- ---------- ---------- ---------- ---------- ---------- ---------- ---------- ----------   800
mbru-IDR1                              ---------- ---------- ---------- ---------- ---------- ---------- ---------- --------AC CACGAGAGCC YRGTAGCG--   800
Metarhizium_brunneum_TYPE_ARSEF_2107   AGTACTTAAT TTATATAGGT ATAAGTTAAT TTTTTTG-TT TTCTTATATA AATAAAATTT GCGGAAAATA AAAAATAAAC CACGAGAGCC CGGTAGCGGA   800
Metarhizium_brunneum_ARSEF_1066        AGTACTTAAT TTATGTAGGT ATGGGTTGAT TTTTTTG-TT TTCTTATATA AATAAAATTT GCGGAAAATA AAAAATAAAC CACGAGAGCC CGGTAGCGGA   800
Metarhizium_brunneum_ARSEF_1278        AGTACTTAAT TTATATAGGT ATAAGTTGAT TTTTTTG-TT TTCTTATATA AATAAAATTT GCGGAAAATA AAAAATAAAC CACGAGAGCC CGGTAGCGGA   800
Metarhizium_brunneum_ARSEF_3295        AGTACTTAAT TTATATAGGT ATAAGTTGAT TTTTTTGTTT TTTTTATATA TATAAATTTT GCGGGAAATA AAAAATAAAC CACGAGAGCC CGGTAGCGGA   800
Metarhizium_brunneum_ARSEF_3826        AGTACTTAAT TTATATAGGT ATGGGTTGAT TTTTTTG-TT TTCTTATATA AATAAAATTT GCGGAAAATA AAAAATAAAC CACGAGAGCC CGGTAGCGGA   800
Metarhizium_brunneum_ARSEF_4152        GGTACTTAAT TTATATAGAG ATGAGTTGAT TTTTTTG-TT TTTATATATA AATAAATTTT GCGGAAAATA AAAAATAAAC CACGAGAGCC TAGTAGCGGA   800
Metarhizium_brunneum_ARSEF_4179        GGTACTTAAT TTATATAGAG ATGAGTTGAT TTTTTTG-TT TTTATATATA AATAAATTTT GCGGAAAATA AAAAATAAAC CACGAGAGCC TAGTAGCGGA   800
Metarhizium_brunneum_ARSEF_5198        AGTACTTAAT TTATGTAGGT ATGGGTTGAT TTTTTTG-TT TTCTTATATA AATAAAATTT GCGGAAAATA AAAAATAAAC CACGAGAGCC CGGTAGCGGA   800
Metarhizium_brunneum_ARSEF_7524        AGTACTTAAT TTATGTAGGT ATGGGTTGAT TTTTTTG-TT TTCTTATATA AATAAAATTT GCGGAAAATA AAAAATAAAC CACGAGAGCC CGGTAGCGGA   800
Metarhizium_brunneum_ARSEF_988         AGTACTTAAT TTATATAGGT ATAAGTTGAT TTTTTTGTTT TTTTTATATA TATAAATTTT GCGGGAAATA AAAAATAAAC CACGAGAGCC CGGTAGCGGA   800
Metarhizium_brunneum_ART_2825          AGTACTTAAT TTATATAGGT ATAAGTTGAT TTTTTTG-TT TTTATATATA TATAAATTTT GCGGAAAATA AAAAATAAAC CACGAGAGCC CGGTAGCGGA   800

mpin-IDF1                              ---------- ---------- ---------- ---------- ---------- ---------- ---------- ---------- ---------- ----------   800
mpin-IDR1                              ---------- ---------- ---------- ---------- ---------- ---------- ---------- ---------- ---------- ----------   800
Metarhizium_pingshaense_TYPE_CBS_257.90 ---------- -------AGT ATAAGTTGAT TTTTTT--TT -ATATATATA AATAAATTCT GCGGAAAATA AAAAATAAGC TATAAAGGCC TGGTAGCGGA   800
Metarhizium_pingshaense_ARSEF_2809     ---------- -------AGT ATAAGTTGAT CTTTTT--TT TATATATGTA AATAAATTTT GCGGAAAATA AAAAATAAGC TATAAAAGCC TGGTAGCGGA   800
Metarhizium_pingshaense_ARSEF_3210     ---------- -------AGT ATAAGTTGAT TTTTTT--TT TATATATATA AATAAATTTT GCGGAAAATA AAAAATAAGC TATAAAGGCC TGGTAGCGGA   800
Metarhizium_pingshaense_ARSEF_4342     ---------- ---------- ---------- ---------- ---------- -------TTT GCGGAAAATA AAAAATAAGC TATAAAAGCC TGGTAGCGGA   800
Metarhizium_pingshaense_ARSEF_7422     AGTACTTAAT TTATATAAGT ATAAGTTGAT TTTTTTG-TT TTTTTATATA AATAAATTTT GCGGAAAATA AAAAATAAGC TATAAAAGCC TGGTAGCGCA   800
Metarhizium_pingshaense_ARSEF_7929     ---------- -------AGT ATAAGTTGAT TTTTTT--TT TATATATATA AATAAATTTT GCGGAAAATA AAAAATAAGC TATAAAGGCC TGGTAGCGGA   800

mrob-IDF1                              ---------- ---------- ---------- ---------- ---------- ---------- ---------- ---------- ---------- ----------   800
mrob-IDR1                              ---------- ---------- ---------- ---------- ---------- ---------- ---------- ---------- ---------- ----------   800
Metarhizium_robertsii_TYPE_ARSEF_2575  GGTACTTAAT TTATATAAGT ACAAGTTGAT TTTTTTG-TT TTTTTATATA AATAAATTTT GCGGAAAATA AAAAATAAGC TATAAAAGCC TGGTAGCGGA   800
Metarhizium_robertsii_ARSEF_6472       GGTACTTAAT TTATATAAGT ACAAGTTGAT TTTTTTG-TT TTTTTATATA AATAAATTTT GCGGAAAATA AAAAATAAGC TATAAAAGCC TGGTAGCGGA   800
Metarhizium_robertsii_ARSEF_727        GGTACTTAAT TTATATAAGT ACAAGTTGAT TTTTTTG-TT TTTTTATATA AATAAATTTT GCGGAAAATA AAAAATAAGC TATAAAAGCC TGGTAGCGGA   800
Metarhizium_robertsii_ARSEF_7501       GGTACTTAAT TTATATAAGT ACAAGTTGAT TTTTTTG-TT TTTTTATATA AATAAATTTT GCGGAAAATA AAAAATAAGC TATAAAAGCC TGGTAGCGGA   800
Metarhizium_robertsii_ARSEF_7532       GGTACTTAAT TTATATAAGT ACAAGTTGAT TTTTTTG-TT TTTTTATATA AATAAATTTT GCGGAAAATA AAAAATAAGC TATAAAAGCC TGGTAGCGGA   800
Metarhizium_robertsii_ARSEF_8070       GGTACTTAAT TTATATAAGT ACAAGTTGAT TTTTTTG-TT TTTTTATATA AATAAATTTT GCGGAAAATA AAAAATAAGC TATAAAAGCC TGGTAGCGGA   800
Metarhizium_robertsii_ARSEF_8685       GGTACTTAAT TTATATAAGT ACAAGTTGAT TTTTTTG-TT TTTTTATATA AATAAATTTT GCGGAAAATA AAAAATAAGC TATAAAAGCC TGGTAGCGGA   800
```

```
mani-IDF1                              ---------- ---------- ---------- ---------- ---------- ---------- ---------- ---------- ---------- ----------   900
mani-IDR1                              ---------- ---------- ---------- ---------- ---------- ---------- ---------- ---------- ---------- ----------   900
Metarhizium_anisopliae_TYPE_ARSEF_7487 GGCGGGCCCT TAAGGTAATT GGTGGGTATA TAAGAGGGAG GGCTGGACAC GCGTTGCGTT GTGTATTTCC ACCACACCCA ATACTTTTAG CTTTAGGGTA   900
Metarhizium_anisopliae_ARSEF_1080      GGCGGGCTTT TAAGGTAATT GGTGGGTATA TAAGAGGGAG GGCTGGACAC GCGTTGCGTT GTGTATTTCT ACCATACCCA ATACTTTTAG CTTTAGGGTA   900
Metarhizium_anisopliae_ARSEF_6347      GGCGGGCTTT TAAGGTAATT GGTGGGTATA TAAGAGGGAG GGCTGGACAC GCGTTGCGTT GTGTATTTCT ACCATACCCA ATACTTTTAG CTTTAGGGTA   900
Metarhizium_anisopliae_ARSEF_7450      GGCGGGCCCT TAAGGTAATT GGTGGGTATA TAAGAGGGAG GGCTGGACAC GCGTTGCGTT GTGTATTTCC ACCACACCCA ATACTTTTAG CTTTAGGGTA   900
Metarhizium_anisopliae_ARSEF_798       GGCGGGCTTT TAAGGTAATT GGTGGGTATA TAAGAGGGAG GGCTGGACAC GCGTTGCGTT GTGTATTTCT ACCATACCCA ATACTTTTAG CTTTAGGGTA   900
Metarhizium_anisopliae_ART_2062        GGCGGGCCCT TAAGGTAATT GGTGGGTATA TAAGAGGGAG GGCTGGACAC GCGTTGCGTT GTGTATTTCC ACCACACCCA ATACTTTTAG CTTTAGGGTA   900

mbru-IDF1                              ---------- ---------- ---------- ---------- ---------- ---------- ---------- ---------- ---------- ----------   900
mbru-IDR1                              ---------- ---------- ---------- ---------- ---------- ---------- ---------- ---------- ---------- ----------   900
Metarhizium_brunneum_TYPE_ARSEF_2107   GGCAAGCCCT TAAGGTAGTC GGTAAGTATA TAAGAGAGAG GGCTGGACAC GCGTCGCGTC GCGTATTTCC ACCGCACCTA ATACTTTTGG CTTTAGGGTA   900
Metarhizium_brunneum_ARSEF_1066        GGCGAGCCCT TAAGGTAGTC GGTAAGTATA TAAGAGAGAG GGCTGGACAC GCGTCGCGTC GCGTATTTCC ACCGCACCTA ATACTTTTGG CTTTAGGGTA   900
Metarhizium_brunneum_ARSEF_1278        GGCGAGCCCT TAAGGTAGTC GGTAAGTATA TAAGAGAGAG GGCTGGACAC GCGTCGCGTC GCGTATTTCC ACCGCACCTA ATACTTTTGG CTTTAGGGTA   900
Metarhizium_brunneum_ARSEF_3295        GGCGAGCCCT TAAGGTAGTC GGTAAGTATA TAAGAGAGAG GGCTGGACAC GCGTCGCGTC GCGTATTTCC ACCGCACCTA ATACTTTTGG CTTTAGGGTA   900
Metarhizium_brunneum_ARSEF_3826        GGCGAGCCCT TAAGGTAGTC GGTAAGTATA TAAGAGAGAG GGCTGGACAC GCGTCGCGTC GCGTATTTCC ACCGCACCTA ATACTTTTGG CTTTAGGGTA   900
Metarhizium_brunneum_ARSEF_4152        GGCGGGCCCT CGAGGTGGTC GGTAGGTATA TAAGAGAGAG GGCTGGACAC GCGTCGCGTC GCGTATTTCC ACCGCAGCCA ATACCTTTAG CTTTAGGGTA   900
Metarhizium_brunneum_ARSEF_4179        GGCGGGCCCT CGAGGTGGTC GGTAGGTATA TAAGAGAGAG GGCTGGACAC GCGTCGCGTC GCGTATTTCC ACCGCAGCCA ATACCTTTAG CTTTAGGGTA   900
Metarhizium_brunneum_ARSEF_5198        GGCGAGCCCT TAAGGTAGTC GGTAAGTATA TAAGAGAGAG GGCTGGACAC GCGTCGCGTC GCGTATTTCC ACCGCACCTA ATACTTTTGG CTTTAGGGTA   900
Metarhizium_brunneum_ARSEF_7524        GGCGAGCCCT TAAGGTAGTC GGTAAGTATA TAAGAGAGAG GGCTGGACAC GCGTCGCGTC GCGTATTTCC ACCGCACCTA ATACTTTTGG CTTTAGGGTA   900
Metarhizium_brunneum_ARSEF_988         GGCGAGCCCT TAAGGTAGTC GGTAAGTATA TAAGAGAGAG GGCTGGACAC GCGTCGCGTC GCGTATTTCC ACCGCACCTA ATACTTTTGG CTTTAGGGTA   900
Metarhizium_brunneum_ART_2825          GGCGAGCCCT TAAGGTAGTC GGTAAGTATA TAAGAGAGAG GGCTGGACAC GCGTCGCGTC GCGTATTTCC ACCGCACCTA ATACTTTTGG CTTTAGGGTA   900

mpin-IDF1                              ---------- ---------- ---------- ---------- ---------- ---------- ---------- ---------- ---------- ----------   900
mpin-IDR1                              ---------- ---------- ---------- ---------- ---------- ---------- ---------- ---------- ---------- ----------   900
Metarhizium_pingshaense_TYPE_CBS_257.90 GGCGAGCTCT TAAGGTAGTG GGTGGGTATA TAAGAGGGAG GGCTGGACAC GCGTTGCGCC GCGTATTTCC ACCGCACCCA ATACTTTTAG CTTTAGGGTA   900
Metarhizium_pingshaense_ARSEF_2809     GGCGAGCTCT TAAGATAGTT GGTGGGTATA TAAGAGGGAG GGCTGGACAC GCGTTACGCC GCGTATTTCC ACCGCACCCA ATACTTTTAG CTTTAGGGTA   900
Metarhizium_pingshaense_ARSEF_3210     GGCGAGCTCT TAAGGTAGTG GGTGGGTATA TAAGAGGGAG GGCTGGACAC GCGTTGCGCC GCGTATTTCC ACCGCACCCA ATACTTTTAG CTTTAGGGTA   900
Metarhizium_pingshaense_ARSEF_4342     GGCGGGCTCC TAAGGTAGTT GGTGGGTATA TAAGAGGGAG GGCTGGACAC GCGTTGCGTC GCGTATTTCC ACCGCACCCA ATACTTTTAG CTTTAGGGTA   900
Metarhizium_pingshaense_ARSEF_7422     GGCGGGCTCT TAAGGTAGTT GGTGGGTATA TAAGAGGGAG GGCTGGAGAC GCGTTGCGCC CCGTATTTCC ACCGTACCCA ATACTTTTAG CTTTAGGGTA   900
Metarhizium_pingshaense_ARSEF_7929     GGCGAGCTCT TAAGGTAGTG GGTGGGTATA TAAGAGGGAG GGCTGGACAC GCGTTGCGCC GCGTATTTCC ACCGCACCCA ATACTTTTAG CTTTAGGGTA   900

mrob-IDF1                              ---------- ---------- ---------- ---------- ---------- ---------- ---------- ---------- ---------- ----------   900
mrob-IDR1                              ---------- ---------- ---------- ---------- ---------- ---------- ---------- GCCGCAGCCA ATACTTTTAG ----------   900
Metarhizium_robertsii_TYPE_ARSEF_2575  GGCGGGCCCT TAGGGTAGTT GGTGGGTATA TGAGAGGGAG GGCTGGACAC GCGTCGCGTC GCGTATTTCC GCCGCAGCCA ATACTTTTAG CTTTAGGGTA   900
Metarhizium_robertsii_ARSEF_6472       GGCGGGCCCT TAGGGTAGTT GGTGGGTATA TGAGAGGGAG GGCTGGACAC GCGTCGCGTC GCGTATTTCC GCCGCAGCCA ATACTTTTAG CTTTAGGGTA   900
Metarhizium_robertsii_ARSEF_727        GGCGGGCCCT TAGGGTAGTT GGTGGGTATA TGAGAGGGAG GGCTGGACAC GCGTCGCGTC GCGTATTTCC GCCGCAGCCA ATACTTTTAG CTTTAGGGTA   900
Metarhizium_robertsii_ARSEF_7501       GGCGGGCCCT TAGGGTAGTT GGTGGGTATA TGAGAGGGAG GGCTGGACAC GCGTCGCGTC GCGTATTTCC GCCGCAGCCA ATACTTTTAG CTTTAGGGTA   900
Metarhizium_robertsii_ARSEF_7532       GGCGGGCCCT TAGGGTAGTT GGTGGGTATA TGAGAGGGAG GGCTGGACAC GCGTCGCGTC GCGTATTTCC GCCGCAGCCA ATACTTTTAG CTTTAGGGTA   900
Metarhizium_robertsii_ARSEF_8070       GGCGGGCCCT TAGGGTAGTT GGTGGGTATA TGAGAGGGAG GGCTGGACAC GCGTCGCGTC GCGTATTTCC GCCGCAGCCA ATACTTTTAG CTTTAGGGTA   900
Metarhizium_robertsii_ARSEF_8685       GGCGGGCCCT TAGGGTAGTT GGTGGGTATA TGAGAGGGAG GGCTGGACAC GCGTCGCGTC GCGTATTTCC GCCGCAGCCA ATACTTTTAG CTTTAGGGTA   900
```

```
mani-IDF1                                ---------- ---------- ---------- ---------- ---------- ---------- ---------- ---------- ---------- ----------  1000
mani-IDR1                                ---------- ---------- ---------- ---------- ---------- ---------- ---------- ---------- ---------- ----------  1000
Metarhizium_anisopliae_TYPE_ARSEF_7487   GGCTGCTTGT TCAGAGGCGT GCTGAATTAA ATGGTCTCTT GAGTGAGGGG ATTTCTCTGC TGGCAGTTGC CTGTGATCCG GGAGTCCGTG GCGGTAAAGT  1000
Metarhizium_anisopliae_ARSEF_1080        GGCTGCTTGT TTAGAGGCGT GCTGAATTAA ATGGTCTCTT GAGTGAGGGG ATTTCTCTGC TGGCAGTTGC CTGTGATCCG GGAGTCCGTG GCGGTAAAGT  1000
Metarhizium_anisopliae_ARSEF_6347        GGCTGCTTGT TTAGAGGCGT GCTGAATTAA ATGGTCTCTT GAGTGAGGGG ATTTCTCTGC TGGCAGTTGC CTGTGATCCG GGAGTCCGTG GCGGTAAAGT  1000
Metarhizium_anisopliae_ARSEF_7450        GGCTGCTTGT TCAGAGGCGT GCTGAATTAA ATGGTCTCTT GAGTGAGGGG ATTTCTCTGC TGGCAGTTGC CTGTGATCCG GGAGTCCGTG GCGGTAAAGT  1000
Metarhizium_anisopliae_ARSEF_798         GGCTGCTTGT TTAGAGGCGT GCTGAATTAA ATGGTCTCTT GAGTGAGGGG ATTTCTCTGC TGGCAGTTGC CTGTGATCCG GGAGTCCGTG GCGGTAAAGT  1000
Metarhizium_anisopliae_ART_2062          GGCTGCTTGT TCAGAGGCGT GCTGAATTAA ATGGTCTCTT GAGTGAGGGG ATTTCTCTGC TGGCAGTTGC CTGTGATCCG GGAGTCCGTG GCGGTAAAGT  1000

mbru-IDF1                                ---------- ---------- ---------- ---------- ---------- ---------- ---------- ---------- ---------- ----------  1000
mbru-IDR1                                ---------- ---------- ---------- ---------- ---------- ---------- ---------- ---------- ---------- ----------  1000
Metarhizium_brunneum_TYPE_ARSEF_2107     GGCTGCTTGT TTAGAGGCGC GCTGAATTAG ATGGTCTCTT AAGTGAGGGG ATTTCTCTGC TGGCAGTTGC CTGTAATCCG GGAGTCCGTG GTGGTAAAGT  1000
Metarhizium_brunneum_ARSEF_1066          GGCTGCTTGT TTAGAGGCGC GCTGAATTAG ATGGTCTCTT AAGTGAGGGG ATTTCTCTGG TGGCAGTTGC CTGTAATCCG GGAGCCCGTG GTGGTAAAGT  1000
Metarhizium_brunneum_ARSEF_1278          GGCTGCTTGT TTAGAGGCGC GCTGAATTAG ATGGTCTCTT AAGTGAGGGG ATTTCTCTGC TGGCAGTTGC CTGTAATCCG GGAGTCCGTG GTGGTAAAGT  1000
Metarhizium_brunneum_ARSEF_3295          GGCTGCTTGT TTAGAGGCGC GCTGAATTAG ATGGTCTCTT AAGTGAGGGG ATTTCTCTGC TGGCAGTTGC CTGTAATCCG GGAGTCCGTG GTGGTAAAGT  1000
Metarhizium_brunneum_ARSEF_3826          GGCTGCTTGT TTAGAGGCGC GCTGAATTAG ATGGTCTCTT AAGTGAGGGG ATTTCTCTGG TGGCAGTTGC CTGTAATCCG GGAGCCCGTG GTGGTAAAGT  1000
Metarhizium_brunneum_ARSEF_4152          GGCTGCTTGT TTAGAGGCGC GCTGGATTAG ATGGTCTCTC AAGTGAGGGG ATTTCTCTGC TGGCAGTTGC CTATAATCCG GGAGTCCGTG GTGGTAAAAT  1000
Metarhizium_brunneum_ARSEF_4179          GGCTGCTTGT TTAGAGGCGC GCTGGATTAG ATGGTCTCTC AAGTGAGGGG ATTTCTCTGC TGGCAGTTGC CTATAATCCG GGAGTCCGTG GTGGTAAAAT  1000
Metarhizium_brunneum_ARSEF_5198          GGCTGCTTGT TTAGAGGCGC GCTGAATTAG ATGGTCTCTT AAGTGAGGGG ATTTCTCTGG TGGCAGTTGC CTGTAATCCG GGAGCCCGTG GTGGTAAAGT  1000
Metarhizium_brunneum_ARSEF_7524          GGCTGCTTGT TTAGAGGCGC GCTGAATTAG ATGGTCTCTT AAGTGAGGGG ATTTCTCTGG TGGCAGTTGC CTGTAATCCG GGAGCCCGTG GTGGTAAAGT  1000
Metarhizium_brunneum_ARSEF_988           GGCTGCTTGT TTAGAGGCGC GCTGAATTAG ATGGTCTCTT AAGTGAGGGG ATTTCTCTGC TGGCAGTTGC CTGTAATCCG GGAGTCCGTG GTGGTAAAGT  1000
Metarhizium_brunneum_ART_2825            GGCTGCTTGT TTAGAGGCGC GCTGAATTAG ATGGTCTCTT AAGTGAGGGG ATTTCTCTGC TGGCAGTTGC CTGTAATCCG GGAGTCCGTG GTGGTAAAGT  1000

mpin-IDF1                                ---------- ---------- ---------- ---------- ---------- ---------- ---------- ---------- ---------- ----------  1000
mpin-IDR1                                ---------- ---------- ---------- ---------- ---------- ---------- ---------- ---------- ---------- ----------  1000
Metarhizium_pingshaense_TYPE_CBS_257.90  GGCTGCTTGT TTAGAGGCGT GCTGAATTAA ATGGTCTCTC AAGTGAGGGG ATTTCTCTGC TGGCAGTTGC TTGTGATCCG GGAGTCCGTG GCGGTAAAGT  1000
Metarhizium_pingshaense_ARSEF_2809       GGCTGCTTGT TTAGAGGCGT GCTGAATTAA ATGGTCTCTC AAGTGAGGGG ATTTCTCTGC TGGCAGTTGC TTGTGATCCG GGAGTCCGTG GCGGTAAAGT  1000
Metarhizium_pingshaense_ARSEF_3210       GGCTGCTTGT TTAGAGGCGT GCTGAATTAA ATGGTCTCTC AAGTGAGGGG ATTTCTCTGC TGGCAGTTGC TTGTGATCCG GGAGTCCGTG GCGGTAAAGT  1000
Metarhizium_pingshaense_ARSEF_4342       GGCTGCTTGT TTAGAGGCGT GCTGAATTAA ATGGTCTCTC AAGTGAGGGG ATTTCTCTGC TGGCAGTTGC TTGTGATCCG GGAGTCCGTG GCGGTAAAGT  1000
Metarhizium_pingshaense_ARSEF_7422       GGCTGCTTGT TTAAAGGCGT GCTGAATTAA ATGGTCTCTT AAGTGAGGGG ATTTCTCTGC TGGCAGTTGC TTGTGATCCG GGAGTCCGTG GCGGTAAAGT  1000
Metarhizium_pingshaense_ARSEF_7929       GGCTGCTTGT TTAGAGGCGT GCTGAATTAA ATGGTCTCTC AAGTGAGGGG ATTTCTCTGC TGGCAGTTGC TTGTGATCCG GGAGTCCGTG GCGGTAAAGT  1000

mrob-IDF1                                ---------- ---------- ---------- ---------- ---------- ---------- ---------- ---------- ---------- ----------  1000
mrob-IDR1                                ---------- ---------- ---------- ---------- ---------- ---------- ---------- ---------- ---------- ----------  1000
Metarhizium_robertsii_TYPE_ARSEF_2575    GGCTGCTTCT TTAGAGGCGT GCTGAATTAA ATGGTCTCTT GAGTGAGGGG ATTTCTCTGC TGGCAGTTGC CTGTGATCCG GGAGTCCGTG GCGGTAAAGT  1000
Metarhizium_robertsii_ARSEF_6472         GGCTGCTTCT TTAGAGGCGT GCTGAATTAA ATGGTCTCTT GAGTGAGGGG ATTTCTCTGC TGGCAGTTGC CTGTGATCCG GGAGTCCGTG GCGGTAAAGT  1000
Metarhizium_robertsii_ARSEF_727          GGCTGCTTCT TTAGAGGCGT GCTGAATTAA ATGGTCTCTT GAGTGAGGGG ATTTCTCTGC TGGCAGTTGC CTGTGATCCG GGAGTCCGTG GCGGTAAAGT  1000
Metarhizium_robertsii_ARSEF_7501         GGCTGCTTCT TTAGAGGCGT GCTGAATTAA ATGGTCTCTT GAGTGAGGGG ATTTCTCTGC TGGCAGTTGC CTGTGATCCG GGAGTCCGTG GCGGTAAAGT  1000
Metarhizium_robertsii_ARSEF_7532         GGCTGCTTCT TTAGAGGCGT GCTGAATTAA ATGGTCTCTT GAGTGAGGGG ATTTCTCTGC TGGCAGTTGC CTGTGATCCG GGAGTCCGTG GCGGTAAAGT  1000
Metarhizium_robertsii_ARSEF_8070         GGCTGCTTCT TTAGAGGCGT GCTGAATTAA ATGGTCTCTT GAGTGAGGGG ATTTCTCTGC TGGCAGTTGC CTGTGATCCG GGAGTCCGTG GCGGTAAAGT  1000
Metarhizium_robertsii_ARSEF_8685         GGCTGCTTCT TTAGAGGCGT GCTGAATTAA ATGGTCTCTT GAGTGAGGGG ATTTCTCTGC TGGCAGTTGC CTGTGATCCG GGAGTCCGTG GCGGTAAAGT  1000
```

```
mani-IDF1                           ---------- ---------- ---------- ---------- ---------- ---------- ---------- ---------- ---------- ----------  1100
mani-IDR1                           ---------- ---------- ---------- ---------- ---------- ---------- ---------- ---------- ---------- ----------  1100
Metarhizium_anisopliae_TYPE_ARSEF_7487     CAACTGTAAG GCTTGTGTGT GTG------- ---------- ---CCGGGGC CCTGTAAGTC CCGACCAGGA ACCCTACAGG TCACGTGCCG ATACAATAAG  1100
Metarhizium_anisopliae_ARSEF_1080   CAACTGTAAG GCTTGTGTGT GTG------- ---------- ---CCGGGGC CCTGTAAGTC CCGACCAGGA ACCCTACAGG TTATGTGCCG ATACAATAAG  1100
Metarhizium_anisopliae_ARSEF_6347   CAACTGTAAG GCTTGTGTGT GTG------- ---------- ---CCGGGGC CCTGTAAGTC CCGACCAGGA ACCCTACAGG TTATGTGCCG ATACAATAAG  1100
Metarhizium_anisopliae_ARSEF_7450   CAACTGTAAG GCTTGTGTGT GTG------- ---------- ---CCGGGGC CCTGTAAGTC CCGACCAGGA ACCCTACAGG TCACGTGCCG ATACAATAAG  1100
Metarhizium_anisopliae_ARSEF_798    CAACTGTAAG GCTTGTGTGT GTG------- ---------- ---CCGGGGC CCTGTAAGTC CCGACCAGGA ACCCTACAGG TTATGTGCCG ATACAATAAG  1100
Metarhizium_anisopliae_ART_2062     CAACTGTAAG GCTTGTGTGT GTG------- ---------- ---CCGGGGC CCTGTAAGTC CCGACCAGGA ACCCTACAGG TCACGTGCCG ATACAATAAG  1100

mbru-IDF1                           ---------- ---------- ---------- ---------- ---------- ---------- ---------- ---------- ---------- ----------  1100
mbru-IDR1                           ---------- ---------- ---------- ---------- ---------- ---------- ---------- ---------- ---------- ----------  1100
Metarhizium_brunneum_TYPE_ARSEF_2107  CAGCTATAAG GCTTGTGTGT GTGTGGGTGC GCGGGTGTGT GTGCCGGGGC CCTGTAAGTC CCGACCAGGA ACCCTACAGG TCACGTGCCG ACACAGTAAG  1100
Metarhizium_brunneum_ARSEF_1066     CAGCTATAAG GCTTGTGTGT GTGTGGGTGC GCGGGTGTGT GTGCCGGGGC CCTGTAAGTC CCGACCAGGA ACCCTACAGG TCACGTGCCG ACACAGTAAG  1100
Metarhizium_brunneum_ARSEF_1278     CAGCTATAAG GCTTGTGTGT GTG------- ---------- ---CCGGGGC CCTGTAAGTC CCGACCAGGA ACCCTGCAGG TCACGTGCCG ACACAGTAAG  1100
Metarhizium_brunneum_ARSEF_3295     CAGCTATAAG GCTTGTGTGT GTGTGGGTGC GCGGGTGTGT GTGCCGGGGC CCTGTAAGTC CCGACCAGGA ACCCTACAGG TCACGTGCCG ACACAGTAAG  1100
Metarhizium_brunneum_ARSEF_3826     CAGCTATAAG GCTTGTGTGT GTGTGGGTGC GCGGGTGTGT GTGCCGGGGC CCTGTAAGTC CCGACCAGGA ACCCTACAGG TCACGTGCCG ACACAGTAAG  1100
Metarhizium_brunneum_ARSEF_4152     CAGCTATAAA GCTTGTGTGT ATG------- -------TGT GTGCCGGGGC CCTGTAAGTC CCGACCAGGA ACCTTACAGG TCACGTGCCG ATACGATAAG  1100
Metarhizium_brunneum_ARSEF_4179     CAGCTATAAA GCTTGTGTGT ATG------- -------TGT GTGCCGGGGC CCTGTAAGTC CCGACCAGGA ACCTTACAGG TCACGTGCCG ATACGATAAG  1100
Metarhizium_brunneum_ARSEF_5198     CAGCTATAAG GCTTGTGTGT GTGTGGGTGC GCGGGTGTGT GTGCCGGGGC CCTGTAAGTC CCGACCAGGA ACCCTACAGG TCACGTGCCG ACACAGTAAG  1100
Metarhizium_brunneum_ARSEF_7524     CAGCTATAAG GCTTGTGTGT GTGTGGGTGC GCGGGTGTGT GTGCCGGGGC CCTGTAAGTC CCGACCAGGA ACCCTACAGG TCACGTGCCG ACACAGTAAG  1100
Metarhizium_brunneum_ARSEF_988      CAGCTATAAG GCTTGTGTGT GTGTGGGTGC GCGGGTGTGT GTGCCGGGGC CCTGTAAGTC CCGACCAGGA ACCCTACAGG TCACGTGCCG ACACAGTAAG  1100
Metarhizium_brunneum_ART_2825       CAGCTATAAG GCTTGTGTGT GTGTGGGTGC GCGGGTGTGT GTGCCGGGGC CCTGTAAGTC CCGACCAGGA ACCCTACAGG TCACGTGCCG ACACAGTAAG  1100

mpin-IDF1                           ---------- ---------- GTG------- ---------- ---CCGGGGC CCTGTAG--- ---------- ---------- ---------- ----------  1100
mpin-IDR1                           ---------- ---------- ---------- ---------- ---------- ---------- ---------- ---------- ---------- ----------  1100
Metarhizium_pingshaense_TYPE_CBS_257.90  CAACTGTAAG GCTTGTGTGT GTG------- ---------- ---CCGGGGC CCTGTAGGTC CCGACCAGGA ACCCTACAGG CCACGTGCCG ATATAATAAG  1100
Metarhizium_pingshaense_ARSEF_2809  CAACTGTAAG GCTTGTGTGT GTG------- ---------- ---CCGGGGC CCTGTAGGTC CCGACCAGGA ACCCCACAGG TCACGTGCC- ----------  1100
Metarhizium_pingshaense_ARSEF_3210  CAACTGTAAG GCTTGTGTGT GTG------- ---------- ---CCGGGGC CCTGTAGGTC CCGACCAGGA ACCCTACAGG TCACGTGCCG ATACAATAAG  1100
Metarhizium_pingshaense_ARSEF_4342  CAACTGTAAG GCTTGTGTGT GTG------- ---------- ---CCGGGGC CCTGTAGGTC CCGACCAGGA ACCCTACAGG TCACGTGCCG ATATAATAAG  1100
Metarhizium_pingshaense_ARSEF_7422  CAACTGTAAG GCTTGCGTGT GTG------- ---------- ---CCGGGGC CCTGTAGGTC CCGACCAGGA ACCCTACAGG TCACGTGCCG ATATAATAAG  1100
Metarhizium_pingshaense_ARSEF_7929  CAACTGTAAG GCTTGTGTGT GTG------- ---------- ---CCGGGGC CCTGTAGGTC CCGACCAGGA ACCCTACAGG TCACGTGCCG ATACAATAAG  1100

mrob-IDF1                           ---------- ---------- ---------- ---------- ---------- ---------- ---------- ---------- ---------- ----------  1100
mrob-IDR1                           ---------- ---------- ---------- ---------- ---------- ---------- ---------- ---------- ---------- ----------  1100
Metarhizium_robertsii_TYPE_ARSEF_2575  CAACTGTAAG GCTTGTGTGT GTG------- ---------- ---CCGGGGC CCTGTAAGTC CCGACCAGGA ACCCTACAGG TCACGTGCCG ATACAATAAG  1100
Metarhizium_robertsii_ARSEF_6472    CAACTGTAAG GCTTGTGTGT GTG------- ---------- ---CCGGGGC CCTGTAAGTC CCGACCAGGA ACCCTACAGG TCACGTGCCG ATACAATAAG  1100
Metarhizium_robertsii_ARSEF_727     CAACTGTAAG GCTTGTGTGT GTG------- ---------- ---CCGGGGC CCTGTAAGTC CCGACCAGGA ACCCTACAGG TCACGTGCCG ATACAATAAG  1100
Metarhizium_robertsii_ARSEF_7501    CAACTGTAAG GCTTGTGTGT GTG------- ---------- ---CCGGGGC CCTGTAAGTC CCGACCAGGA ACCCTACAGG TCACGTGCCG ATACAATAAG  1100
Metarhizium_robertsii_ARSEF_7532    CAACTGTAAG GCTTGTGTGT GTG------- ---------- ---CCGGGGC CCTGTAAGTC CCGACCAGGA ACCCTACAGG TCACGTGCCG ATACAATAAG  1100
Metarhizium_robertsii_ARSEF_8070    CAACTGTAAG GCTTGTGTGT GTG------- ---------- ---CCGGGGC CCTGTAAGTC CCGACCAGGA ACCCTACAGG TCACGTGCCG ATACAATAAG  1100
Metarhizium_robertsii_ARSEF_8685    CAACTGTAAG GCTTGTGTGT GTG------- ---------- ---CCGGGGC CCTGTAAGTC CCGACCAGGA ACCCTACAGG TCACGTGCCG ATACAATAAG  1100
```

```
mani-IDF1                             ---------- ---------- ---------- ---------- ---------- ---------- ---------- ---------- ---------- ----------  1200
mani-IDR1                             ---------- ---------- ---------- ---------- ---------- ---------- ---------- ---------- ---------- ----------  1200
Metarhizium_anisopliae_TYPE_ARSEF_7487     ACAGATAAGA TATGCGGCAC GTGCTAGTAC ACGTGCCAAT ACAAGT---- -GACGATAAG ATCGATAAGA TACCTGCACG TGCCTAGTCA CGGAGACTTA  1200
Metarhizium_anisopliae_ARSEF_1080     ACAGATAAGA TATGCGGCAC GTGCTAGTAC ACGTGCCAAT ACAAGT---- -GACGATAAG ATCGATAAGA TACCTGCACG TGCCTAGTCA CGGAGACTTA  1200
Metarhizium_anisopliae_ARSEF_6347     ACAGATAAGA TATGCGGCAC GTGCTAGTAC ACGTGCCAAT ACAAGT---- -GACGATAAG ATCGATAAGA TACCTGCACG TGCCTAGTCA CGGAGACTTA  1200
Metarhizium_anisopliae_ARSEF_7450     ACAGATAAGA TATGCGGCAC GTGCTAGTAC ACGTGCCAAT ACAAGT---- -GACGATAAG ATCGATAAGA TACCTGCACG TGCCTAGTCA CGGAGACTTA  1200
Metarhizium_anisopliae_ARSEF_798      ACAGATAAGA TATGCGGCAC GTGCTAGTAC ACGTGCCAAT ACAAGT---- -GACGATAAG ATCGATAAGA TACCTGCACG TGCCTAGTCA CGGAGACTTA  1200
Metarhizium_anisopliae_ART_2062       ACAGATAAGA TATGCGGCAC GTGCTAGTAC ACGTGCCAAT ACAAGT---- -GACGATAAG ATCGATAAGA TACCTGCACG TGCCTAGTCA CGGAGACTTA  1200

mbru-IDF1                             ---------- ---------- ---------- ---------- ---------- ---------- ---------- ---------- ---------- ----------  1200
mbru-IDR1                             ---------- ---------- ---------- ---------- ---------- ---------- ---------- ---------- ---------- ----------  1200
Metarhizium_brunneum_TYPE_ARSEF_2107  ACAGATAAGA TATACGGCAC GTGTTAGTAC ACGTGCCAAT ACAAGT---- -GACGATAAG ATCGATAAGA TACCTGCACG TGCCCAGTCA CGGAGACTTA  1200
Metarhizium_brunneum_ARSEF_1066       ACAGATAAGA TATACGGCAG GTGTTAGTAC ACGTGCCAAT ACAAGT---- -GACGATAAG ATCGATAAGA TACCTGCACG TGCCCAGTCA CGGAGACTTA  1200
Metarhizium_brunneum_ARSEF_1278       ACAGATAAGA TATACGGCAC GTGTTAGTAC ACGTGCCAAT ACAAGT---- -GACGATAAG ATCGATAAGA TACCTGCACG TGCCCAGTCA CGGAGACTTA  1200
Metarhizium_brunneum_ARSEF_3295       ACAGATAAGA TATACGGCAC GTGTTAGTAC ACGTGCCAAT ACAAGT---- -GACGATAAG ATCGATAAGA TACCTGCAC- ---------- ----------  1200
Metarhizium_brunneum_ARSEF_3826       ACAGATAAGA TATACGGCAG GTGTTAGTAC ACGTGCCAAT ACAAGT---- -GACGATAAG ATCGATAAGA TACCTGCACG TGCCCAGTCA CGGAGACTTA  1200
Metarhizium_brunneum_ARSEF_4152       ACAGATAAGA TATACAGCAC GTGCTAGTAC ACGTGCCAGT ATAAGTAACG AGACGATAAG ATCGATAAGA TACCTGCACG TGCCTAGTCA CGGAGACTTA  1200
Metarhizium_brunneum_ARSEF_4179       ACAGATAAGA TATACAGCAC GTGCTAGTAC ACGTGCCAGT ATAAGTAACG AGACGATAAG ATCGATAAGA TACCTGCACG TGCCTAGTCA CGGAGACTTA  1200
Metarhizium_brunneum_ARSEF_5198       ACAGATAAGA TATACGGCAG GTGTTAGTAC ACGTGCCAAT ACAAGT---- -GACGATAAG ATCGATAAGA TACCTGCACG TGCCCAGTCA CGGAGACTTA  1200
Metarhizium_brunneum_ARSEF_7524       ACAGATAAGA TATACGGCAG GTGTTAGTAC ACGTGCCAAT ACAAGT---- -GACGATAAG ATCGATAAGA TACCTGCACG TGCCCAGTCA CGGAGACTTA  1200
Metarhizium_brunneum_ARSEF_988        ACAGATAAGA TATACGGCAC GTGTTAGTAC ACGTGCCAAT ACAAGT---- -GACGATAAG ATCGATAAGA TACCTGCAC- ---------- ----------  1200
Metarhizium_brunneum_ART_2825         ACAGATAAGA TATACGGCAC GTGTTAGTAC ACGTGCCAAT ACAAGTAA-G TGACGATAAG ATCGATAAGA TACCTGCAC- ---------- ----------  1200

mpin-IDF1                             ---------- ---------- ---------- ---------- ---------- ---------- ---------- ---------- ---------- ----------  1200
mpin-IDR1                             ---------- ---------- ---------- ---------- ---------- ---------- ---------- ---------- ---------- ----------  1200
Metarhizium_pingshaense_TYPE_CBS_257.90    ATAGATAAGG TATACGGCAC GTGCTAATAC ACGTGCCAAT ACAAGT---- -AACGATAAG ATCAACAAGA TACCTGCACG TGCCTAGTCA CGGAGACTTA  1200
Metarhizium_pingshaense_ARSEF_2809    ---------- ---------- -----AATAC ACGTGCCAAT ATAAGT---- -AACGATAAG ATCAACAAGA TACCTGCACG TGCCTAGTCA CGGAGACTTA  1200
Metarhizium_pingshaense_ARSEF_3210    ATAGATAAGG TATACGGCAC GTGCTAATAC ACGTGCCAAT ACAAGT---- -AACGATAAG ATCAACAAGA TACCTGCACG TGCCTAGTCA CGGAGACTTA  1200
Metarhizium_pingshaense_ARSEF_4342    ACAGATAAGA TATACGGCAC GTGCTAATAC ACGTGCCAAT ACAAGT---- -AACGATAAG ATCAACAAGA TACCTGCACG TGCCTAGTCA CGGAGACTTA  1200
Metarhizium_pingshaense_ARSEF_7422    ACAGATAAGA TATACGGCAC GTGCTAATAC ACGTGCCAAT ACAAGT---- -AACGATAAG ATCAACAAGA TACCTGCACG TGCCTAGTCA CGGAGACTTA  1200
Metarhizium_pingshaense_ARSEF_7929    ATAGATAAGG TATACGGCAC GTGCTAATAC ACGTGCCAAT ACAAGT---- -AACGATAAG ATCAACAAGA TACCTGCACG TGCCTAGTCA CGGAGACTTA  1200

mrob-IDF1                             ---------- ---------- ---------- ---------- ---------- ---------- ---------- ---------- ---------- ----------  1200
mrob-IDR1                             ---------- ---------- ---------- ---------- ---------- ---------- ---------- ---------- ---------- ----------  1200
Metarhizium_robertsii_TYPE_ARSEF_2575 ACAGATAAGA TATACGGCAC GTGCTAATAC ACGTGCCAAT ACAAGT---- -GACGATAAG ATCAACAAGA TACCTGCACG TGCCTAGTCA CGGAGACTTA  1200
Metarhizium_robertsii_ARSEF_6472      ACAGATAAGA TATACGGCAC GTGCTAATAC ACGTGCCAAT ACAAGT---- -GACGATAAG ATCAACAAGA TACCTGCACG TGCCTAGTCA CGGAGACTTA  1200
Metarhizium_robertsii_ARSEF_727       ACAGATAAGA TATACGGCAC GTGCTAATAC ACGTGCCAAT ACAAGT---- -GACGATAAG ATCAACAAGA TACCTGCACG TGCCTAGTCA CGGAGACTTA  1200
Metarhizium_robertsii_ARSEF_7501      ACAGATAAGA TATACGGCAC GTGCTAATAC ACGTGCCAAT ACAAGT---- -GACGATAAG ATCAACAAGA TACCTGCACG TGCCTAGTCA CGGAGACTTA  1200
Metarhizium_robertsii_ARSEF_7532      ACAGATAAGA TATACGGCAC GTGCTAATAC ACGTGCCAAT ACAAGT---- -GACGATAAG ATCAACAAGA TACCTGCACG TGCCTAGTCA CGGAGACTTA  1200
Metarhizium_robertsii_ARSEF_8070      ACAGATAAGA TATACGGCAC GTGCTAATAC ACGTGCCAAT ACAAGT---- -GACGATAAG ATCAACAAGA TACCTGCACG TGCCTAGTCA CGGAGACTTA  1200
Metarhizium_robertsii_ARSEF_8685      ACAGATAAGA TATACGGCAC GTGCTAATAC ACGTGCCAAT ACAAGT---- -GACGATAAG ATCAACAAGA TACCTGCACG TGCCTAGTCA CGGAGACTTA  1200
```

```
mani-IDF1                              ---------- ---------- ---------- ---------- ---------- ---------- ---------- ---------- ---------- ----------  1300
mani-IDR1                              ---------- ---------- ---------- ---------- ---------- ---------- ---------- ---------- ---------- ----------  1300
Metarhizium_anisopliae_TYPE_ARSEF_7487 CACGTGTTCC GCTGCCAAGG TCCCCGAGGT CCCCGCGATT GCCGACCTCG CCGACTACTC CTGGTGTAAT ATACCGGTGT AGTTGTATAT AATTTCTTAC  1300
Metarhizium_anisopliae_ARSEF_1080      CACGTGTTCC GCTGCCAAGG TCCCCGAGGT CCCCGCGATT GCCGACCTCG CCGACTACTC CTGGTGTAAT ATGCCGGTGT AGTTGTGTAT AATTTCTTAC  1300
Metarhizium_anisopliae_ARSEF_6347      CACGTGTTCC GCTGCCAAGG TCCCCGAGGT CCCCGCGATT GCCGACCTCG CCGACTACTC CTGGTGTAAT ATGCCGGTGT AGTTGTGTAT AATTTCTTAC  1300
Metarhizium_anisopliae_ARSEF_7450      CACGTGTTCC GCTGCCAAGG TCCCCGAGGT CCCCGCGATT GCCGACCTCG CCGACTACTC CTGGTGTAAT ATACCGGTGT AGTTGTATAT AATTTCTTAC  1300
Metarhizium_anisopliae_ARSEF_798       CACGTGTTCC GCTGCCAAGG TCCCCGAGGT CCCCGCGATT GCCGACCTCG CCGACTACAC CTGGTGTAAT ATGCCGGTGT AGTTGTGTAT AATTTCTTAC  1300
Metarhizium_anisopliae_ART_2062        CACGTGTTCC GCTGCCAAGG TCCCCGAGGT CCCCGCGATT GCCGACCTCG CCGACTACTC CTGGTGTAAT ATACCGGTGT AGTTGTATAT AATTTCTTAC  1300

mbru-IDF1                              ---------- ---------- ---------- ---------- ---------- ---------- ---------- ---------- ---------- ----------  1300
mbru-IDR1                              ---------- ---------- ---------- ---------- ---------- ---------- ---------- ---------- ---------- ----------  1300
Metarhizium_brunneum_TYPE_ARSEF_2107   CACGTGTCCC GCTGCCAAGG TCCCCGAGGT CCCCGAGGTT GCCGACCTCG CCGACTACTC CTGGTGTAAT GTGCCGGTGC AGTTGCATAT AATTCCTTAC  1300
Metarhizium_brunneum_ARSEF_1066        CACGTGTCCC GCTGCCAAGG TCCCCGAGGT ---------T GCCGACCTCG CCGACTACTC CTGGTGTAAT GTGCCGGTGC A--------- ----------  1300
Metarhizium_brunneum_ARSEF_1278        CACGTGTCCC GCTGCCAAGG TCCCCGAGGT CCCCGAGGTT GCCGACCTCG CCGACTACTC CTGGTGTAAT GTGCCGGTGC AGTTGCATAT AATTCCTTAC  1300
Metarhizium_brunneum_ARSEF_3295        ---------- ---------- ---------- ---------- ---------- ---------- ---------- ---------- ---------- ----------  1300
Metarhizium_brunneum_ARSEF_3826        CACGTGTCCC GCTGCCAAGG TCCCCGAGGT ---------T GCCGACCTCG CCGACTACTC CTGGTGTAAT GTGCCGGTGC A--------- ----------  1300
Metarhizium_brunneum_ARSEF_4152        CACGTGTCCC GCCGCCAAGG TCCCCAAGGT CCCCGAGGTT GCCGACCTCG CCGACTACTC CTGGTGTAAT ATGCCGGTAC AGTTGCATAT AATTCCCTAC  1300
Metarhizium_brunneum_ARSEF_4179        CACGTGTCCC GCCGCCAAGG TCCCCAAGGT CCCCGAGGTT GCCGACCTCG CCGACTACTC CTGGTGTAAT ATGCCGGTAC AGTTGCATAT AATTCCCTAC  1300
Metarhizium_brunneum_ARSEF_5198        CACGTGTCCC GCTGCCAAGG TCCCCGAGGT ---------T GCCGACCTCG CCGACTACTC CTGGTGTAAT GTGCCGGTGC A--------- ----------  1300
Metarhizium_brunneum_ARSEF_7524        CACGTGTCCC GCTGCCAAGG TCCCCGAGGT ---------T GCCGACCTCG CCGACTACTC CTGGTGTAAT GTGCCGGTGC A--------- ----------  1300
Metarhizium_brunneum_ARSEF_988         ---------- ---------- ---------- ---------- ---------- ---------- ---------- ---------- ---------- ----------  1300
Metarhizium_brunneum_ART_2825          ---------- ---------- ---------- ---------- ---------- ---------- ---------- ---------- ---------- ----------  1300

mpin-IDF1                              ---------- ---------- ---------- ---------- ---------- ---------- ---------- ---------- ---------- ----------  1300
mpin-IDR1                              ---------- ---------- ---------- ---------- ---------- ---------- ---------- ---------- ---------- ----------  1300
Metarhizium_pingshaense_TYPE_CBS_257.90 CACGTGTCCC GCTGCCAAGA TCCCCGAGGT ---------T GCCGACCTCG CCGACTACTC CTGGTGTAAT GTGCCGGTGT AGTTATATAT AATTTCTTAC  1300
Metarhizium_pingshaense_ARSEF_2809     CACGTGTCCC GCTGCCAAGA TCCCCGAGGT ---------T GCCGACCTCG CCGACCACTC CTGGTGTAAT GTGCCGGTGT AGTTATATAT AATTTCTTAC  1300
Metarhizium_pingshaense_ARSEF_3210     CACGTGTCCC GCTGCCAAGA TCCCCGAGGT ---------T GCCGACCTCG CCGACTACTC CTGGTGTAAT GTGCCGGTGT AGTTATATAT AATTTCTTAC  1300
Metarhizium_pingshaense_ARSEF_4342     CACGTGTCCC GCTGCCAAGA TCCCCGAGAT ---------T GCCGACCTCG CCGACTACTC CTGGTGTAAT GTGCCGGTGT AGTTATATAT AATTTCTTAC  1300
Metarhizium_pingshaense_ARSEF_7422     CACGTGTCCC GCTGCCAAGA TCCCCGAGGT ---------T GCCGACCTCG CCGACTACTC CTGGCGTAAT GTGCCGGTGT AGTTATATAT AATTTCTTAC  1300
Metarhizium_pingshaense_ARSEF_7929     CACGTGTCCC GCTGCCAAGA TCCCCGAGGT ---------T GCCGACCTCG CCGACTACTC CTGGTGTAAT GTGCCGGTGT AGTTATATAT AATTTCTTAC  1300

mrob-IDF1                              ---------- ---------- ---------- ---------- ---------- ---------- ---------- ---------- ---------- ----------  1300
mrob-IDR1                              ---------- ---------- ---------- ---------- ---------- ---------- ---------- ---------- ---------- ----------  1300
Metarhizium_robertsii_TYPE_ARSEF_2575  CACGTGTCCC GCTGCCAAGA TCCCCGAGAT ---------T GCCGACCTCG CCGACTACTC TTGGTGTAAT GTGCCGGTGT ---------- AATTTCTTGC  1300
Metarhizium_robertsii_ARSEF_6472       CACGTGTCCC GCTGCCAAGA TCCCCGAGAT ---------T GCCGACCTCG CCGACTACTC TTGGTGTAAT GTGCCGGTGT ---------- AATTTCTTAC  1300
Metarhizium_robertsii_ARSEF_727        CACGTGTCCC GCTGCCAAGA TCCCCGAGAT ---------T GCCGACCTCG CCGACTACTC TTGGTGTAAT GTGCCGGTGT ---------- AATTTCTTAC  1300
Metarhizium_robertsii_ARSEF_7501       CACGTGTCCC GCTGCCAAGA TCCCCGAGAT ---------T GCCGACCTCG CCGACTACTC TTGGTGTAAT GTGC------ ---------- ----------  1300
Metarhizium_robertsii_ARSEF_7532       CACGTGTCCC GCTGCCAAGA TCCCCGAGAT ---------T GCCGACCTCG CCGACTACTC TTGGTGTAAT GTGCCGGTGT ---------- AATTTCTTAC  1300
Metarhizium_robertsii_ARSEF_8070       CACGTGTCCC GCTGCCAAGA TCCCCGAGAT ---------T GCCGACCTCG CCGACTACTC TTGGTGTAAT GTGCCGGTGT ---------- AATTTCTTAC  1300
Metarhizium_robertsii_ARSEF_8685       CACGTGTCCC GCTGCCAAGA TCCCCGAGAT ---------T GCCGACCTCG CCGACTACTC TTGGTGTAAT GTGCCGGTGT ---------- AATTTCTTAC  1300
```

```
mani-IDF1                              ---------- ---------- ---------- ---------- ---------- ---------- ---------- ---------- ---------- ----------  1400
mani-IDR1                              ---------- ---------- ---------- ---------- ---------- ---------- ---------- ---------- ---------- ----------  1400
Metarhizium_anisopliae_TYPE_ARSEF_7487 TAGTCTTAAT AGTAGTACAC TTGTTTATCG CGATACACTT ACTTGTGGTG CGGCTGTGCG GCTGTGCGGC TGTGCTGACG TACTTGTAGT GGTGCACTTA  1400
Metarhizium_anisopliae_ARSEF_1080      TAGTCTTAAT AGTAGTACAC TTGTTTATCG CGATACACTT ACTTGTG--- -----GTGCG GCTGTGCGGC TGTGCTGATG TACTTGTAGT GGTGCACTTA  1400
Metarhizium_anisopliae_ARSEF_6347      TAGTCTTAAT AGTAGTACAC TTGTTTATCG CGATACACTT ACTTGTG--- -----GTGCG GCTGTGCGGC TGTGCTGATG TACTTGTAGT GGTGCACTTA  1400
Metarhizium_anisopliae_ARSEF_7450      TAGTCTTAAT AGTAGTACAC TTGTTTATCG CGATACACTT ACTTGTGGTG CGGCTGTGCG GCTGTGCGGC TGTGCTGACG TACTTGTAGT GGTGCACTTA  1400
Metarhizium_anisopliae_ARSEF_798       TAGTCTTAAT AGTAGTACAC TTGTTTATCG CGATACACTT ACTTGTG--- -----GTGCG GCTGTGCGGC TGTGCTGATG TACTTGTAGT GGTGCACTTA  1400
Metarhizium_anisopliae_ART_2062        TAGTCTTAAT AGTAGTACAC TTGTTTATCG CGATACACTT ACTTGTGGTG CGGCTGTGCG GCTGTGCGGC TGTGCTGACG TACTTGTAGT GGTGCACTTA  1400

mbru-IDF1                              ---------- ---------- ---------- ---------- ---------- ---------- ---------- ---------- ---------- ----------  1400
mbru-IDR1                              ---------- ---------- ---------- ---------- ---------- ---------- ---------- ---------- ---------- ----------  1400
Metarhizium_brunneum_TYPE_ARSEF_2107   TAGTCTCAAT AATAGTACAC TCGTTTATCG CGATGCACTC ACTTGTG--- -----GTACG GCTGTGCGGC TGTGCTGACG TACTTGTGGT ----------  1400
Metarhizium_brunneum_ARSEF_1066        ---------- ---------- ---------- ---------- ---------- ---------- ---------- ---------- ---------- ----------  1400
Metarhizium_brunneum_ARSEF_1278        TAGTCTCAAT AATAGTACAC TCGTTTATCG CGATGCACTC ACTTGTG--- -----GTACG GCTGTGCGGC TGTGCTGACG TACTT----- ----------  1400
Metarhizium_brunneum_ARSEF_3295        ---------- ---------- ---------- ---------- ---------- ---------- ---------- ---------- ---------- ----------  1400
Metarhizium_brunneum_ARSEF_3826        ---------- ---------- ---------- ---------- ---------- ---------- ---------- ---------- ---------- ----------  1400
Metarhizium_brunneum_ARSEF_4152        TAGTCTCAAT AATAGTACAC TCGTTTATCG CGATGCACTT ACTTGTG--- -----GTGCG GCTGTGCGGC TGTGCTGACG TACTTGTGGT AGTGCACTTA  1400
Metarhizium_brunneum_ARSEF_4179        TAGTCTCAAT AATAGTACAC TCGTTTATCG CGATGCACTT ACTTGTG--- -----GTGCG GCTGTGCGGC TGTGCTGACG TACTTGTGGT AGTGCACTTA  1400
Metarhizium_brunneum_ARSEF_5198        ---------- ---------- ---------- ---------- ---------- ---------- ---------- ---------- ---------- ----------  1400
Metarhizium_brunneum_ARSEF_7524        ---------- ---------- ---------- ---------- ---------- ---------- ---------- ---------- ---------- ----------  1400
Metarhizium_brunneum_ARSEF_988         ---------- ---------- ---------- ---------- ---------- ---------- ---------- ---------- ---------- ----------  1400
Metarhizium_brunneum_ART_2825          ---------- ---------- ---------- ---------- ---------- ---------- ---------- ---------- ---------- ----------  1400

mpin-IDF1                              ---------- ---------- ---------- ---------- ---------- ---------- ---------- ---------- ---------- ----------  1400
mpin-IDR1                              ---------- ---------- ---------- ---------- ---------- ---------- ---------- ---------- ---------- ----------  1400
Metarhizium_pingshaense_TYPE_CBS_257.90 TAGTCTCAAT AATAGTACAC TCGTTTATCG CGATGCACTC ACTTGTG--- -----GTGCG GGTGTGCGGG TGTGCTGACG TACTTGTGGT GGTGCACTTA  1400
Metarhizium_pingshaense_ARSEF_2809     TAGTCTCAAT AATAGTACAC TC-------- ---------- ACTTGTG--- -----GTGCG GCTGTGCGGC TGTGCTGACG TGCTTGTGGT GGTGCACTTA  1400
Metarhizium_pingshaense_ARSEF_3210     TAGTCTCAAT AATAGTACAC TCGTTTATCG CGATGCACTC ACTTGTG--- -----GTGCG GGTGTGCGGG TGTGCTGACG TACTTGTGGT GGTGCACTTA  1400
Metarhizium_pingshaense_ARSEF_4342     TAGTCTCAAT AATAGTACAC TCGTTTATCG CGATGCACTC ACTTGTG--- ---------- ---------- ---------- --------GT GGTGCACTTA  1400
Metarhizium_pingshaense_ARSEF_7422     TAGTCTCAAT AATAGTACAC CCGTTTATCG CGATGCACTC ACTTGTG--- ---------- ---------- ---------- --------GT GGTGCACTTA  1400
Metarhizium_pingshaense_ARSEF_7929     TAGTCTCAAT AATAGTACAC TCGTTTATCG CGATGCACTC ACTTGTG--- -----GTGCG GGTGTGCGGG TGTGCTGACG TACTTGTGGT GGTGCACTTA  1400

mrob-IDF1                              ---------- ---------- ---------- ---------- ---------- ---------- ---------- ---------- ---------- ----------  1400
mrob-IDR1                              ---------- ---------- ---------- ---------- ---------- ---------- ---------- ---------- ---------- ----------  1400
Metarhizium_robertsii_TYPE_ARSEF_2575  TAGTCTCAAT AATAGTACAC TCGTTTATCG CGATGCACTT ACTTGTA--- ---------- ---GTGCGGT TGTGCTGACG TACTTGTGGT GGTGTACTTA  1400
Metarhizium_robertsii_ARSEF_6472       TAGTCTCAAT AATAGTACAC TCGTTTATCG CGATGCACTT ACTTGTA--- ---------- ---GTGCGGT TGTGCTGACG TACTTGTGGT GGTGTACTTA  1400
Metarhizium_robertsii_ARSEF_727        TAGTCTCAAT AATAGTACAC TCGTTTATCG CGATGCACTT ACTTGTA--- ---------- ---GTGCGGT TGTGCTGACG TACTTGTGGT GGTGTACTTA  1400
Metarhizium_robertsii_ARSEF_7501       ---------- ---------- ---------- ---------- ---------- ---------- ---------- ---------- ---------- ----------  1400
Metarhizium_robertsii_ARSEF_7532       TAGTCTCAAT AATAGTACAC TCGTTTATCG CGATGCACTT ACTTGTA--- ---------- ---GTGCGGT TGTGCTGACG TACTTGTGGT GGTGTACTTA  1400
Metarhizium_robertsii_ARSEF_8070       TAGTCTCAAT AATAGTACAC TCGTTTATCG CGATGCACTT ACTTGTA--- ---------- ---GTGCGGT TGTGCTGACG TACTTGTGGT GGTGTACTTA  1400
Metarhizium_robertsii_ARSEF_8685       TAGTCTCAAT AATAGTACAC TCGTTTATCG CGATGCACTT ACTTGTA--- ---------- ---GTGCGGT TGTGCTGACG TACTTGTGGT GGTGTACTTA  1400
```

```
mani-IDF1                              ---------- ---------- ---------- ---------- ---------- ---------- ---------- ---------- ---------- ----------  1500
mani-IDR1                              ---------- ---------- ---------- ---------- ---------- ---------- ---------- ---------- ---------- ----------  1500
Metarhizium_anisopliae_TYPE_ARSEF_7487 CTCTACAATA GTGCTGGTAC TTACGGTCGC ACACAAATCC CTAGTATTTT GGTGTAGTTG TGATCGTGGT GGTGCAGTTA CAGCAGTGCA CTTACCCTGT  1500
Metarhizium_anisopliae_ARSEF_1080      CTCTACAATA GTGCTGGTAC TTACGGTCGC ACACAAATCC CTAGTATTTT GGTGTAGTTG TGGTCGTGGT GGTGCAGTTA CACCAGTGCA CTTACCCTGT  1500
Metarhizium_anisopliae_ARSEF_6347      CTCTACAATA GTGCTGGTAC TTACGGTCGC ACACAAATCC CTAGTATTTT GGTGTAGTTG TGGTCGTGGT GGTGCAGTTA CAGCAGTGCA CTTACCCTGT  1500
Metarhizium_anisopliae_ARSEF_7450      CTCTACAATA GTGCTGGTAC TTACGGTCGC ACACAAATCC CTAGTATTTT GGTGTAGTTG TGATCGTGGT GGTGCAGTTA CAGCAGTGCA CTTACCCTGT  1500
Metarhizium_anisopliae_ARSEF_798       CTCTACAATA GTGCTGGTAC TTACGGTCGC ACACAAATCC CTAGTATTTT GGTGTAGTTG TGGTCGTGGT GGTGCAGTTA CAGCAGTGCA CTTACCCTGT  1500
Metarhizium_anisopliae_ART_2062        CTCTACAATA GTGCTGGTAC TTACGGTCGC ACACAAATCC CTAGTATTTT GGTGTAGTTG TGATCGTGGT GGTGCAGTTA CAGCAGTGCA CTTACCCTGT  1500

mbru-IDF1                              ---------- ---------- ---------- ---------- ---------- ---------- ---------- ---------- ---------- ----------  1500
mbru-IDR1                              ---------- ---------- ---------- ---------- ---------- ---------- ---------- ---------- ---------- ----------  1500
Metarhizium_brunneum_TYPE_ARSEF_2107   ---------- ---------- ---------- ---------- ---------- ---------- ---------- ---------- ---------- ----------  1500
Metarhizium_brunneum_ARSEF_1066        ---------- ---------- ---------- ---------- ---------- ---------- ---------- ---------- ---------- ----------  1500
Metarhizium_brunneum_ARSEF_1278        ---------- ---------- ---------- ---------- ---------- ---------- -----GTGGT GGTGGAGTTA CAGCAGTGCA CTTACCCTGT  1500
Metarhizium_brunneum_ARSEF_3295        ---------- ---------- ---------- ---------- ---------- ---------- ---------- ---------- ---------- -TTACCCTGT  1500
Metarhizium_brunneum_ARSEF_3826        ---------- ---------- ---------- ---------- ---------- ---------- ---------- ---------- ---------- ----------  1500
Metarhizium_brunneum_ARSEF_4152        CTCTACAATA GTGCTGGTAC TTGCGGTCAC ATACAATTTC CCAGTATTTT GGCGTAGTTG TGGTCGTGGT GGTGCAGTTA CAGCAGTGCA CTTACCCTGT  1500
Metarhizium_brunneum_ARSEF_4179        CTCTACAATA GTGCTGGTAC TTGCGGTCAC ATACAATTTC CCAGTATTTT GGCGTAGTTG TGGTCGTGGT GGTGCAGTTA CAGCAGTGCA CTTACCCTGT  1500
Metarhizium_brunneum_ARSEF_5198        ---------- ---------- ---------- ---------- ---------- ---------- ---------- ---------- ---------- ----------  1500
Metarhizium_brunneum_ARSEF_7524        ---------- ---------- ---------- ---------- ---------- ---------- ---------- ---------- ---------- ----------  1500
Metarhizium_brunneum_ARSEF_988         ---------- ---------- ---------- ---------- ---------- ---------- ---------- ---------- ---------- -TTACCCTGT  1500
Metarhizium_brunneum_ART_2825          ---------- ---------- ---------- ---------- ---------- ---------- ---------- ---------- ---------- -TTACCCTGT  1500

mpin-IDF1                              ---------- ---------- ---------- ---------- ---------- ---------- ---------- ---------- ---------- ----------  1500
mpin-IDR1                              ---------- ---------- ---------T ATACAAGTTC CTAGTATTTT GGC------- ---------- ---------- ---------- ----------  1500
Metarhizium_pingshaense_TYPE_CBS_257.90 CTCTACAATA GTGCTGGTAC TTGCGGTCAT ATACAAGTTC CTAGTATTTT GGCGTAGTTG TGGCCGTGGT CGTGCAGTTA CAGCAGTGTA CTTACCCTGT  1500
Metarhizium_pingshaense_ARSEF_2809     CTCTACAATA GTGCTGGTAC TTGCGGTCAT ATACAAGTTC CTAGTATTTT GGCGTAGTTG TGGTCGTGGT CGTGCAGTTA CAGCAGTGTA CTTACCCTGT  1500
Metarhizium_pingshaense_ARSEF_3210     CTCTACAATA GTGCTGGTAC TTGCGGTCAT ATACAAGTTC CTAGTATTTT GGCGTAGTTG TGGCCGTGGT CGTGCAGTTA CAGCAGTGTA CTTACCCTGT  1500
Metarhizium_pingshaense_ARSEF_4342     CTCTACAATA GTGCTGGTAC TTGCGGTCAT ATACAAGTTC CTAGTATTTT GGCGTAGTTG TGGTCGTGGT CGTGCAGTTA CAGCAGTGTA CTTACCCTGT  1500
Metarhizium_pingshaense_ARSEF_7422     CTCTACAATA GTGCTGGTAC TTGCGGTCAT ATACAAGTTC CTAGTATTTT GGCGTAGTTG TGGTCGTGGT CGTGCAGTTA CAGCAGTGTA CTTACCCTGT  1500
Metarhizium_pingshaense_ARSEF_7929     CTCTACAATA GTGCTGGTAC TTGCGGTCAT ATACAAGTTC CTAGTATTTT GGCGTAGTTG TGGCCGTGGT CGTGCAGTTA CAGCAGTGTA CTTACCCTGT  1500

mrob-IDF1                              ---------- ---------- ---------- ---------- ---------- ---------- ---------- ---------- ---------- ----------  1500
mrob-IDR1                              ---------- ---------- ---------- ---------- ---------- ---------- ---------- ---------- ---------- ----------  1500
Metarhizium_robertsii_TYPE_ARSEF_2575  CTCTATAATA GTGCTGGTAC TTGCGGTCAC ATACAAGTTC CTAGTATTTT GGCGTAGTTG TGGTCGTGGT CGTGCAGTTA CAGCAGTGCA CTTACCCTGT  1500
Metarhizium_robertsii_ARSEF_6472       CTCTATAATA GTGCTGGTAC TTGCGGTCAC ATACAAGTTC CTAGTATTTT GGCGTAGTTG TGGTCGTGGT CGTGCAGTTA CAGCAGTGCA CTTACCCTGT  1500
Metarhizium_robertsii_ARSEF_727        CTCTATAATA GTGCTGGTAC TTGCGGTCAC ATACAAGTTC CTAGTATTTT GGCGTAGTTG TGGTCGTGGT CGTGCAGTTA CAGCAGTGCA CTTACCCTGT  1500
Metarhizium_robertsii_ARSEF_7501       ---------- ----TGGTAC TTGCGGTCAC ATACAAGTTC CTAGTATTTT GGCGTAGTTG TGGTCGTGGT CGTGCAGTTA CAGCAGTGCA CTTACCCTGT  1500
Metarhizium_robertsii_ARSEF_7532       CTCTATAATA GTGCTGGTAC TTGCGGTCAC ATACAAGTTC CTAGTATTTT GGCGTAGTTG TGGTCGTGGT CGTGCAGTTA CAGCAGTGCA CTTACCCTGT  1500
Metarhizium_robertsii_ARSEF_8070       CTCTATAATA GTGCTGGTAC TTGCGGTCAC ATACAAGTTC CTAGTATTTT GGCGTAGTTG TGGTCGTGGT CGTGCAGTTA CAGCAGTGCA CTTACCCTGT  1500
Metarhizium_robertsii_ARSEF_8685       CTCTATAATA GTGCTGGTAC TTGCGGTCAC ATACAAGTTC CTAGTATTTT GGCGTAGTTG TGGTCGTGGT CGTGCAGTTA CAGCAGTGCA CTTACCCTGT  1500
```

```
mani-IDF1                                ---------- ---------- ---------- ---------- ---------- ---------- ---------- ---------- ---------- ----------  1600
mani-IDR1                                ---------- ---------- ---------- ---------- ---------- ---------- ---------- ---------- ---------- ----------  1600
Metarhizium_anisopliae_TYPE_ARSEF_7487   AGTACTGCTG GCACCGGCCA ---------- ---------- CCGTCGGCCG TGGCGGAGGG TTACCCGGGA CCACAGTGCA TTTTTCC-GT TTTTTCCATT  1600
Metarhizium_anisopliae_ARSEF_1080        AGTACTGCTG GCACCGGCCA ---------- ---------- CCGTCGGCCG TGGCGGAGGG TTACCCGGGA CCACAGTGCA TTTTTCC-GT TTTTTCCATT  1600
Metarhizium_anisopliae_ARSEF_6347        AGTACTGCTG GCACCGGCCA ---------- ---------- CCGTCGGCCG TGGCGGAGGG TTACCCGGGA CCACAGTGCA TTTTTCC-GT TTTTTCCATT  1600
Metarhizium_anisopliae_ARSEF_7450        AGTACTGCTG GCACCGGCCA ---------- ---------- CCGTCGGCCG TGGCGGAGGG TTACCCGGGA CCACAGTGCA TTTTTCC-GT TTTTTCCATT  1600
Metarhizium_anisopliae_ARSEF_798         AGTACTGCTG GCACCGGCCA ---------- ---------- CCGTCGGCCG TGGCGGAGGG TTACCCGGGA CCACAGTGCA TTTTTCC-GT TTTTTCCATT  1600
Metarhizium_anisopliae_ART_2062          AGTACTGCTG GCACCGGCCA ---------- ---------- CCGTCGGCCG TGGCGGAGGG TTACCCGGGA CCACAGTGCA TTTTTCC-GT TTTTTCCATT  1600

mbru-IDF1                                ---------- ---------- ---------- ---------- ---------- ---------- ---------- ---------- ---------- ----------  1600
mbru-IDR1                                ---------- ---------- ---------- ---------- ---------- ---------- ---------- ---------- ---------- ----------  1600
Metarhizium_brunneum_TYPE_ARSEF_2107     ---------- ---------- ---------- ---------- ---------- ----GGAGGG TTACCCGGGA CCACGGTGCA TTTTTCC-GT TTTTTCCATT  1600
Metarhizium_brunneum_ARSEF_1066          ---------- -------TCA CCGTCGCGTG CACGCGGCTG CCGTCGGCCG TGGTGGAGGG TTACCCGGGA CCACGGTGCA TTTTTCC-GT TTTTTCCATT  1600
Metarhizium_brunneum_ARSEF_1278          AGCACTGCTG GTACCGGCCA CCGTCGCGTG CACGCGGCTG CCGTCGGCCG TGGTGGAGGG TTACCCGGGA CCACGGTGCA TTTTTCC-GT TTTTTCCATT  1600
Metarhizium_brunneum_ARSEF_3295          AGCACTGCTG GTACCGGCCA CCGCCGCGTG CACGCGGCTG CCGTCGGCCG TGGTGGAGGG TTACCCGGGA CCACGGTGCA TTTTTCCTTT TTTTTCCATT  1600
Metarhizium_brunneum_ARSEF_3826          ---------- -------TCA CCGTCGCGTG CACGCGGCTG CCGTCGGCCG TGGTGGAGGG TTACCCGGGA CCACGGTGCA TTTTTCC-GT TTTTTCCATT  1600
Metarhizium_brunneum_ARSEF_4152          AGTACTGCTG GTACCGGCCA CCGTCGCGTG CGCGCGGCTG CCGTCGGCCG TGGTGGAGGG CTACCCGGGA CCACGGTGCA ATTTTCC-TT TTTTTCCATT  1600
Metarhizium_brunneum_ARSEF_4179          AGTACTGCTG GTACCGGCCA CCGTCGCGTG CGCGCGGCTG CCGTCGGCCG TGGTGGAGGG CTACCCGGGA CCACGGTGCA ATTTTCC-TT TTTTTCCATT  1600
Metarhizium_brunneum_ARSEF_5198          ---------- -------TCA CCGTCGCGTG CACGCGGCTG CCGTCGGCCG TGGTGGAGGG TTACCCGGGA CCACGGTGCA TTTTTCC-GT TTTTTCCATT  1600
Metarhizium_brunneum_ARSEF_7524          ---------- -------TCA CCGTCGCGTG CACGCGGCTG CCGTCGGCCG TGGTGGAGGG TTACCCGGGA CCACGGTGCA TTTTTCC-GT TTTTTCCATT  1600
Metarhizium_brunneum_ARSEF_988           AGCACTGCTG GTACCGGCCA CCGCCGCGTG CACGCGGCTG CCGTCGGCCG TGGTGGAGGG TTACCCGGGA CCACGGTGCA TTTTTCCTTT TTTTTCCATT  1600
Metarhizium_brunneum_ART_2825            AGCACTGCTG GTACCGGCCA CCGTCGCGTG CACGCGGCTG CCGTCGGCCG TGGTGGAGGG TTACCCGGGA CCACGGTGCA TTTTTCC-TT TTTTTCCATT  1600

mpin-IDF1                                ---------- ---------- ---------- ---------- ---------- ---------- ---------- ---------- ---------- ----------  1600
mpin-IDR1                                ---------- ---------- ---------- ---------- ---------- ---------- ---------- ---------- ---------- ----------  1600
Metarhizium_pingshaense_TYPE_CBS_257.90  AGTACTGCTG GCACCGGCCA CCGTCGCGTG CACGCGGCTG CCGTCGGCCG TGGTAGAGGG TTACCCGGGA CCACGGTGCA TTTTTCC-GT TTTTTCCATT  1600
Metarhizium_pingshaense_ARSEF_2809       AGTACTGCTG GCACCGGCCA CCGTCGCGTG CACGCGGCTG CCGTCGGCCG TGGTAGAGGG TTACCCGGGA CCACGGTGCA TTTTTCC-GT TTTTTCCATT  1600
Metarhizium_pingshaense_ARSEF_3210       AGTACTGCTG GCACCGGCCA CCGTCGCGTG CACGCGGCTG CCGTCGGCCG TGGTAGAGGG TTACCCGGGA CCACGGTGCA TTTTTCC-GT TTTTTCCATT  1600
Metarhizium_pingshaense_ARSEF_4342       AGTACTGCTG GCACCGGCCA CCGTCGCGTG CACGCGGCTG CCGTCGGCCG TGGTAGAGGG TTACCCGGGA CCACGGTGCA TTTTTCC-GT TTTTTCCATT  1600
Metarhizium_pingshaense_ARSEF_7422       AGTACTGCTG GCACCGGCCA CCGTCGCGTG CACGCGGCTG CCGTCGGCCG TGGTAGAGGG TTACCCGGGA CCACGGTGCA TTTTTCC-GT TTTTTCCATT  1600
Metarhizium_pingshaense_ARSEF_7929       AGTACTGCTG GCACCGGCCA CCGTCGCGTG CACGCGGCTG CCGTCGGCCG TGGTAGAGGG TTACCCGGGA CCACGGTGCA TTTTTCC-GT TTTTTCCATT  1600

mrob-IDF1                                ---------- ---------- ---------- ---------- ---------- ---------- ---------- ---------- ---------- ----------  1600
mrob-IDR1                                ---------- ---------- ---------- ---------- ---------- ---------- ---------- ---------- ---------- ----------  1600
Metarhizium_robertsii_TYPE_ARSEF_2575    AGTACTGCTG GCACCGGCCA CCGTCGCGTG CACGCGGCTG CCGTCGGCCG TGGTGGAGGG TTACCCGGGA CCACGGTGCA TTTTTCC-GT TTTTTTCATT  1600
Metarhizium_robertsii_ARSEF_6472         AGTACTGCTG GCACCGGCCA CCGTCGCGTG CACGCGGCTG CCGTCGGCCG TGGTGGAGGG TTACCCGGGA CCACGGTGCA TTTTTCC-GT TTTTTTCATT  1600
Metarhizium_robertsii_ARSEF_727          AGTACTGCTG GCACCGGCCA CCGTCGCGTG CACGCGGCTG CCGTCGGCCG TGGTGGAGGG TTACCCGGGA CCACGGTGCA TTTTTCC-GT TTTTTTCATT  1600
Metarhizium_robertsii_ARSEF_7501         AGTACTGCTG GCACCGGCCA CCGTCGCGTG CACGCGGCTG CCGTCGGCCG TGGTGGAGGG TTACCCGGGA CCACGGTGCA TTTTTCC-GT TTTTTTCATT  1600
Metarhizium_robertsii_ARSEF_7532         AGTACTGCTG GCACCGGCCA CCGTCGCGTG CACGCGGCTG CCGTCGGCCG TGGTGGAGGG TTACCCGGGA CCACGGTGCA TTTTTCC-GT TTTTTTCATT  1600
Metarhizium_robertsii_ARSEF_8070         AGTACTGCTG GCACCGGCCA CCGTCGCGTG CACGCGGCTG CCGTCGGCCG TGGTGGAGGG TTACCCGGGA CCACGGTGCA TTTTTCC-GT TTTTTTCATT  1600
Metarhizium_robertsii_ARSEF_8685         AGTACTGCTG GCACCGGCCA CCGTCGCGTG CACGCGGCTG CCGTCGGCCG TGGTGGAGGG TTACCCGGGA CCACGGTGCA TTTTTCC-GT TTTTTTCATT  1600
```

```
mani-IDR1                                ---------- ---------- ---------- ---------- ---------- ---------- ---------- ---------- ---------- ----------  1700
Metarhizium_anisopliae_TYPE_ARSEF_7487   TTTT-CAAAA TCGACGACCA AAAAGTTGGT AGGTCTGGGT TGCCTTGCTG GCCAATAGAG ATTTGTAT-- ---------A GGGTAGGCCG GGGCAGGTAC  1700
Metarhizium_anisopliae_ARSEF_1080        TTTT-CAAAA TCGACGACCA AAAAGTTGGT AGGTCTGGGT TGCCTTGCTG GCCAATAGAG ATTTGTATGG CGCTACTATA GGGTAGGCTG -TGCCGGTAC  1700
Metarhizium_anisopliae_ARSEF_6347        TTTT-CAAAA TCGACGACCA AAAAGTTGGT AGGTCTGGGT TGCCTTGCTG GCCAATAGAG ATTTGTATGG CGCTACTATA GGGTAGGCTG -TGCCGGTAC  1700
Metarhizium_anisopliae_ARSEF_7450        TTTT-CAAAA TCGACGACCA AAAAGTTGGT AGGTCTGGGT TGCCTTGCTG GCCAATAGAG ATTTGTAT-- ---------A GGGTAGGCCG GGGCAGGTAC  1700
Metarhizium_anisopliae_ARSEF_798         TTTT-CAAAA TCGACGACCA AAAAGTTGGT AGGTCTGGGT TGCCTTGCTG GCCAATAGAG ATTTGTATGG CGCTACTATA GGGTAGGCTG -TGCCGGTAC  1700
Metarhizium_anisopliae_ART_2062          TTTT-CAAAA TCGACGACCA AAAAGTTGGT AGGTCTGGGT TGCCTTGCTG GCCAATAGAG ATTTGTAT-- ---------A GGGTAGGCCG GGGCAGGTAC  1700

mbru-IDF1                                ---------- ---------- ---------- ---------- ---------- ---------- ---------- ---------- ---------- ----------  1700
mbru-IDR1                                ---------- ---------- ---------- ---------- ---------- ---------- ---------- ---------- ---------- ----------  1700
Metarhizium_brunneum_TYPE_ARSEF_2107     TTTT-CAAAA TCGACGACCA AAAGGTTGGC AGGTCTGGGT TGCCTTGCTG GCCAATAGAG ATTTGTACGG CGCTACTATA GGGTAGG--- ----------  1700
Metarhizium_brunneum_ARSEF_1066          TTTT-CAAAA TCGACGACCA AAAGGTTGGT AGGTCTGGGT TGCCTTGCTG GCCAATAGAG ATTTGTACGG CGCTACTATA GGGTAGG--- ----------  1700
Metarhizium_brunneum_ARSEF_1278          TTTT-CAAAA TCGACGACCA AAAGGTTGGT AGGTCTGGGT TGCCTTGCTG GCCAATAGAG ATTTGTACGG CGCTACTATA GGGTAGG--- ----------  1700
Metarhizium_brunneum_ARSEF_3295          TTTT-CAAAA TCGACGACCA AAAGGTTGGT AGGTCTGGGT TGCCTGGCCG GCCAATAGAG ATTTGTACGG CGCTACTATA GGGTAGG--- ----------  1700
Metarhizium_brunneum_ARSEF_3826          TTTT-CAAAA TCGACGACCA AAAGGTTGGT AGGTCTGGGT TGCCTTGCTG GCCAATAGAG ATTTGTACGG CGCTACTATA GGGTAGG--- ----------  1700
Metarhizium_brunneum_ARSEF_4152          TTTT-CAAAA TCGACGACCA AAAGGTTGGT AGGTCTGGGT TGCCTTGCTG GCCAATAGAG ATTTGTACGG CGCTACTATA GGGTAGG--- ----------  1700
Metarhizium_brunneum_ARSEF_4179          TTTT-CAAAA TCGACGACCA AAAGGTTGGT AGGTCTGGGT TGCCTTGCTG GCCAATAGAG ATTTGTACGG CGCTACTATA GGGTAGG--- ----------  1700
Metarhizium_brunneum_ARSEF_5198          TTTT-CAAAA TCGACGACCA AAAGGTTGGT AGGTCTGGGT TGCCTTGCTG GCCAATAGAG ATTTGTACGG CGCTACTATA GGGTAGG--- ----------  1700
Metarhizium_brunneum_ARSEF_7524          TTTT-CAAAA TCGACGACCA AAAGGTTGGT AGGTCTGGGT TGCCTTGCTG GCCAATAGAG ATTTGTACGG CGCTACTATA GGGTAGG--- ----------  1700
Metarhizium_brunneum_ARSEF_988           TTTT-CAAAA TCGACGACCA AAAGGTTGGT AGGTCTGGGT TGCCTGGCCG GCCAATAGAG ATTTGTACGG CGCTACTATA GGGTAGG--- ----------  1700
Metarhizium_brunneum_ART_2825            TTTT-CAAAA TCGACGACCA AAAGGTTGGT AGGTCTGGGT TGCCTTGCTG GCCAATAGAG ATTTGTACGG CGCTACTATA GGGTAGG--- ----------  1700

mpin-IDF1                                ---------- ---------- ---------- ---------- ---------- ---------- ---------- ---------- ---------- ----------  1700
mpin-IDR1                                ---------- ---------- ---------- ---------- ---------- ---------- ---------- ---------- ---------- ----------  1700
Metarhizium_pingshaense_TYPE_CBS_257.90  TTTT-CAAAA TCGACGACCA AAAAGTTGGT AGGTCTGGGT TGCCTTGCTG GCCAATAGAG ATTTGTATGG CGCTACTATA GGGTAGG--- ----------  1700
Metarhizium_pingshaense_ARSEF_2809       TTTT-CAAAA TCGACGACCA AAAAGTTGGT AGGTCTGGGT TGCCTTGCTG GCCAATAGAG ATTTGTATGG CGCTACTATA GGGTAGG--- ----------  1700
Metarhizium_pingshaense_ARSEF_3210       TTTT-CAAAA TCGACGACCA AAAAGTTGGT AGGTCTGGGT TGCCTTGCTG GCCAATAGAG ATTTGTATGG CGCTACTATA GGGTAGG--- ----------  1700
Metarhizium_pingshaense_ARSEF_4342       TTTT-CAAAA TCGACGACCA AAAAGTTGGT AGGTCTGGGT TGCCTTGCTG GCCAATAGAG ATTTGTATGG CGCTACTATA GGGTAGG--- ----------  1700
Metarhizium_pingshaense_ARSEF_7422       TTTT-CAAAA TCGACGACCA AAAAGTTGGT AGGTCTGGGT TGCCTTGCTG GCCAATACAG ATTTGTATGG CGCTACTATA GGGTAAG--- ----------  1700
Metarhizium_pingshaense_ARSEF_7929       TTTT-CAAAA TCGACGACCA AAAAGTTGGT AGGTCTGGGT TGCCTTGCTG GCCAATAGAG ATTTGTATGG CGCTACTATA GGGTAGG--- ----------  1700

mrob-IDF1                                ---------- ---------- ---------- ---------- ---------- ---------- ---------- ---------- ---------- ----------  1700
mrob-IDR1                                ---------- ---------- ---------- ---------- ---------- ---------- ---------- ---------- ---------- ----------  1700
Metarhizium_robertsii_TYPE_ARSEF_2575    TTTTTCAAAA TCGACGACCA AAAAGTTGGT AGGTCTGGGT TGCCTTGCTG GCCAATAGAG ATTTGTATGG CGCTACTATA GGGTAGG--- ----------  1700
Metarhizium_robertsii_ARSEF_6472         TTTTTCAAAA TCGACGACCA AAAAGTTGGT AGGTCTGGGT TGCCTTGCTG GCCAATAGAG ATTTGTATGG CGCTACTATA GGGTAGG--- ----------  1700
Metarhizium_robertsii_ARSEF_727          TTTTTCAAAA TCGACGACCA AAAAGTTGGT AGGTCTGGGT TGCCTTGCTG GCCAATAGAG ATTTGTATGG CGCTACTATA GGGTAGG--- ----------  1700
Metarhizium_robertsii_ARSEF_7501         TTTTTCAAAA TCGACGACCA AAAAGTTGGT AGGTCTGGGT TGCCTTGCTG GCCAATAGAG ATTTGTATGG CGCTACTATA GGGTAGG--- ----------  1700
Metarhizium_robertsii_ARSEF_7532         TTTTTCAAAA TCGACGACCA AAAAGTTGGT AGGTCTGGGT TGCCTTGCTG GCCAATAGAG ATTTGTATGG CGCTACTATA GGGTAGG--- ----------  1700
Metarhizium_robertsii_ARSEF_8070         TTTTTCAAAA TCGACGACCA AAAAGTTGGT AGGTCTGGGT TGCCTTGCTG GCCAATACAG ATTTGTATGG CGCTACTATA GGGTAGG--- ----------  1700
Metarhizium_robertsii_ARSEF_8685         TTTTTCAAAA TCGACGACCA AAAAGTTGGT AGGTCTGGGT TGCCTTGCTG GCCAATAGAG ATTTGTATGG CGCTACTATA GGGTAGG--- ----------  1700
```

```
mani-IDF1                               ---------- ---------- ---------- ---------- ---------- ---------- ---------- ---------- ---------- ----------  1800
mani-IDR1                               ---------- ---------- ---------- ---------- ---------- ---------- ---------- ---------- ---------- ----------  1800
Metarhizium_anisopliae_TYPE_ARSEF_7487  TTGCAGTCGC ATACAAATTC TCTAGTAATT TGATGTACCT CCGCGCGAAT GGCAGTTTGG CAGGGTAGGT ACCGGGTAGG CTGGTGCAGG TACTTGCAGT  1800
Metarhizium_anisopliae_ARSEF_1080       TTGCTGTCGC ATACAAATTC CCTAGTAACT GGAAGTACCT C--------- -GCAGTTTGG TAGGGCAGGT ACCGGGTAGG CTG-TGCCGG TACTTGCTGT  1800
Metarhizium_anisopliae_ARSEF_6347       TTGCTGTCGC ATACAAATTC CCTAGTAACT GGAAGTACCT C--------- -GCAGTTTGG TAGGGCAGGT ACCGGGTAGG CTG-TGCCGG TACTTGCTGT  1800
Metarhizium_anisopliae_ARSEF_7450       TTGCAGTCGC ATACAAATTC TCTAGTAATT TGATGTACCT CCGCGCGAAT GGCAGTTTGG CAGGGTAGGT ACCGGGTAGG CTGGTGCAGG TACTTGCAGT  1800
Metarhizium_anisopliae_ARSEF_798        TTGCTGTCGC ATACAAATTC CCTAGTAACT GGAAGTACCT C--------- -GCAGTTTGG TAGGGCAGGT ACCGGGTAGG CTG-TGCCGG TACTTGCTGT  1800
Metarhizium_anisopliae_ART_2062         TTGCAGTCGC ATACAAATTC TCTAGTAATT TGATGTACCT CCGCGCGAAT GGCAGTTTGG CAGGGTAGGT ACCGGGTAGG CTGGTGCAGG TACTTGCAGT  1800

mbru-IDF1                               ---------- ---------- ---------- ---------- ---------- ---------- ---------- ---------- ---------- ----------  1800
mbru-IDR1                               ---------- ---------- ---------- ---------- ---------- ---------- ---------- ---------- ---------- ----------  1800
Metarhizium_brunneum_TYPE_ARSEF_2107    ---------- ---------- ---------- ---------- ---------- ---------- ---------T ACAGGGTAGG CTG-TGCCGG TACTTGCGGT  1800
Metarhizium_brunneum_ARSEF_1066         ---------- ---------- ---------- ---------- ---------- ---------- ---------T ACAGGGTAGG CTG-TGCCGG TACTTGCGGT  1800
Metarhizium_brunneum_ARSEF_1278         ---------- ---------- ---------- ---------- ---------- ---------- ---------T ACAGGGTAGG CTG-TGCCGG TACTTGCGGT  1800
Metarhizium_brunneum_ARSEF_3295         ---------- ---------- ---------- ---------- ---------- ---------- ---------T ACAGGGTAGG CTG-TGCCGG TACTTGCGGT  1800
Metarhizium_brunneum_ARSEF_3826         ---------- ---------- ---------- ---------- ---------- ---------- ---------T ACAGGGTAGG CTG-TGTCGG TACTTGCGGT  1800
Metarhizium_brunneum_ARSEF_4152         ---------- ---------- ---------- ---------- ---------- ---------- ---------T ACAGGGTAGG CTG-TGCCGG TACTTGCGGT  1800
Metarhizium_brunneum_ARSEF_4179         ---------- ---------- ---------- ---------- ---------- ---------- ---------T ACAGGGTAGG CTG-TGCCGG TACTTGCGGT  1800
Metarhizium_brunneum_ARSEF_5198         ---------- ---------- ---------- ---------- ---------- ---------- ---------T ACAGGGTAGG CTG-TGCCGG TACTTGCGGT  1800
Metarhizium_brunneum_ARSEF_7524         ---------- ---------- ---------- ---------- ---------- ---------- ---------T ACAGGGTAGG CTG-TGCCGG TACTTGCGGT  1800
Metarhizium_brunneum_ARSEF_988          ---------- ---------- ---------- ---------- ---------- ---------- ---------T ACAGGGTAGG CTG-TGCCGG TACTTGCGGT  1800
Metarhizium_brunneum_ART_2825           ---------- ---------- ---------- ---------- ---------- ---------- ---------T ACAGGGTAGG CTG-TGCCGG TACTTGCGGT  1800

mpin-IDF1                               ---------- ---------- ---------- ---------- ---------- ---------- ---------- ---------- ---------- ----------  1800
mpin-IDR1                               ---------- ---------- ---------- ---------- ---------- ---------- ---------- ---------- ---------- ----------  1800
Metarhizium_pingshaense_TYPE_CBS_257.90 ---------- ---------- ---------- ---------- ---------- ---------- ---------T ATAGGGTAGG CTG-TACCGG TACTTGCGGT  1800
Metarhizium_pingshaense_ARSEF_2809      ---------- ---------- ---------- ---------- ---------- ---------- ---------T ACAGGGTAGG CTG-TACCGG TACTTGCGGT  1800
Metarhizium_pingshaense_ARSEF_3210      ---------- ---------- ---------- ---------- ---------- ---------- ---------T ATAGGGTAGG CTG-TACCGG TACTTGCGGT  1800
Metarhizium_pingshaense_ARSEF_4342      ---------- ---------- ---------- ---------- ---------- ---------- ---------T GTAGGGTAGG CTG-TACCGG TACTTGCGGT  1800
Metarhizium_pingshaense_ARSEF_7422      ---------- ---------- ---------- ---------- ---------- ---------- ---------C ATAGGGTAGG CTG-TACCGG TACTTGCGGT  1800
Metarhizium_pingshaense_ARSEF_7929      ---------- ---------- ---------- ---------- ---------- ---------- ---------T ATAGGGTAGG CTG-TACCGG TACTTGCGGT  1800

mrob-IDF1                               ---------- ---------- ---------- ---------- ---------- ---------- ---------- ---------- ---------- ----------  1800
mrob-IDR1                               ---------- ---------- ---------- ---------- ---------- ---------- ---------- ---------- ---------- ----------  1800
Metarhizium_robertsii_TYPE_ARSEF_2575   ---------- ---------- ---------- ---------- ---------- ---------- ---------T ATAGGGTAGG CTG-TGCCGG TACTTGCGGT  1800
Metarhizium_robertsii_ARSEF_6472        ---------- ---------- ---------- ---------- ---------- ---------- ---------T ATAGGGTAGG CTG-TGCCGG TACTTGCGGT  1800
Metarhizium_robertsii_ARSEF_727         ---------- ---------- ---------- ---------- ---------- ---------- ---------T ATAGGGTAGG CTG-TGCCGG TACTTGCGGT  1800
Metarhizium_robertsii_ARSEF_7501        ---------- ---------- ---------- ---------- ---------- ---------- ---------T ATAGGGTAGG CTG-TGCCGG TACTTGCGGT  1800
Metarhizium_robertsii_ARSEF_7532        ---------- ---------- ---------- ---------- ---------- ---------- ---------T ATAGGGTAGG CTG-TGCCGG TACTTGCGGT  1800
Metarhizium_robertsii_ARSEF_8070        ---------- ---------- ---------- ---------- ---------- ---------- ---------T ATAGGGTAGG CTG-TGCCGG TACTTGCGGT  1800
Metarhizium_robertsii_ARSEF_8685        ---------- ---------- ---------- ---------- ---------- ---------- ---------T ATAGGGTAGG CTG-TGCCGG TACTTGCGGT  1800
```

```
mani-IDF1                                ---------- ---------- ---------- ---------- ----------  1850
mani-IDR1                                ---------- ---------- ---------- ---------- ----------  1850
Metarhizium_anisopliae_TYPE_ARSEF_7487   TGCATACAAA TTCTCTAGTA ACTGGAAGTA CCCCCGCGCG CGGGCAACTT  1850
Metarhizium_anisopliae_ARSEF_1080        CGCATACAAA TTCCCTAGTA ACTGGAAGTA CCCCCGCGCG CGGGCAACTT  1850
Metarhizium_anisopliae_ARSEF_6347        CGCATACAAA TTCCCTAGTA ACTGGAAGTA CCCCCGCGCG CGGGCAACTT  1850
Metarhizium_anisopliae_ARSEF_7450        TGCATACAAA TTCTCTAGTA ACTGGAAGTA CCCCCGCGCG CGGGCAACTT  1850
Metarhizium_anisopliae_ARSEF_798         CGCATACAAA TTCCCTAGTA ACTGGAAGTA CCCCCGCGCG CGGGCAACTT  1850
Metarhizium_anisopliae_ART_2062          TGCATACAAA TTCTCTAGTA ACTGGAAGTA CCCCCGCGCG CGGGCAACTT  1850

mbru-IDF1                                ---------- ---------- ---------- ---------- ----------  1850
mbru-IDR1                                ---------- ---------- ---------- ---------- ----------  1850
Metarhizium_brunneum_TYPE_ARSEF_2107     CGCACACAAA T-CCCTAGCA GCCTGAAGTA CCCCCGCGCG CGGGCAACTT  1850
Metarhizium_brunneum_ARSEF_1066          CGCACACAAA T-CCCTAGCA GCCTGAAGTA CCCCCGCGCG CGGGCAACTT  1850
Metarhizium_brunneum_ARSEF_1278          CGCACACAAA T-CCCTAGCA GCCCGAAGTA CCCCCGCGCG CGGGCAAGTT  1850
Metarhizium_brunneum_ARSEF_3295          CGCACACAAA T-CCCTAGCA GCCTGAAGTA CCCCCGCGCG CGGGCAGCTT  1850
Metarhizium_brunneum_ARSEF_3826          CGCACACAAA T-CCCTAGCA GCCTGAAGTA CCCCCGCGCG CGGGCAACTT  1850
Metarhizium_brunneum_ARSEF_4152          CGCACACAAA T-CCCTAGCA GCCTGAAGTA CCCCCGCGCG CGGGCAGCTT  1850
Metarhizium_brunneum_ARSEF_4179          CGCACACAAA T-CCCTAGCA GCCTGAAGTA CCCCCGCGCG CGGGCAGCTT  1850
Metarhizium_brunneum_ARSEF_5198          CGCACACAAA T-CCCTAGCA GCCTGAAGTA CCCCCGCGCG CGGGCAACTT  1850
Metarhizium_brunneum_ARSEF_7524          CGCACACAAA T-CCCTAGCA GCCTGAAGTA CCCCCGCGCG CGGGCAACTT  1850
Metarhizium_brunneum_ARSEF_988           CGCACACAAA T-CCCTAGCA GCCTGAAGTA CCCCCGCGCG CGGGCAGCTT  1850
Metarhizium_brunneum_ART_2825            CGCACACAAA T-CCCTAGCA GCCTGAAGTA CCCCCGCGC- -GGGCAGCCT  1850

mpin-IDF1                                ---------- ---------- ---------- ---------- ----------  1850
mpin-IDR1                                ---------- ---------- ---------- ---------- ----------  1850
Metarhizium_pingshaense_TYPE_CBS_257.90  CGCACACAAA T-CCCTAGCA GCCTGGAGTA CCCCCGCGCG CGGGCAACTT  1850
Metarhizium_pingshaense_ARSEF_2809       CGCACACAAA T-CCCTAGCA GCCTGGAGTA CCCCCGCGCG CGGGCAACTT  1850
Metarhizium_pingshaense_ARSEF_3210       CGCACACAAA T-CCCTAGCA GCCTGGAGTA CCCCCGCGCG CGGGCAACTT  1850
Metarhizium_pingshaense_ARSEF_4342       CGCACACAAA T-CCCTAGCA GCCTGGAGTA CCCCCGCGCG CGGGCAACTT  1850
Metarhizium_pingshaense_ARSEF_7422       CGCACACAAA T-CCCTAGCA GCCTGGAGTA CCCCCGCGCG CGGGCAACTT  1850
Metarhizium_pingshaense_ARSEF_7929       CGCACACAAA T-CCCTAGCA GCCTGGAGTA CCCCCGCGCG CGGGCAACTT  1850

mrob-IDF1                                ---------- ---------- ---------- ---------- ----------  1850
mrob-IDR1                                ---------- ---------- ---------- ---------- ----------  1850
Metarhizium_robertsii_TYPE_ARSEF_2575    CGCACACAAA T-CCCTAGCA GCCTGAAGTA CCCCCGCGCG CGGGCAACTT  1850
Metarhizium_robertsii_ARSEF_6472         CGCACACAAA T-CCCTAGCA GCCTGAAGTA CCCCCGCGCG CGGGCAACTT  1850
Metarhizium_robertsii_ARSEF_727          CGCACACAAA T-CCCTAGCA GCCTGAAGTA CCCCCGCGCG CGGGCAACTT  1850
Metarhizium_robertsii_ARSEF_7501         CGCACACAAA T-CCCTAGCA GCCTGAAGTA CCCCCGCGCG CGGGCAACTT  1850
Metarhizium_robertsii_ARSEF_7532         CGCACACAAA T-CCCTAGCA GCCTGAAGTA CCCCCGCGCG CGGGCAACTT  1850
Metarhizium_robertsii_ARSEF_8070         CGCACACAAA T-CCCTAGCA GCCTGAAGTA CCCCCGCGCG CGGGCAACTT  1850
Metarhizium_robertsii_ARSEF_8685         CGCACACAAA T-CCCTAGCA GCCTGAAGTA CCCCCGCGCG CGGGCAACTT  1850
```

Supplementary Figure S6. Alignment of ribosomal intergenic spacer (rIGS) sequences used for diagnostic primer design. Diagnostic primer pairs are indicated by colors: mani-IDF1/R1 (blue), mbru-IDF1/R1 (green), mpin-IDF1/R1 (red), mrob-IDF1/R1 (yellow).
