# Supplementary material for: Species Discrimination within the Metarhizium PARB Clade: Ribosomal Intergenic Spacer (rIGS)-Based Diagnostic PCR and Single Marker Taxonomy
Source: J Fungi (Basel). 2023 Oct 8;9(10):996. doi: 10.3390/jof9100996 (PMC10607842; doi:10.3390/jof9100996)
Supplement: Supplementary file 1 [file jof-09-00996-s001.zip › Suppl Figure S7.pdf]

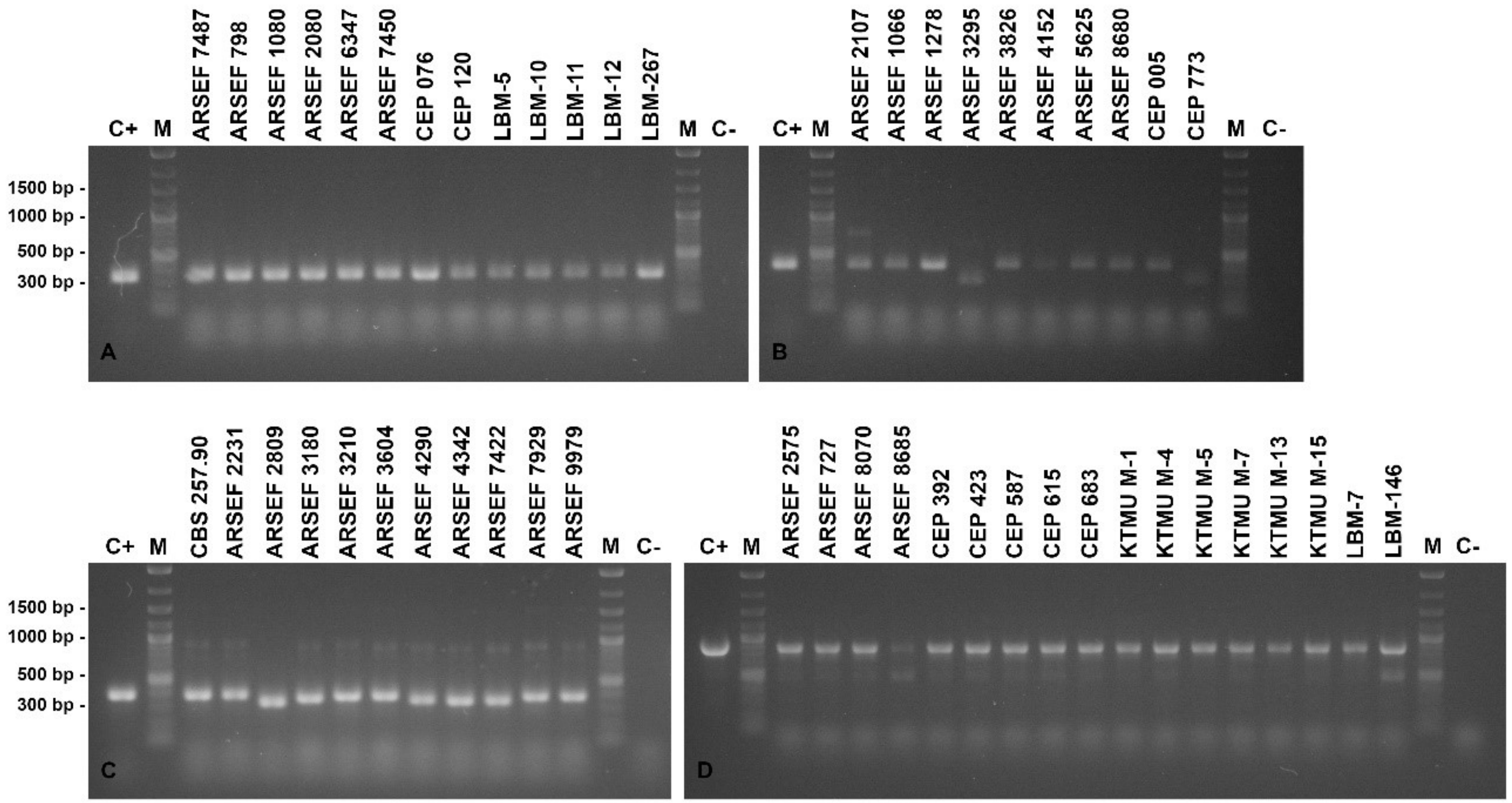

Suppl. Figure S7. Agarose gel electrophoresis of multiplexed diagnostic PCR reactions using simultaneously the four PARB species-discriminating primer pairs mani-IDF1/R1, mbru-IDF1/R1, mpin-IDF1/R1, and mrob-IDF1/R1 with template DNA of both the reference and validation strain sets, sorted by *Metarhizium* species: *M. anisopliae* (**A**), *M. brunneum* (**B**), *M. pinghaense* (**C**), and *M. robertsii* (**D**). The length of relevant signals in the size standard is indicated at the left gel margins. Lane labels on top of gels indicate the *Metarhizium*

strain or isolate; “**M**” denotes the size standard, “**C-**“ the negative (no template) control, “**C+**” un-multiplexed PCR reactions with respective type strain DNA and the cognate diagnostic primer pair only as species-specific positive control.
