# Supplementary material for: Species Discrimination within the Metarhizium PARB Clade: Ribosomal Intergenic Spacer (rIGS)-Based Diagnostic PCR and Single Marker Taxonomy
Source: J Fungi (Basel). 2023 Oct 8;9(10):996. doi: 10.3390/jof9100996 (PMC10607842; doi:10.3390/jof9100996)
Supplement: Supplementary file 1 [file jof-09-00996-s001.zip › Suppl Figure S8.pdf]

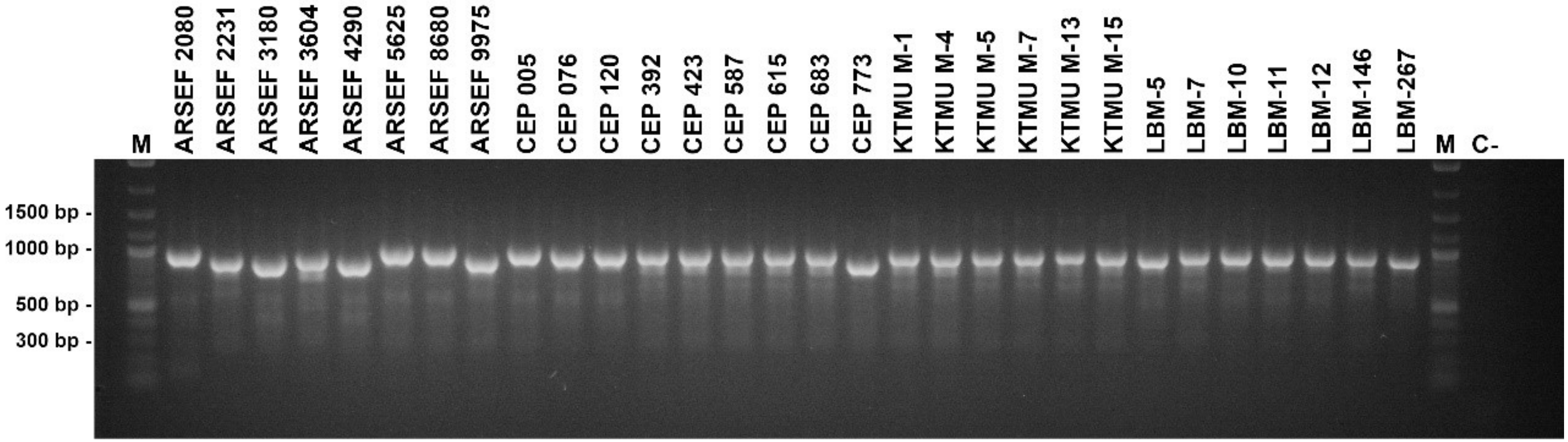

Suppl. Figure S8. Agarose gel electrophoresis of PCR reactions from template DNA of the validation strain set amplifying the rIGS-ID800 sequence with primer pair Migs1-F1 / Migs850-R1. The length of relevant signals in the size standard is indicated at the left margin. Lane labels on top of the picture indicate the *Metarhizium* strain or isolate; "**M**" denotes the size standard, "**C-**" the negative (no template) control.
