# Supplementary material for: Species Discrimination within the Metarhizium PARB Clade: Ribosomal Intergenic Spacer (rIGS)-Based Diagnostic PCR and Single Marker Taxonomy
Source: J Fungi (Basel). 2023 Oct 8;9(10):996. doi: 10.3390/jof9100996 (PMC10607842; doi:10.3390/jof9100996)
Supplement: Supplementary file 1 [file jof-09-00996-s001.zip › Suppl Figure S9.pdf]

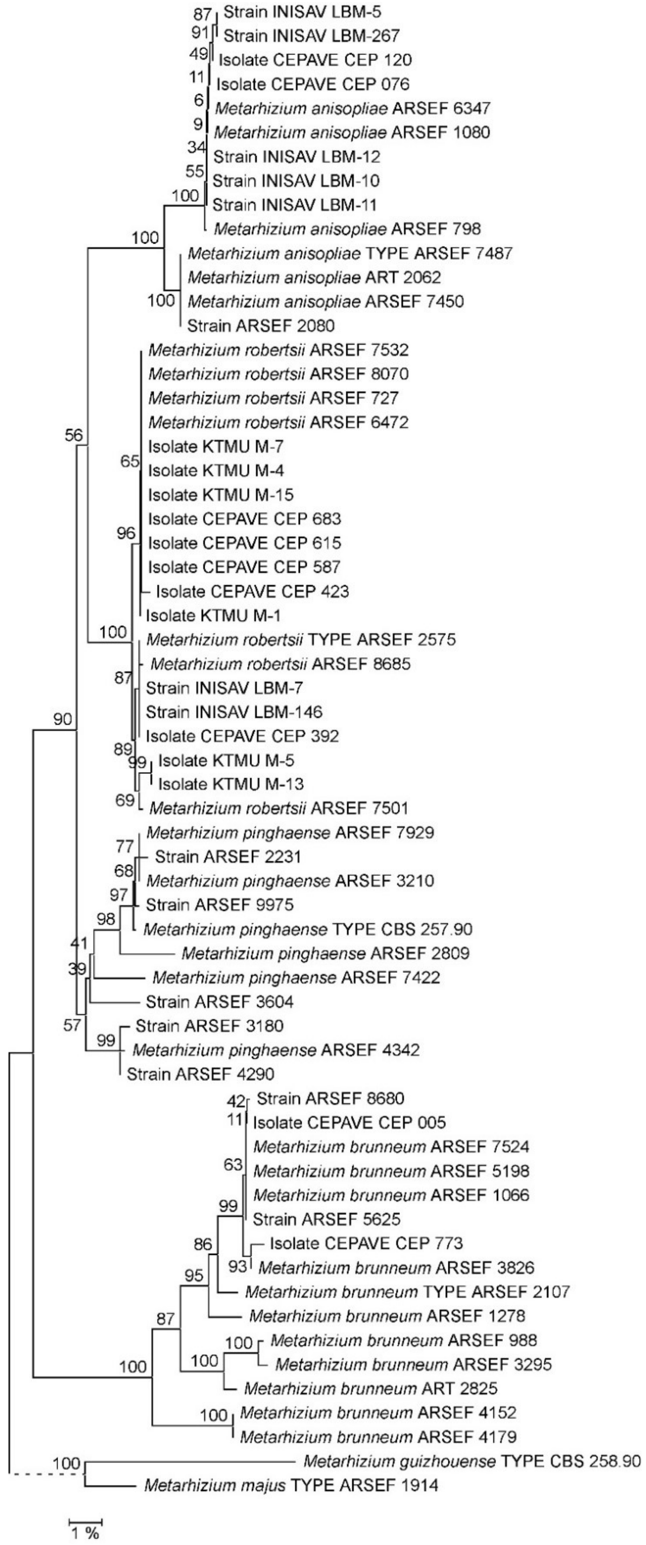

Supplementary Figure S7. Neighbor-Joining (NJ) phylogeny of *Metarhizium* fungi as reconstructed from 5TEF sequences. Terminal branches are labelled by genus, species and strain designations; “TYPE” denotes the nomenclatural type strain of a species. Numbers on branches indicate bootstrap support percentages. The size bar corresponds to 1 % sequence divergence; branches drawn as dashed lines are not to scale. The 5TEF sequence from the *M. acridum* type strain has been used as outgroup.
