# Supplementary material for: Species Discrimination within the Metarhizium PARB Clade: Ribosomal Intergenic Spacer (rIGS)-Based Diagnostic PCR and Single Marker Taxonomy
Source: J Fungi (Basel). 2023 Oct 8;9(10):996. doi: 10.3390/jof9100996 (PMC10607842; doi:10.3390/jof9100996)
Supplement: Supplementary file 1 [file jof-09-00996-s001.zip › Suppl Table S1.pdf]

Suppl. Table S1. Reference data set of *Metarhizium* strains and marker sequences used for diagnostic PCR development. "-T" following a strain designation denotes a nomenclatural type strain.

Strain Designation	Genus, Species	rIGS	5TEF	EF1A	RPB1	RPB2	Geographic Origin	Original Host, Isolation Source	References
ARSEF 7486 - T	*Metarhizium acridum*	NA	EU248845	EU248845	EU248897	EU248925	Niger	Orthoptera	[24]
ARSEF 798	*Metarhizium anisopliae*	OR296677	KX342696	OR296642	OR296653	OR296664	Colombia	*Aeneolamia varia*	[36], this study
ARSEF 1080	*Metarhizium anisopliae*	OR296678	KX342698	OR296643	OR296654	OR296665	USA	*Helicoverpa zea*	[36], this study
ARSEF 6347	*Metarhizium anisopliae*	MH604976	EU248881	OR296644	OR296655	OR296666	Colombia	*Hemiptera*	[24,37], this study
ARSEF 7450	*Metarhizium anisopliae*	MH604977	EU248852	EU248852	EU248904	EU248932	Australia	Coleoptera	[24,37]
ARSEF 7487 - T	*Metarhizium anisopliae*	MH604974	DQ463996	DQ463996	DQ468355	DQ468370	Ethiopia	Orthoptera	[29,37]
ART 2062	*Metarhizium anisopliae*	MH604975	NA	NA	NA	NA	India	NA	[37]
ARSEF 988	*Metarhizium brunneum*	MH604982	KX342758	EU248890	NA	NA	Japan	*Bombyx mori*	[24,36,37]
ARSEF 1066	*Metarhizium brunneum*	OR296679	KX342763	OR296645	OR296656	OR296667	Switzerland	*Melolontha melolontha*	[36], this study
ARSEF 1278	*Metarhizium brunneum*	OR296680	KX342772	OR296646	OR296657	OR296668	Canada	soil	[36], this study
ARSEF 2107 - T	*Metarhizium brunneum*	MH604983	EU248855	EU248855	EU248907	EU248935	USA	Coleoptera	[24,37]
ARSEF 3295	*Metarhizium brunneum*	OR296681	KX342759	OR296647	OR296658	OR296669	Mexico	*Anticarsia gemmatalis*	[36], this study

ARSEF 3826	*Metarhizium brunneum*	MH604984	KX342769	OR296648	OR296659	OR296670	USA	*Ochlerotatus triseriatus*	[36,37], this study
ARSEF 4152	*Metarhizium brunneum*	MH604984	KX342757	EU248853	EU248905	EU248933	Australia	soil	[24,36,37]
ARSEF 4179	*Metarhizium brunneum*	MH604986	EU248854	EU248854	EU248906	EU248934	Australia	soil	[24,37]
ARSEF 5198	*Metarhizium brunneum*	MH604987	EU248876	NA	NA	NA	Germany	Coleoptera	[24,37]
ARSEF 7524	*Metarhizium brunneum*	MH604988	NA	NA	NA	NA	Switzerland	*Agriotes sp.* larva	[37]
ART 2825	*Metarhizium brunneum*	MH604989	NA	KR706488	NA	NA	Switzerland	*Agriotes obscurus*	[37,48]
CBS 258.90 - T	*Metarhizium guizhouense*	MH604996	EU248862	EU248862	EU248914	EU248942	China	Lepidoptera	[24,37], this study
ESALQ 1638	*Metarhizium humberi*	JACEFI010000005.1	KP027955	JACEFI010000008.1	JACEFI010000006.1	JACEFI010000009.1	Brazil	soil	[34]
IP 1	*Metarhizium humberi*	NA	JQ061188	MH837571	MH837553	MH837562	Brazil	soil	[35,49]
IP 46 - T	*Metarhizium humberi*	NA	JQ061205	MH837574	MH837556	MH837565	Brazil	soil	[35,49]
IP 86	*Metarhizium humberi*	NA	JQ061186	MH837576	MH837558	MH837567	Brazil	soil	[35,49]
IP 151	*Metarhizium humberi*	NA	JQ061208	MH837579	MH837561	MH837570	Brazil	soil	[49]
ARSEF 1914 - T	*Metarhizium majus*	MH604993	EU248868	EU248868	EU248920	EU248948	Phillipines	Coleoptera	[24,37]
ARSEF 2809	*Metarhizium pinghaense*	OR296682	KX342712	OR296649	OR296660	OR296671	Philippines	soil	[36], this study
ARSEF 3210	*Metarhizium pinghaense*	MH604981	DQ463995	DQ463995	DQ468354	DQ468369	India	Coleoptera	[29,37]
ARSEF 4342	*Metarhizium pinghaense*	MH604980	EU248851	EU248851	EU248903	EU248931	Solomon Islands	Coleoptera	[24,37]

ARSEF 7422	*Metarhizium pinghaense*	OR296683	KX342706	OR296650	OR296661	OR296672	Australia	Coleoptera	[36], this study
ARSEF 7929	*Metarhizium pinghaense*	MH604979	EU248847	EU248847	EU248899	OR296675	Australia	Isoptera	[24,37], this study
CBS 257.90 - T	*Metarhizium pinghaense*	MH604978	EU248850	EU248850	EU248902	OR296676	China	Coleoptera	[24,37], this study
ARSEF 727	*Metarhizium robertsii*	MH604970	DQ463994	DQ463994	DQ468353	DQ468368	Brazil	Orthoptera	[29,37]
ARSEF 2575 - T	*Metarhizium robertsii*	MH604971	KX342729	JELW01000003.1	JELW01000006.1	JELW01000009.1	USA	*Curculio caryae*	[36,37]
ARSEF 6472	*Metarhizium robertsii*	MH604972	KX342740	EU248884	NA	NA	USA	*Dectes texanus*	[24,36,37]
ARSEF 7501	*Metarhizium robertsii*	MH604973	KX342741	EU248849	EU248901	EU248929	Australia	Coleoptera	[24,36,37]
ARSEF 7532	*Metarhizium robertsii*	MH604969	NA	NA	NA	NA	Switzerland	*Melolontha melolontha*	[37]
ARSEF 8070	*Metarhizium robertsii*	OR296684	KX342750	OR296651	OR296662	OR296673	Australia	mound material of *Nasutitermes exitiosus*	[36], this study
ARSEF 8685	*Metarhizium robertsii*	OR296685	FJ229494	OR296652	OR296663	OR296674	Canada	Soil	[24], this study
