# Supplementary material for: Species Discrimination within the Metarhizium PARB Clade: Ribosomal Intergenic Spacer (rIGS)-Based Diagnostic PCR and Single Marker Taxonomy
Source: J Fungi (Basel). 2023 Oct 8;9(10):996. doi: 10.3390/jof9100996 (PMC10607842; doi:10.3390/jof9100996)
Supplement: Supplementary file 1 [file jof-09-00996-s001.zip › Suppl Table S2.pdf]

Suppl. Table S2. Validation data set of *Metarhizium* strains and marker sequences. "M." abbreviates "Metarhizium".

Strain / Isolate	Previous Identification	Identification by			5TEF	rIGS-ID800	Geographic Origin	Original Host, Isolation Source	References
		diagnostic PCR	rIGS-ID800	5TEF					
ARSEF 2080	*M. anisopliae sensu stricto*	*M. anisopliae*	*M. anisopliae*	*M. anisopliae*	KX342700	OR400501	Indonesia	*Nilaparvata lugens*	[36], this study
ARSEF 2231	*M. pinghaense*	*M. pinghaense*	*M. pinghaense*	*M. pinghaense*	KX342723	OR400502	India	*Zygogramma bicolorata*	[36], this study
ARSEF 3180	*M. pinghaense*	*M. pinghaense*	*M. pinghaense*	*M. pinghaense*	KX342704	OR400503	Philippines	Soil	[36], this study
ARSEF 3604	*M. pinghaense*	*M. pinghaense*	*M. pinghaense*	*M. pinghaense*	KX342708	OR400504	Papua New Guinea	*Scapanes australis*	[36], this study
ARSEF 4290	*M. pinghaense*	*M. pinghaense*	*M. pinghaense*	*M. pinghaense*	KX342705	OR400505	Solomon Islands	Coleoptera	[36], this study
ARSEF 5625	*M. brunneum*	*M. brunneum*	*M. brunneum*	*M. brunneum*	KX342765	OR400506	Finland	Soil	[36], this study
ARSEF 8680	*M. anisopliae sensu lato*	*M. brunneum*	*M. brunneum*	*M. brunneum*	FJ229493	OR400507	Canada	Soil	[24], this study
ARSEF 9975	*Metarhizium sp.*	*M. pinghaense*	*M. pinghaense*	*M. pinghaense*	MK156097	OR400508	U.S.A.	Soil	[50], this study
CEPAVE CEP 005	*M. anisopliae sensu lato*	*M. brunneum*	*M. brunneum*	*M. brunneum*	OR441043	OR441065	Argentina, Buenos Aires, Berisso	*Aedes crinifer* [Diptera: Culicidae]	this study
CEPAVE CEP 076	*M. anisopliae sensu lato*	*M. anisopliae*	*M. anisopliae*	*M. anisopliae*	OR441044	OR441066	Argentina, Buenos Aires, Los Hornos	*Kanaima fluvialis* [Hemiptera: Cercopidae]	this study
CEPAVE CEP 120	*M. anisopliae sensu lato*	*M. anisopliae*	*M. anisopliae*	*M. anisopliae*	OR441045	OR441067	Argentina, Buenos Aires, Abasto	*Kanaima fluvialis* [Hemiptera: Cercopidae]	this study
CEPAVE CEP 392	*M. anisopliae sensu lato*	*M. robertsii*	*M. robertsii*	*M. robertsii*	OR441046	OR441068	Argentina, San Juan, Est. Exp. P	Soil	this study
CEPAVE CEP 423	*M. anisopliae sensu lato*	*M. robertsii*	*M. robertsii*	*M. robertsii*	OR441047	OR441069	Argentina, La Plata	Soil	this study
CEPAVE CEP 587	*M. anisopliae sensu lato*	*M. robertsii*	*M. robertsii*	*M. robertsii*	OR441048	OR441070	Argentina, Buenos Aires, Parque Pereyra Iraola	Soil	this study
CEPAVE CEP 615	*M. anisopliae sensu lato*	*M. robertsii*	*M. robertsii*	*M. robertsii*	OR441049	OR441071	Argentina	Soil	this study

CEPAVE CEP 683	*M. anisopliae sensu lato*	*M. robertsii*	*M. robertsii*	*M. robertsii*	OR441050	OR441072	Argentina, La Plata, Est. Exp. J. Hirschhorn	Soil	this study
CEPAVE CEP 773	*M. anisopliae sensu lato*	*M. brunneum*	*M. brunneum*	*M. brunneum*	OR441051	OR441073	Argentina, Salta, Piedra del Molino	Soil	this study
INISAV LBM-5	*M. anisopliae*	*M. anisopliae*	*M. anisopliae*	*M. anisopliae*	MZ329397	OR441058	Cuba	undetermined Hemipteran insect	[6], this study
INISAV LBM-7	*M. anisopliae sensu lato*	*M. robertsii*	*M. robertsii*	*M. robertsii*	OR423400	OR441059	Cuba	undetermined Homopteran insect	this study
INISAV LBM-10	*M. anisopliae*	*M. anisopliae*	*M. anisopliae*	*M. anisopliae*	MZ329398	OR441060	Cuba	undetermined Hemipteran insect	[6], this study
INISAV LBM-11	*M. anisopliae*	*M. anisopliae*	*M. anisopliae*	*M. anisopliae*	MZ329399	OR441061	Cuba, Havana	*Mocis latipes* (Lepidoptera: Erebidae)	[6], this study
INISAV LBM-12	*M. anisopliae*	*M. anisopliae*	*M. anisopliae*	*M. anisopliae*	MZ329400	OR441062	Cuba	*Corcyra cephalonica* (Lepidoptera: Pyralidae)	[6], this study
INISAV LBM-146	*M. robertsii*	*M. robertsii*	*M. robertsii*	*M. robertsii*	MZ329401	OR441063	Cuba	Leaf hopper (Hemiptera: Cicadellidae)	[6], this study
INISAV LBM-267	*M. anisopliae*	*M. anisopliae*	*M. anisopliae*	*M. anisopliae*	MZ329402	OR441064	Cuba	Leaf hopper (Hemiptera: Cicadellidae)	[6], this study
KTMU M-01	*M. anisopliae sensu lato*	*M. robertsii*	*M. robertsii*	*M. robertsii*	OR441052	OR441074	Kyrgyzstan, Chemche village	Soil	this study
KTMU M-04	*M. anisopliae sensu lato*	*M. robertsii*	*M. robertsii*	*M. robertsii*	OR441053	OR441053	Kyrgyzstan, Syrt village	Soil	this study
KTMU M-05	*M. anisopliae sensu lato*	*M. robertsii*	*M. robertsii*	*M. robertsii*	OR441054	OR441076	Kyrgyzstan, Molo village	Soil	this study
KTMU M-07	*M. anisopliae sensu lato*	*M. robertsii*	*M. robertsii*	*M. robertsii*	OR441055	OR441077	Kyrgyzstan, Zhetigen village	Soil	this study
KTMU M-13	*M. anisopliae sensu lato*	*M. robertsii*	*M. robertsii*	*M. robertsii*	OR441056	OR441078	Kyrgyzstan, Krugowoi	Soil	this study
KTMU M-15	*M. anisopliae sensu lato*	*M. robertsii*	*M. robertsii*	*M. robertsii*	OR441057	OR441079	Kyrgyzstan, Kolmo village	Soil	this study
