# Supplementary material for: Species Discrimination within the Metarhizium PARB Clade: Ribosomal Intergenic Spacer (rIGS)-Based Diagnostic PCR and Single Marker Taxonomy
Source: J Fungi (Basel). 2023 Oct 8;9(10):996. doi: 10.3390/jof9100996 (PMC10607842; doi:10.3390/jof9100996)
Supplement: Supplementary file 1 [file jof-09-00996-s001.zip › Suppl Table S3.pdf]

Suppl Table S3. Preparative PCR primers used and reaction-specific parameters applied in this study.

Primer Designation	Primer Sequence (5′ => 3′)	Annealing Temperature (°C)	Elongation Time (sec)	References
EF1T	ATGGGTAAGGARGACAAGAC	64	60	[29]
EF2T	GGAAGTACCAGTGATCATGTT			
EF1A-983F	GCYCCYGGHCAYCGTGAYTTYAT	52	90	[53]
EF1A-2218R	ATGACACCRACRGCRACRGTYTG			
EF1A-1567R	ACHGTRCCRATACCACCSATCTT	sequencing primer		[53]
EF1A-1577F	CARGAYGTBTACAAGATYGGTGG	sequencing primer		[6]
RPB1Af	GARTGYCCDGGDCAYTTYGG	50	90	[54]
RPB1Cr	CCNGCDATNTCRTTRTCCATRTA			
RPB2-5f	GAYGAYMGWGATCAYTTYGG	50	120	[52]
RPB2-7r	CCCATRGCTTGYTTRCCCAT			
RPB2-6f	TGGGGKWTGGTYTGYCCTGC	sequencing primer		[51]
RPB2-6r	GCAGGRCARACCAWMCCCCA	sequencing primer		
Migs1-F1	CCTTGTTGTTACGATCTGCTGAG	55	90	this study
Migs850-R1	TAAASAAGCAGCCTACCCTAAAGC			this study
Migs1-F2	CTGAGGGTTAGCCGTTCTTCG	64	120	this study
Migs 1200R	CCAGCAGAGAAATCCCCTCAC			this study
Migs 1100F	AGAGRGAGGGCTGGACACGCG	64	120	this study
Migs 2000R	GGCTCAATCGATGCAGCTCGGCGAG			this study
